# The quorum sensing peptide EntF* promotes colorectal cancer metastasis in mice: a new factor in the host-microbiome interaction

**DOI:** 10.1186/s12915-022-01317-z

**Published:** 2022-06-27

**Authors:** Evelien Wynendaele, Nathan Debunne, Yorick Janssens, Anton De Spiegeleer, Frederick Verbeke, Liesa Tack, Sophie Van Welden, Evy Goossens, Daniel Knappe, Ralf Hoffmann, Christophe Van De Wiele, Debby Laukens, Peter Van Eenoo, Lars Vereecke, Filip Van Immerseel, Olivier De Wever, Bart De Spiegeleer

**Affiliations:** 1 Drug Quality and Registration Group, Faculty of Pharmaceutical Sciences, Ghent University, Ghent, Belgium; 2 Department of Internal Medicine and Pediatrics, Faculty of Medicine and Health Sciences, Ghent University, Ghent, Belgium; 3 Department of Pathology, Bacteriology and Poultry diseases, Faculty of Veterinary Medicine, Ghent University, Ghent, Belgium; 4 Center of Biotechnology and Biomedicine, Faculty of Chemistry and Mineralogy, Universität Leipzig, Leipzig, Germany; 5 Department of Diagnostic Sciences, Faculty of Medicine and Health Sciences, Ghent University, Ghent, Belgium; 6 Department of Rheumatology, Faculty of Medicine and Health Sciences, Ghent, Belgium; 7 Department of Human Structure and Repair, Faculty of Medicine and Health Sciences, Ghent University, Ghent, Belgium

**Keywords:** Quorum sensing peptides, Microbiota, Colorectal cancer metastasis, Orthotopic mice model, LC-MS

## Abstract

**Background:**

Colorectal cancer, one of the most common malignancies worldwide, is associated with a high mortality rate, mainly caused by metastasis. Comparative metagenome-wide association analyses of healthy individuals and cancer patients suggest a role for the human intestinal microbiota in tumor progression. However, the microbial molecules involved in host-microbe communication are largely unknown, with current studies mainly focusing on short-chain fatty acids and amino acid metabolites as potential mediators. Quorum sensing peptides are not yet considered in this context since their presence in vivo and their ability to affect host cells have not been reported so far.

**Results:**

Here, we show that EntF*, a metabolite of the quorum sensing peptide EntF produced by *Enterococcus faecium*, is naturally present in mice bloodstream. Moreover, by using an orthotopic mouse model, we show that EntF* promotes colorectal cancer metastasis in vivo, with metastatic lesions in liver and lung tissues. In vitro tests suggest that EntF* regulates E-cadherin expression and consequently the epithelial-mesenchymal transition, via the CXCR4 receptor. In addition, alanine-scanning analysis indicates that the first, second, sixth, and tenth amino acid of EntF* are critical for epithelial-mesenchymal transition and tumor metastasis.

**Conclusion:**

Our work identifies a new class of molecules, quorum sensing peptides, as potential regulators of host-microbe interactions. We prove, for the first time, the presence of a selected quorum sensing peptide metabolite in a mouse model, and we demonstrate its effects on colorectal cancer metastasis. We believe that our work represents a starting point for future investigations on the role of microbiome in colorectal cancer metastasis and for the development of novel bio-therapeutics in other disease areas.

**Supplementary Information:**

The online version contains supplementary material available at 10.1186/s12915-022-01317-z.

## Background

Colorectal cancer (CRC) is the third most commonly occurring malignancy worldwide and is associated with a high mortality rate, mainly caused by metastasis (mCRC). Primary CRC originates from epithelial cells that line the gastrointestinal tract, usually through an adenoma-carcinoma sequence: normal colorectal epithelium transforms into an adenoma and eventually into an invasive and metastatic tumor. During the initial steps of the metastatic process, epithelial CRC cells switch towards a mesenchymal phenotype, known as epithelial-to-mesenchymal transition (EMT) [1, 2]. Although the role of genetic predisposition is clear in some cases, a strong association of CRC with diet and lifestyle has been demonstrated [3]. Moreover, inflammation is believed to play a role in CRC development either as a promoting condition, as shown by colitis-associated colorectal cancers (CAC) in patients with inflammatory bowel disease (IBD), or as a consequence, in patients with sporadic CRC [4].

Over the last decade, an increasing amount of evidence suggested that human intestinal microbiota may play a role in CRC [5–7]. For example, a high abundance of *Enterococcus*, *Escherichia*, and *Fusobacterium* species was observed in the feces of patients suffering from intestinal disorders, including CRC and Crohn’s disease [8–11]. The causative links between gut microbiota and CRC development/progression are not clear to date, and current investigations focus on bacterial-derived compounds such as short-chain fatty acids and amino acid-derived amines [12].

Quorum sensing peptides are traditionally regarded as intra- and inter-bacterial communication molecules. However, given their structural variety and co-evolution with the host, these bacterial metabolites may also affect host physiology. Indeed, different quorum sensing peptides were previously found to influence the behavior of host cells, including brain, muscle, and cancer cells (CRC and breast cancer) [13–16]. In CRC, specific microbial quorum sensing peptides were found to promote tumor cell invasion and angiogenesis in vitro, suggesting potential pro-metastatic effects of these peptides. However, the presence of quorum sensing peptides in biofluids has not been unambiguously demonstrated. In fact, only indirect evidence of the presence of an uncharacterized quorum sensing peptide in the stool of patients with *Clostridium difficile* infection was reported so far [17].

In this study, we focus on *Enterococcus faecium*, one of the most abundant *Enterococcus* species in the human intestinal microbiota [18–21], which synthesizes the enterocin induction factor, i.e., the precursor of the EntF quorum sensing peptide (AGTKPQGKPASNLVECVFSLFKKCN). This peptide acts as a communication signal, regulating the production of enterocin A and B toxins, which inhibit the growth of similar or closely related bacterial strains [22–27]. Interestingly, it was previously discovered that EntF*, a metabolite of the quorum sensing peptide EntF, could promote CRC tumor cell invasion in vitro [13].

Here, we demonstrate the presence of the EntF* peptide in mice biofluids, along with the EntF*-induced pro-metastatic effect in vivo*.* Together, these results indicate a potential role of quorum sensing peptides in the host-microbiome interaction and CRC tumorigenesis.

## Results

### Presence of EntF* in mice biofluids

**Fig. 1:**
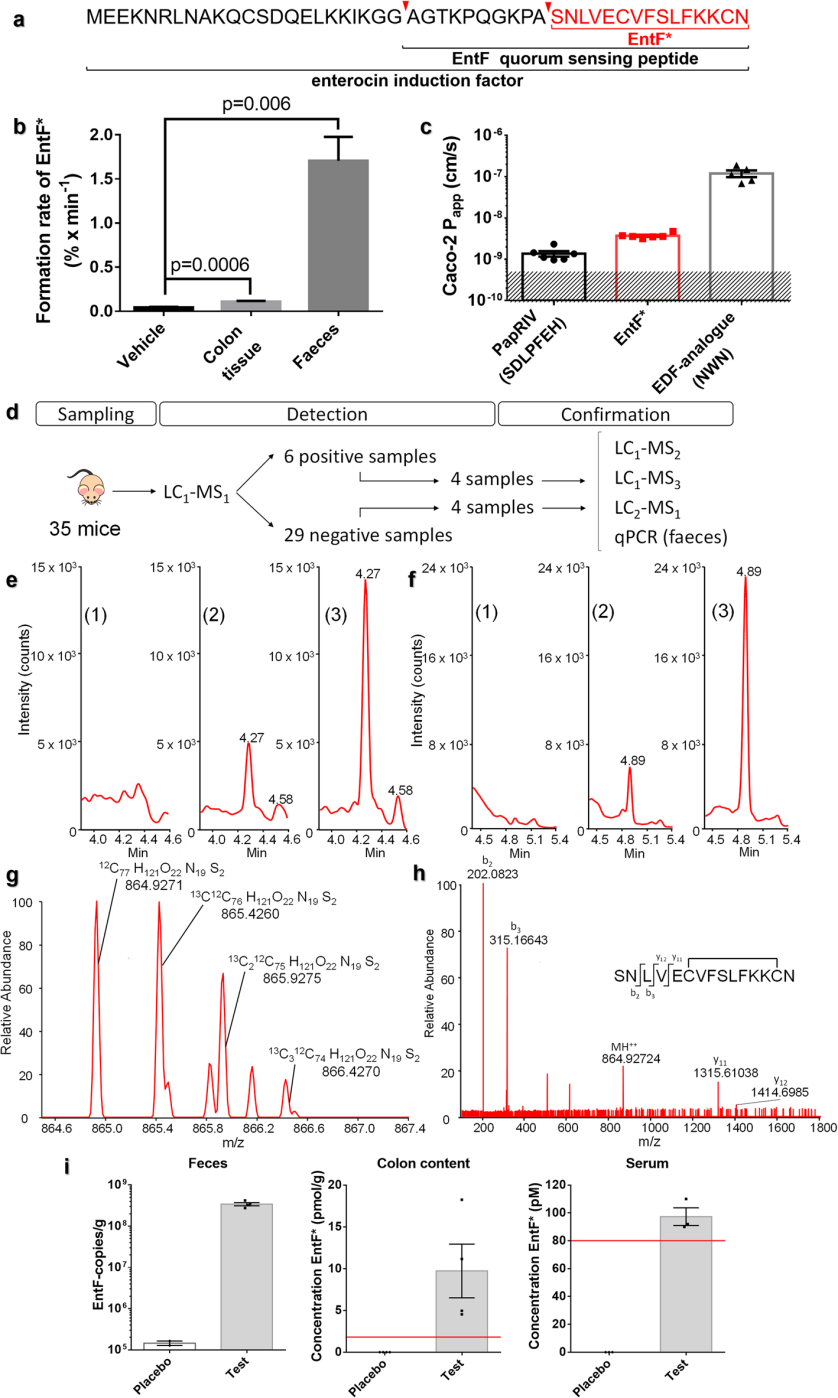
In vitro formation and in vivo presence of the EntF* metabolite. **a** Sequences of the enterocin induction factor pro-peptide, mature quorum sensing peptide EntF, and its metabolite EntF*. **b** In vitro formation rate of EntF* from EntF in colon (*n* = 7) and feces (*n* = 4) homogenates. Bars represent the mean formation rate ± SEM from independent experiments. Statistically significant differences were determined by a Mann-Whitney *U* test with indicated *p*-values. **c** Apparent permeability coefficients (*P*_app_) of PapRIV, EntF*, and EDF-analog in Caco-2 cells. Bars represent mean P_app_ values ± SEM (*n* = 6 independent experiments); the shaded area represents the limit of detection. **d** Flow chart displaying the experimental design stages, from serum sampling to peptide detection and further confirmation of EntF* presence in vivo. Different LC-MS methods: LC_1_-MS_1_, reversed-phase ultra-high-performance liquid chromatography (RP-UPLC) using triple quadrupole (TQ) in MRM mode; LC_1_-MS_2_, high-resolution quadrupole time-of-flight; LC_1_-MS_3_, high-resolution quadrupole-orbitrap; LC_2_-MS_1_, HILIC-amide UPLC using TQ in MRM mode. qPCR was performed on feces sample of mice from the same set to demonstrate the presence of EntF-encoding DNA sequences from *E. faecium*. **e** Chromatographic profiles of (1) negative serum sample, (2) positive serum sample, (3) serum sample from EntF*-treated mice. Chromatographic profiles were obtained using RP-UPLC with detection by electrospray ionization mass spectrometry (ESI-MS) using TQ in MRM mode (*m/z* = 865 ➔ 202.08 + 315.17). **f** Chromatographic profiles of (1) negative serum sample, (2) positive serum sample, (3) serum sample from EntF*-treated mice. Chromatographic profiles were obtained using HILIC amide UPLC with detection by ESI-MS using TQ in MRM mode (*m/z* = 865 ➔ 202.08 + 315.17). **g** Isotopic distribution of the double charged EntF* measured in a positive serum sample using RP-UPLC with detection by ESI-MS using quadrupole-orbitrap. **h** High-resolution tandem mass spectrum of EntF* with characteristic fragments, using RP-UPLC with detection by Q-TOF. **i** In vivo presence of EntF* in gnotobiotic mice treated with EntF-producing bacterial strains. Number of EntF DNA copies per gram of feces measured four days after treatment with placebo (300 μL BHI medium) (limit of detection: 10^5^ copies/g) (left). EntF* concentration in colon content. No EntF* was detected in the placebo group (the red line indicates the limit of detection) (middle). EntF* concentration in serum content. No EntF* was detected in the placebo group (the red line indicates the limit of detection) (right)

To demonstrate that the native EntF peptide can be metabolized into EntF* in mice, we carried out in vitro metabolization studies (Fig. 1a) on feces and colonic tissue homogenates. Our experiments show that the formation rate of the 15-mer peptide EntF* (SNLVECVFSLFKKCN) is 1.71 ± 0.27 % min^−1^ (mean ± SEM; *n* = 4) and 0.11 ± 0.01 % min^−1^ (mean ± SEM; *n* = 7) in feces and colonic tissues, respectively (Fig. 1b, Additional files 1 and 2). Similarly to other quorum sensing peptides [14], EntF* is able to cross the intestinal barrier in vitro with an apparent permeability coefficient of 3.67 ± 0.22 × 10^−9^ cm s^−1^ (mean ± SEM; *n* = 6) in Caco-2 monolayer permeability assays (Fig. 1c, Additional files 1 and 3).

To prove the presence of EntF* in vivo, serum samples from 35 healthy, non-manipulated C57BL/6 mice (age 5–18 months) were collected and analyzed (Fig. 1d, Additional files 1 and 4) by reverse phase ultra-high performance liquid chromatography tandem quadrupole mass spectrometry (RP-UPLC-TQ-MS; LC_1_-MS_1_). Notably, we improved the detection protocol to avoid carry-over and adsorption and to maximize selectivity and sensitivity. To achieve these goals, we (1) developed an adequate sample preparation protocol using a novel bovine serum albumin (BSA)-based anti-adsorption solution [28] together with a combination of solvent/acid/heat sample treatment followed by solid phase extraction and (2) set up optimal MS detection parameters, including the selection of quantifier (b2: m/z = 202.08) and qualifier (b3: m/z = 315.17) ions. This method was thoroughly tested and validated (Additional file 5: Fig. S1, Additional file 6). The amount of EntF* detected in the serum of six mice was above 100 pM (limit of quantification, LOQ; Fig. 1e). Taking into account all samples, including those with EntF* values < LOQ (considered as zero), we obtained an EntF* mean value of 329 ± 150 pM (mean ± SEM; *n* = 35; Additional file 5: Table S1). Serum samples from four EntF*-positive and four EntF*-negative samples (i.e., above and below LOQ, respectively) were analyzed using additional methods (Fig. 1f–h, Additional files 7, 8 and 9). The presence and identity of EntF* were confirmed through the isotopic distribution of the doubly charged EntF* precursor ion (Fig. 1 g) and by the presence of fragment ions y_11_ (*m/z* = 1315.61) and y_12_ (*m/z* = 1414.69) in the four positive serum samples (Fig. 1 h, Additional file 5: Table S1). To demonstrate the presence of EntF-encoding bacterial DNA, quantitative real-time PCR was performed on fecal samples obtained from the same mice used in the biochemical analysis: EntF-encoding DNA sequences were found in all positive samples (Additional file 5: Fig. S2a, Table S1, Additional file 1).

To confirm the bacterial origin of EntF*, EntF-producing *E. faecium* strains were administered via oral gavage to germ-free mice. Four days after the colonization of the mice gastrointestinal system, EntF* was observed in the colon content of the conditioned mice at a concentration of 10 ± 3.2 pmol/g (mean ± SEM; *n* = 4), while it was below the limit of detection (LoD_EntF*_ = 1.9 pmol/g) in the placebo and control groups. EntF* was also detected in the serum of the test group (Fig. 1i, Additional file 10). In addition, standard BLAST protein searches indicated the absence of peptide sequences similar to EntF* in the murine genome (maximum sequence alignment of 67%), supporting the microbial origin of the EntF* peptide found in vivo.

### In vitro activity and molecular targets of EntF*

**Fig. 2:**
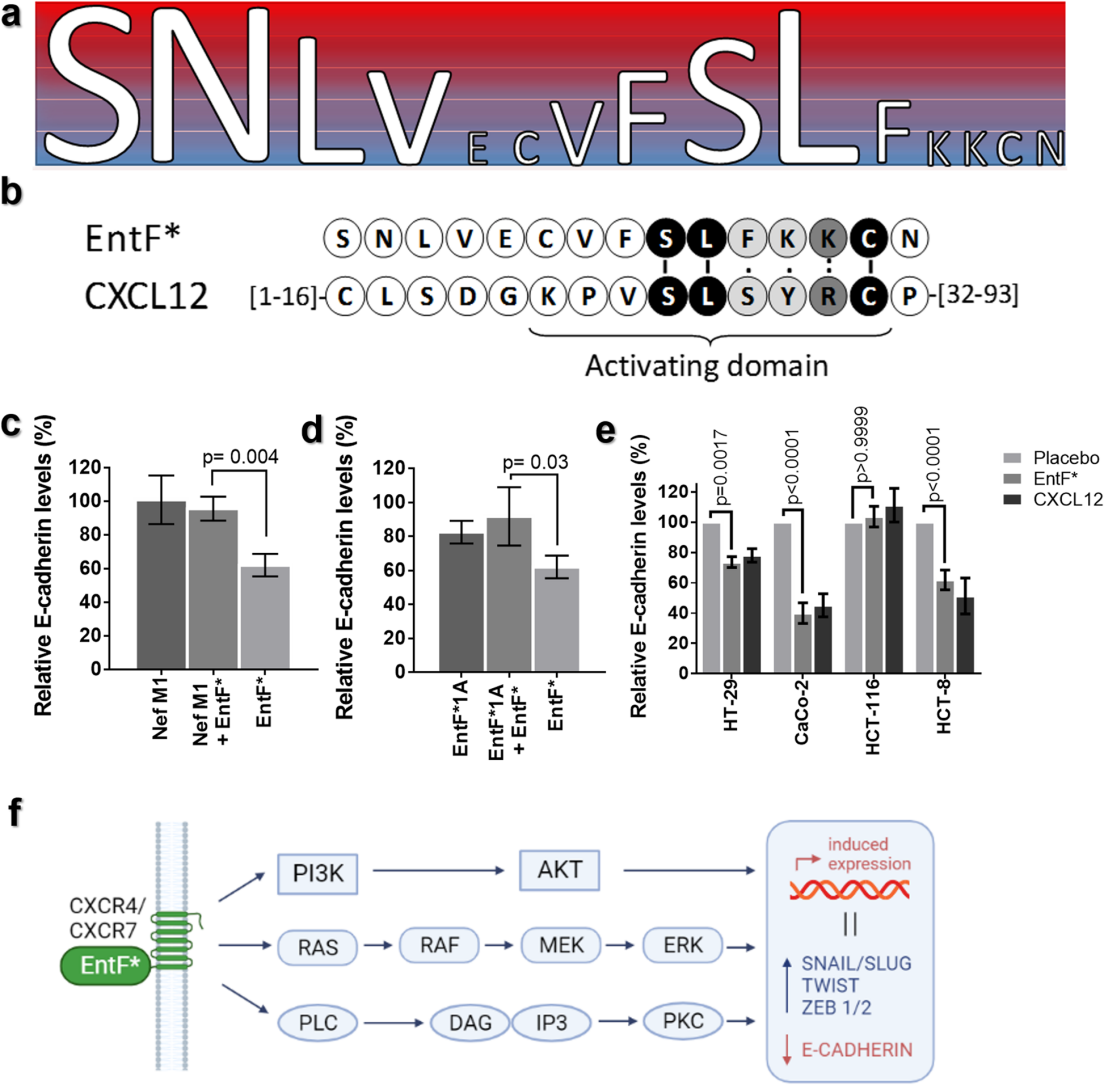
In vitro activity of the EntF* peptide. **a** The relative importance of the amino acids of EntF* on E-cadherin expression ranked in five different classes. Ranking (blue to red: increasing significance) was performed using the Fisher’s LSD *p*-values, which was confirmed using the Jenks natural breaks algorithm. Based on ranking, it is likely that the first, second and tenth amino acid of EntF* are the most important residues for EntF* activity. **b** Alignment between EntF* and the active domain of CXCL12. Black amino acids with a line indicate a match. Dark grey amino acids with two dots mark a similarity between the two residues. Light grey amino acids with one dot are not similar, but no gap is formed. **c** Antagonistic effects of Nef-M1 on EntF*-mediated E-cadherin downregulation in HCT-8 cells (EntF* *n* = 15; Nef-M1 *n* = 6; EntF* + Nef-M1 *n* = 6). Statistically significant differences were determined by a one-sided student's t test. **d** Antagonistic effects of EntF*1A on EntF*-mediated E-cadherin downregulation in HCT-8 cells (EntF* *n* = 15; EntF*1A *n* = 18; EntF* + EntF*1A *n* = 6). Statistically significant differences were determined by a one-sided Student’s *t* test. **e** Effect of EntF* on E-cadherin expression. A significant decrease in E-cadherin levels following EntF* or CXCL12 treatments was observed in HT-29 (*n* = 12), Caco-2 (*n* = 6) and HCT-8 (*n* = 15) cells. Statistically significant differences were determined by one-way ANOVA test. **f** Proposed E-cadherin-regulating pathway for EntF*. Schematic representation of the CXCR4 receptor and its signaling pathways, leading to the activation of EMT transcription factors, followed by the downregulation of E-cadherin expression (see also Additional file 5: Fig. S5)

Western blot analysis showed that EntF* and some alanine- or D-amino acid derived analogs affect the expression of E-cadherin, which is linked to the EMT of cancer cells (Fig. 2a, Additional file 5: Fig. S3-S4, Additional files 11 and 12). Western blot quantifications showed a significant decrease in E-cadherin expression following EntF* treatment (− 38% with respect to control). When the first, second, or tenth amino acid of EntF* was replaced by an alanine residue, the reduction in E-cadherin expression was significantly flattened out. These results indicate that amino acids in position 1, 2, and 10 are important for the EMT-promoting effects of EntF*. The other residues contribute to a much lesser extent, as determined by the Fisher’s LSD test, and further confirmed using the Jenks natural breaks algorithm. Replacing the sixth amino acid of EntF* with the corresponding D-amino acid isomer restored E-cadherin expression to placebo levels. These results suggest that the stereochemical configuration of the sixth amino acid of EntF* is important for its EMT-promoting effects.

To further investigate the mechanism by which EntF* promotes metastasis, we reasoned that EntF*, by mimicking endogenous ligands of the E-cadherin-regulating pathway, may interact with these receptors, thereby provoking signaling pathways that lead to an increased expression of EMT transcription factors; this is then followed by an upregulation of N-cadherin and subsequent downregulation of E-cadherin expression. To this end, we searched for similarities between EntF* and active domains of 13 different natural ligands (Additional file 5: Fig. S5 and Table S2); the selected ligands are known to trigger signaling pathways that lead to an increased expression of EMT transcription factors, thus resulting in the transcriptional downregulation of E-cadherin (Additional file 5: Fig. S5 and Table S2). According to the respective alignment with the activating domain of receptors’ natural ligands and to the relative importance of the aligned amino acids, three receptors were selected as the most likely targets of EntF*: C-X-C chemokine receptor type 4 (CXCR4; alignment score = 27), interleukin 6 (IL-6; alignment score = 21), and vascular endothelial growth factor (VEGF; alignment score = 19). Interestingly, for the activation of the CXCR4 receptor, a nine N-terminal amino acid stretch (KPVSLSYRC; amino acids 22–30) of the endogenous ligand C-X-C motif chemokine 12 (CXCL12) is required [29]; alignment of this CXCL12 activating domain and EntF* also shows a good similarity (Fig. 2b).

To investigate whether EntF* interacts with the CXCR4 receptor, we analyzed by Western blot the effect of the Nef-M1 (an antagonist of CXCL12) on the EntF*-mediated E-cadherin downregulation in HCT-8 CRC cells. Interestingly, co-treatment of HCT-8 CRC cells with EntF* and Nef-M1 restored E-cadherin expression to control levels (Fig. 2c, Additional file 13): E-cadherin levels increased from 62% (EntF* only) to 94% (EntF* + Nef-M1) (Cohen’s *d* = 1.4). These results suggest an interaction between EntF* and the CXCL12/CXCR4 pathway in EMT promotion and tumor metastasis (based on the E-cadherin-regulating pathway, Additional file 5: Fig. S5).

Interestingly, the modified peptide EntF*1A, where the serine amino acid at position 1 is replaced by an alanine residue, also displayed antagonistic activity towards the EntF*/CXCR4 interaction: E-cadherin relative expression levels increased from 62% to 92% (Cohen’s *d* = 0.9) when EntF*1A was added to EntF*-treated cells (Fig. 2d).

Additionally, treatment of HT-29 and Caco-2 CRC cells with EntF* also resulted in a statistically significant reduction of E-cadherin expression. Similar effects on E-cadherin expression were also observed when HT-29 and Caco-2 CRC cells were treated with the endogenous CXCL12 peptide. Interestingly, when CXCR4-negative HCT-116 cells were treated with EntF* or with the CXCL-12 ligand, no decrease in E-cadherin expression was observed in response to either peptide (Fig. 2e, Additional file 14). Together, these data suggest that EntF* exerts its functions by interacting with the CXCR4-mediated signaling pathway, ultimately leading to E-cadherin downregulation (Fig. 2f). The lack of response in HCT-116 cells suggests that CXCR4 receptor is likely the sole molecular target of the EntF* peptide.

### In vivo pro-metastatic properties of EntF*

**Fig. 3:**
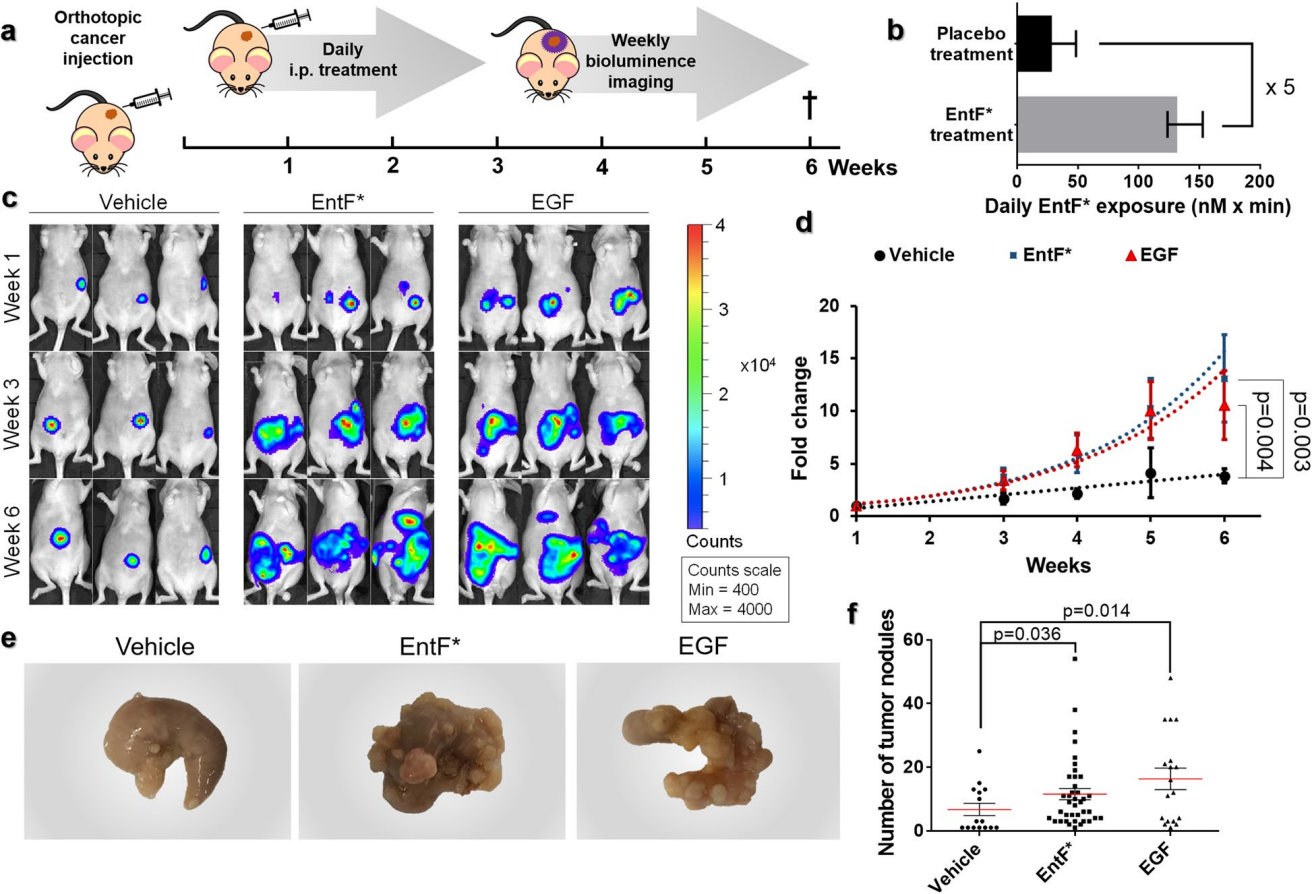
Metastasis-inducing effect of EntF* in an orthotopic mouse model of colorectal cancer. **a** Schematic timeline of the experimental setup. Female Swiss nu/nu mice were orthotopically injected with 1 × 10^6^ luciferase-transfected HCT-8 cells at the age of 5 weeks. During the following 6 weeks, mice were daily i.p. injected with 10^2^ nmol kg^−1^ EntF* (*n* = 38), PBS control (vehicle, *n* = 17), or 0.1 mg kg^−1^ EGF positive control (*n* = 18). Bioluminescent imaging was performed weekly to determine cancer progression. After 6 weeks, mice were euthanized and the cecum, liver and lungs collected. **b** Graph representing the average daily exposure of placebo-treated (black; *n* = 65) and EntF*-treated (gray; *n* = 14) female Swiss nu/nu mice. In the placebo group, mice with an EntF* serum concentration below the limit of detection (100 pM) were counted as zero. The daily exposure after i.p. injection of 10^2^ nmol kg^−1^ EntF* is 5 times higher than the naturally occurring EntF* levels. **c** Representative images of bioluminescence activity in vehicle, EntF*-treated, and EGF-treated mice. Mice were i.p. injected with 150 mg kg-1 luciferin and imaged after 10 min in supine position. **d** Tumor growth curves derived from the quantification of bioluminescence in vehicle, EntF*-treated, and EGF-treated mice. Based on linear regression slope comparison, EntF* and EGF treatment resulted in a significant increase in tumor growth compared to the vehicle. Data represent the mean relative increase in bioluminescence ± SEM. **e** Representative pictures of vehicle, EntF*-treated, and EGF-treated ceca at the end of the experiment. **f** Number of tumor nodules in untreated (vehicle), EntF*-treated, and EGF-treated ceca determined by macroscopic inspection of tissues. Data represent the mean nodule number ± SEM. Statistically significant differences were determined by a Mann-Whitney U test with indicated *p*-values

**Fig. 4:**
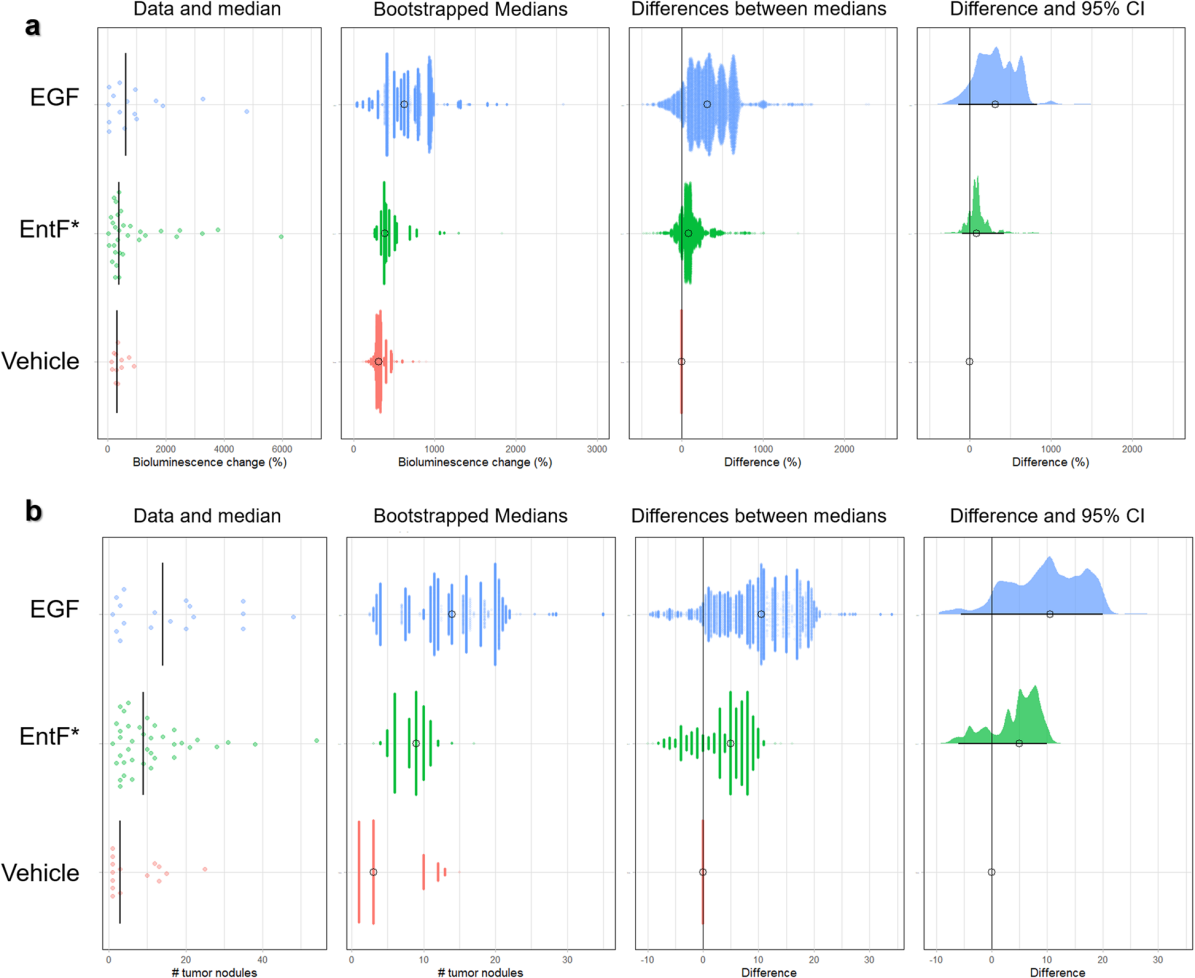
Effect size for bioluminescence and number of nodules in the cecum of the orthotopic mouse model after a 6-week treatment. **a** After a 6-week treatment, an effect size of 79% increase in bioluminescence for EntF* compared to the placebo was observed, while for the positive control EGF, a median effect size of 316% was obtained. When calculating the effect size according to Hedges’ *G* values, a medium to large effect was observed for both EntF* and EGF treatment groups, compared to the placebo group. **b** After a 6-week treatment, a 3-fold increase in the number of nodules on the cecum was observed for EntF* compared to the placebo PBS, while for the positive control EGF, a 4.5-fold increase was obtained. When calculating the effect size according to Hedges’ *G* values, a medium and large effect was observed for the EntF* and EGF treatment groups, respectively, compared to the placebo group [Fig. prepared in R-script: https://github.com/JoachimGoedhart/PlotsOfDifferences]

Pro-metastatic properties of EntF* were determined using an orthotopic mouse model of colorectal cancer. Luciferase-transfected HCT-8 cells were treated for 5 days with phosphate-buffered saline (PBS) vehicle, 10^2^ nM EntF*, or 0.1 μg mL^−1^ transforming growth factor α (TGFα, positive control). On the sixth day, luciferase-transfected CRC cells were orthotopically injected into the cecal wall of 6-week-old female Swiss nu/nu mice. Subsequently mice were treated once a day intraperitoneally with PBS vehicle (*n* = 17), 10^2^ nmol kg^−1^ EntF* (*n* = 38), or 10^2^ μg kg^−1^ Epidermal Growth Factor (EGF, positive control; *n* = 18) (Fig. 3a). To calculate EntF* daily exposure after intraperitoneal (i.p.) injections (10^2^ nmol kg^−1^; *n* = 14), in vivo serum kinetic profiles of EntF* were determined (see Materials and Methods). Additionally, endogenous EntF* levels in untreated female Swiss nu/nu mice (*n* = 65) were assessed. These analyses concluded that daily EntF* injections resulted in five times higher EntF* serum levels compared to the endogenous (natural) exposure in untreated mice, thus demonstrating the biological relevance of this experimental set-up (Fig. 3b, Additional file 15). Bioluminescent imaging of mice was performed weekly to monitor tumor growth (Fig. 3c, Additional file 16). Over a period of 6 weeks, EntF* caused a statistically significant increase in luciferase activity compared to the vehicle (*p* = 0.003). This increase was similar to what was observed in EGF-treated controls (*p* = 0.432; Fig. 3d). Our results show, after a 6-week treatment, an effect size of 79% increase in bioluminescence for EntF* compared to the placebo, ranging from -92%, a relatively small negative association, to a 417% increase, a substantial positive association (Fig. 4a). For the EGF control, a median effect size of 316% was scored, ranging from − 145 to 826% (Fig. 4a). The tumor-promoting activity of EntF* was further confirmed by macroscopically assessing the number of nodules on the cecum of treated mice: EntF*-treated samples showed a 3-fold increase in the number of nodules compared to PBS vehicle (*p* = 0.036) (Fig. 3e, f), whereas EGF-treated samples showed a 4.5-fold increase compared to control (Fig. 4b).

**Fig. 5:**
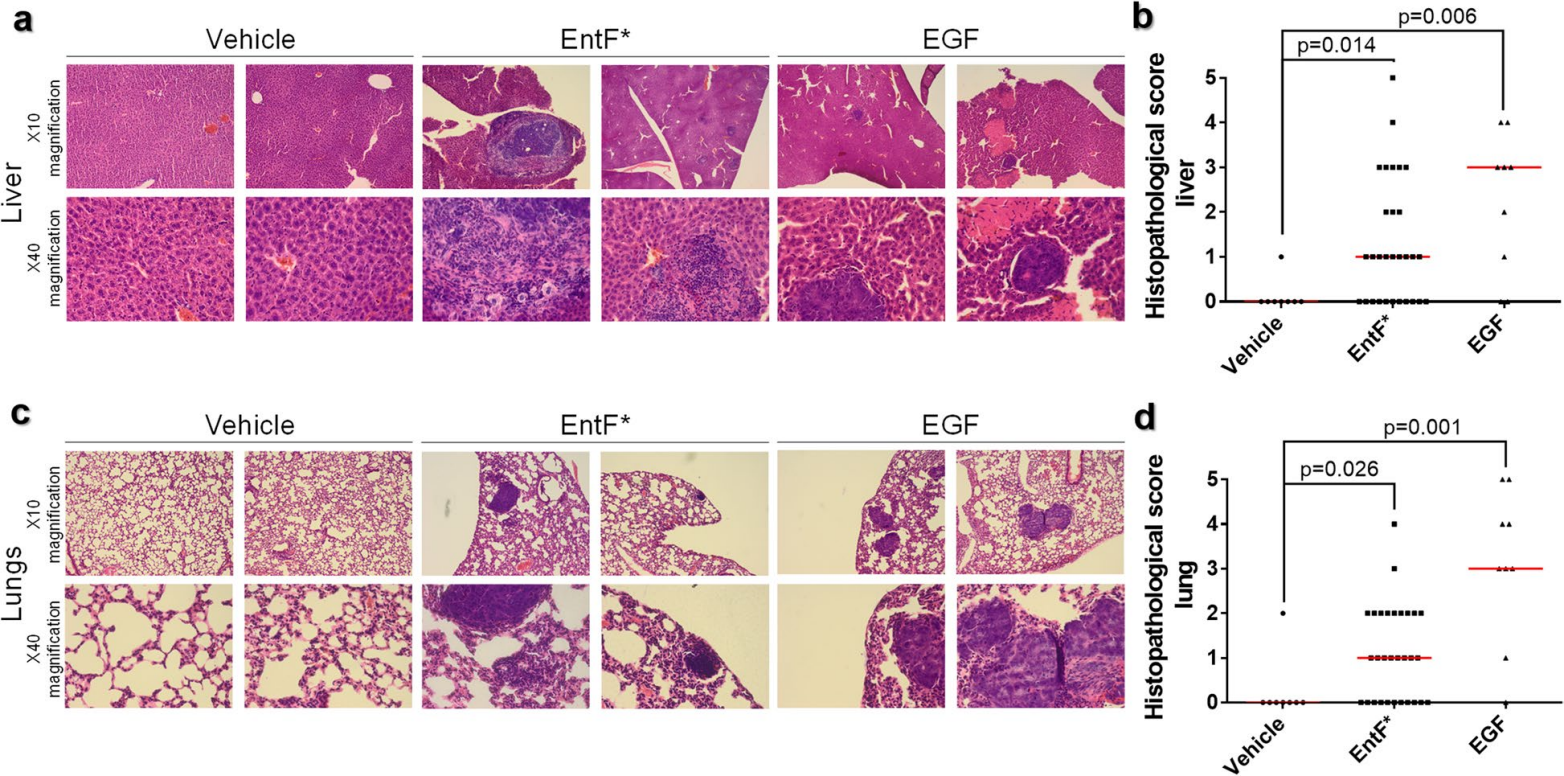
Histopathological evaluation of CRC metastasis after EntF* treatment. **a** Microscopic images of liver tissue sections from untreated (vehicle), EntF*-treated, and EGF-treated mice. Tissues were stained with Hematoxylin and Eosin (H&E) and imaged at different magnifications (10x upper row, 40x lower row). **b** Histopathological scores with statistically significant differences determined by a Mann-Whitney *U* test (*n =* 8 for PBS, *n =* 30 for EntF*, *n =* 9 for EGF) with indicated *p*-values. **c** Microscopic images of lung tissue sections from untreated (vehicle), EntF*-treated, and EGF-treated mice. Tissues were stained with H&E and imaged at different magnifications (10x upper row, 40x lower row). **d** Histopathological scores with statistically significant differences determined by a Mann-Whitney *U* test (*n =* 8 for PBS, *n =* 30 for EntF*, *n =* 9 for EGF) with indicated *p*-values

Histopathological analyses also showed a significantly higher number of tumor nodules in the liver (*p* = 0.014) and lungs (*p* = 0.026) of mice after 6 weeks of treatment with EntF*, compared to the vehicle (Fig. 5a–d; Additional file 17).

Another quorum sensing peptide, Phr0662 from *Bacillus* species, which also promoted in vitro cell invasion in our initial screening experiments [13], showed no pro-metastatic effects in the orthotopic mouse model of colorectal cancer used in this work (Additional file 5: Fig. S6). These findings indicate a certain degree of selectivity of quorum sensing peptides in metastasis promotion in vivo.

## Discussion

The in vitro metabolization analysis and the Caco-2 permeabilization assays suggest that EntF* can be present in the blood circulation of the host. This is likely to occur after the degradation of the bacterial EntF peptide in the colon or feces of the host and subsequent intestinal absorption of the resulting EntF* peptide. To unambiguously demonstrate the presence of EntF* in vivo, we developed and optimized a bioanalytical method based on RP-UPLC-TQ-MS in multiple reaction monitoring (MRM)-mode. Using this method, we detected the presence of EntF* in the serum of six mice. Also, four EntF*-positive and four EntF*-negative samples were analyzed through additional chromatographic methods: hydrophilic interaction liquid chromatography (HILIC)-UPLC-TQ-MS (LC_2_-MS_1_) as an orthogonal separation system and RP-UPLC-QTOF-MS (LC_1_-MS_2_) and RP-UPLC-QOrbitrap-MS (LC_1_-MS_3_) as high-resolution mass spectrometers. All methods confirmed the presence and identity of EntF* in the positive serum samples.

To confirm the bacterial origin of the peptide, feces from four EntF*-positive and -negative mice were tested for the presence of *E. faecium*-specific EntF DNA coding sequences. All EntF*-positive mice possessed the EntF coding sequences. In a few cases, UPLC-MS could not detect the EntF* peptide in the serum of mouse samples that tested positive for the presence of EntF DNA in the feces (e.g. sample 20181011S8). These negative samples could derive from specific *E. faecium* strains with reduced translational efficiency. These data are presented in Additional file 5: Table S3: out of three *E. faecium* strains containing the EntF gene, only one produced measurable EntF levels in vitro (LoD = 1.5 nM). Additionally, variability in peptide degradation rates (EntF to EntF*) and/or differences in gastrointestinal absorption of EntF* could further explain the weak correlation between EntF* levels in the serum and EntF DNA copy number in the feces. The presence of EntF* in the serum of germ-free mice treated with EntF-producing *E. faecium* strains represents an additional proof that the detected EntF* is indeed of microbial origin.

EntF* was previously identified by our group in an in vitro screen for molecules that selectively promote angiogenesis and tumor cell invasion in HCT-8 CRC cells [13]. In this work, we confirm these in vitro effects by proving that EntF* significantly decreases E-cadherin expression in three different colorectal cell lines. Additionally, we found that amino acids in the first (S), second (N), tenth (L) position, as well as the stereochemical configuration of the sixth residue, are important for the EMT-promoting effects. Our findings suggest that EntF* acts as an agonist-ligand in the CXCR4/EGFR phosphorylation/ERK activation/E-cadherin pathway. Indeed, when EntF* is administered together with a CXCR4 antagonist (Nef-M1), or to a CXCR4-negative colorectal cell line (HCT-116), it shows reduced or no activity.

Furthermore, we evaluated EntF* metastasis-promoting activities in vivo using an orthotopic mouse model of colorectal cancer [30, 31]. Since we used a single mouse strain and identical housing conditions, we could directly compare different treatment groups to find causal associations, as only treatments were different among groups (i.e*.*, placebo, peptide or positive control). This experiment demonstrates that the quorum sensing peptide metabolite EntF* can promote CRC metastasis in vivo. This effect seems quite specific to EntF* as Phr0662, another quorum sensing peptide, did not display metastasis-promoting effect. A significantly higher number of tumor nodules was found in the liver and lungs of EntF*-treated mice, compared to the placebo population. It is worth mentioning that the liver is the most common site of metastases from CRC: approximately half of patients with CRC will develop hepatic metastases, with a median survival time of only 8 months and a 5-year survival rate of less than 5% [24–28]. The orthotopic mouse model however has some limitations. First, nude mice, although representing a common model in cancer research, contain a genetic mutation impairing thymus function thus resulting in an inhibited immune system, which may not represent a realistic model of cancer development in humans. Secondly, tumor growth is more difficult to monitor in this system compared to a subcutaneous model. Nevertheless, we preferred the orthotopic mouse model because it better mimics the human disease process with regard to the anatomical location of the tumor and the spontaneous metastatic process [30]. We acknowledge that infecting mice with an EntF-producing *E. faecium* strain instead of using the peptide would be biologically more relevant. However, to prove the causal relationship, we needed to demonstrate the in vivo effect of the peptide without introducing additional confounding factors. Investigating the influence of *E. faecium* bacteria on CRC metastatic progression would be the next logical step.

## Conclusions

Altogether, our findings demonstrate that the quorum sensing peptide metabolite EntF* (1) is naturally present in biofluids of mice, (2) promotes CRC metastasis in an orthotopic animal model, and (3) acts as an agonist-ligand in the CXCR4/EGFR phosphorylation/ERK activation/E-cadherin pathway with a biological activity comparable to that of EGF and CXCL12, well-established human CRC growth factors. Our findings provide the first indication that quorum sensing peptide metabolites represent additional factors in host-microbiota interactions, potentially influencing CRC metastasis. Our work opens new paths for CRC research, offering new perspectives for disease prevention, diagnosis, and therapy by selective modulation of the gut microbiome.

## Methods

### Tissue homogenate preparation

Krebs-Henseleit (KH) buffer (Sigma-Aldrich, Belgium) was prepared by dissolving the powdered medium in 900 mL water while stirring. Once the powder was completely dissolved, 0.3790 g CaCl_2_•2H_2_O and 2.098 g NaHCO_3_ were added to the solution. NaOH or HCl was used to adjust the pH to 7.4. The solution was brought to final volume (1000 mL) with ultrapure water.

For the preparation of colon tissue homogenates for EntF in vitro metabolization study, colons were collected from two untreated C57BL/6 female mice after cervical dislocation. After cleaning and rinsing the organs with ice-cold KH buffer, colons were cut into small pieces and transferred into 15 mL tubes containing 5 mL ice-cold KH buffer. Colon tissues were homogenized for 1 min. Larger particles were allowed to sediment for about 30 min at 5 °C, and then approximately 2 mL of the middle layer of each tube was transferred into a 2 mL Eppendorf tube and stored at − 35 °C. Before use, the homogenate was diluted to a final protein concentration of 0.6 mg/mL.

Feces homogenates were prepared, after collecting fecal material from two untreated C57BL/6 female mice after cervical dislocation, by using the same procedures employed for colon tissue homogenate preparation. Before use, the homogenate was diluted to a final protein concentration of 0.6 mg/mL.

### Peptide adsorption

To avoid the absorption of EntF* onto plasticware and glassware, all tubes and containers were coated with a BSA-based anti-adsorption solution before use [28].

### Metabolization kinetics

Five hundred microliters of tissue homogenate were mixed with 400 μL of pre-warmed KH buffer. Then, 100 μL of KH buffer (blank) or 1 mg/mL EntF peptide solution (test) were added to the diluted homogenate and incubated at 37 °C. At 0, 5, 10, 30, 60, 120, and 180 min, 100 μL aliquots were taken and immediately mixed with 100 μL of 1% V/V trifluoroacetic acid solution in water, heated for 5 min at 95 °C, and placed in an ice bath for 30 min. After centrifugation at 16,000*g* for 30 min at 5 °C, supernatants were analyzed by LC_1_-MS_1_ for EntF* quantification.

### Cell culture

HCT-8, HT-29, and HT-116 cells were grown in DMEM medium supplied with 10% fetal bovine serum (FBS) and 1% penicillin-streptomycin. Caco-2 cells were grown in DMEM medium supplied with 10% FBS, 1% non-essential amino acid NEAA), and 1% penicillin-streptomycin. Cells were cultured at 37 °C and 5% CO_2_. As cells reached confluency, they were detached using 0.25% Trypsin-EDTA.

*E. faecium* strains (LMG 20720, LMG 23236, LMG 15710 and ATCC 8459) were grown overnight at 37 °C in BHI medium under aerobic conditions.

### Western blot analyses

For Western blot analyses, HCT-8 (*n* = 15), HCT-116 (*n* = 12), Caco-2 (*n* = 6), and HT-29 (*n* = 12) cells were seeded in 6-well plates at a density of 1 × 10^6^ cells/well. Twenty-four hours after seeding, all cell lines were treated with 1 μM EntF* or a placebo solution. HCT-8 cells were also tested for responsiveness to 1 μM EntF* synthetic alanine-derived analogs. For antagonist activity analysis, HCT-8 cells were treated with 1 μM Nef-M1 or 1 μM EntF*A1 alone or in combination with 500 nM EntF*. After 24 h, cells were detached from the surface and lysed in RIPA buffer (Thermo Scientific). Protein concentration was determined using the modified Lowry protein assay kit (Thermo Scientific), according to the manufacturer’s instructions. Protein samples were diluted to the same concentration (4 μg/μL) with water and diluted again 1:1 using 2x Laemmli buffer. After that, samples were boiled for 5 min at 95 °C to denature proteins and centrifuged for 5 min at 16,000*g*; supernatants were used for Western blot analyses. Equal amounts of total protein (20 μg/lane) were separated by SDS-PAGE on an Any kD^TM^ gel (Bio-Rad ) and then transferred to a PVDF membrane. Before incubation with antibodies, non-specific binding sites on the membranes were blocked using 5% skimmed milk solution (1 h). TBS buffer with 0.05% Tween 20 was used for all washing steps. Membranes were incubated overnight at 4 °C with an anti-E-cadherin primary antibody (1/1000 dilution). An HRP-conjugated anti-rabbit antibody (1/2000 dilution, 1 h incubation) was used for chemiluminescent detection. Membranes were incubated with the detection substrate for 5 min and signal detection was performed using the ChemiDoc EZ imager and Image Lab software (Bio-Rad). Signal intensity was normalized against the total protein content in each lanes.

A quantitative approach was used to evaluate the importance of each amino acid, where the Fisher’s LSD *p*-values of (1) multiple comparisons between EntF* and the alanine (ALA) scan and (2) multiple comparisons between different peptides of the ALA scan are combined. Subsequently, based on the combined *P*-score, the amino acids were classified in 5 classes using a hierarchical cluster analysis and confirmed using the Jenks natural breaks algorithm with *K* = 5.

$$\mathrm{Combined}\ \mathrm{Pscore}=\left(1-{P}_{PBS\ vs\ {ALA}_{scan}}\right)\ast {P}_{EntF\ast vs\ {ALA}_{scan}}$$

### Sequence alignment

To identify similar regions, EntF* was aligned to the activating or binding domains of endogenous ligands of potential targets using the Smith-Waterman algorithm. The EMBOSS Waters Smith-Waterman is a dynamic programming algorithm designed to find the best local alignment between two sequences, by determining the score of all possible alignments separately. The assigned score measures how often various amino acid replacements occur in a broad range of homologous proteins over time. This score was calculated using the BLOSUM62 substitution matrix and gap-penalty function. BLOSUM grants a score to each amino acid replacement, in regard to the observed frequencies of amino acid substitutions of related proteins [32, 33]. The Smith-Waterman score obtained for EntF* and the activating domain of protein ligands was adjusted with an additional alignment score. In this adjusted score, the contribution of the amino acids of EntF* is taken into account. The ranking of the different amino acids is inferred from the ALA scanning analysis. The individual amino acid score, from the BLOSUM62 matrix, of a match with an important amino acid of the first rank (S1, N2, L10) is multiplied by three. The score of a match with an important amino acid of the second rank (L3, S9) is multiplied by two.

### Intestinal permeability

Caco-2 cells were seeded on Transwell polycarbonate membrane filters (0.4 μm pore size, Corning, Germany) at a density of 2.6 × 10^5^ cells/cm^2^ and permeability tests performed as described by Hubatsch et al. [34]. Cells were submerged with Hank’s balanced salt solution (HBSS), and transepithelial electrical resistance (TEER) was measured before and after the experiment. Peptide solution (1 μM) was added to the apical chamber, and 300 μL aliquots were taken after 30, 60, 90, and 120 min of incubation. Samples were analyzed using LC_1_-MS_1_. Linear curve fitting was used to calculate the apparent permeability coefficient (P_app_).

### Sample collection and preservation

Untreated C57BL/6 mice, possessing their natural microbiome, were euthanized by cervical dislocation and their blood was collected. After a 30 min incubation on ice, blood was centrifuged at 1000*g* for 10 min at room temperature. The supernatant (serum) was then transferred to fresh tubes and stored at − 35 °C until use.

After defecation, two droppings of feces were immediately collected and put in liquid nitrogen for maximum 1 h. Samples were then stored at − 80 °C until use.

### Sample preparation

Fifty microliters of serum were mixed with 150 μL 0.5% formic acid in acetonitrile. After sonication for 5 min, samples were vortexed for 5 s, then heated for 30 s at 100 °C. The solution was vortexed and centrifuged for 20 min at 20,000*g* at 4 °C. The supernatant was purified using solid phase extraction (SPE) on HyperSep C_18_ plates (Thermo Fisher Scientific, Belgium), previously conditioned with acetonitrile and equilibrated with 75% acetonitrile in water, containing 0.375% formic acid. After loading 150 μL of each sample, 120 μL eluent were collected and organic solvents evaporated using nitrogen (1 L/min) for 5 min. The resulting samples were further diluted with 30 μL of BSA-based anti-adsorption solution, and analyzed by LC-MS.

For EntF and EntF* colon content analysis, 50 mg of colon content were suspended in 100 μL of a 2% hydrochloric acid solution, vortexed and sonicated for 30 s. The suspension was centrifuged for 1 min at 3000*g*, and 50 μL of the supernatant was heated at 100 °C for 1 min and cooled on ice for 1 min. Samples were centrifuged again for 1 min at 10,000*g*, and 30 μL of supernatant was added to a 900 μL of a 3% DMSO solution in acetonitrile acidified with 0.1% formic acid (equilibration solution). Nine hundred microliters of the diluted samples was loaded on a HILIC amide SPE MonoSpin column previously conditioned with acetonitrile and equilibrated with the equilibration solution. Samples were eluted using a mixture of 75/20/5 (V/V/V) H_2_O/acetonitrile/DMSO acidified with 0.1% formic acid.

Bacterial culture medium was centrifuged for 10 min at 2095 g at 4 °C, and the supernatant filtered through a 0.20-μm filter. For culture medium purification, 200 μL of broth were loaded on an Oasis HLB μElution plate (Waters, Belgium), previously conditioned with acetonitrile and equilibrated with water. EntF was eluted from the column using 200 μL of 70% methanol in water containing 2% of formic acid, and organic solvents evaporated using nitrogen (1 L/min) for 4 min. The resulting solution was then further diluted with 150 μL of acetonitrile containing 2% of formic acid and analyzed by LC_2_-MS_1_.

### RP-UPLC-TQ-MS (LC_1_-MS_1_) analysis

EntF* was detected and quantified on a Waters Acquity® UPLC H-class system, connected to a Waters Xevo™ TQ-S triple quadrupole mass spectrometer with electrospray ionization (operated in positive ionization mode). Autosampler tray and column oven were kept at 10 ± 5 °C and 60 ± 5 °C, respectively. Chromatographic separation was achieved on a Waters Acquity® UPLC BEH Peptide C_18_ column (300 Å, 1.7 μm, 2.1 mm × 100 mm). The mobile phases consisted of 93/2/5 water/acetonitrile/DMSO (V/V/V) containing 0.1% formic acid (mobile phase A) and 2/93/5 water/acetonitrile/DMSO (V/V/V) containing 0.1% formic acid (mobile phase B), and the flow rate was set to 0.5 mL/min. A 10-μL aliquot from each sample was injected. The gradient program started with 80% of mobile phase A for 1 min, followed by a linear gradient to 40% of mobile phase A for 3.5 min. Gradient was then changed to 14.2% mobile phase A at 5 min, followed by a 1 min equilibration, before starting conditions were applied. EntF* retention time was between 4.25 and 4.45 min.

An optimized capillary voltage of 3.00 kV, a cone voltage of 20.00 V, and a source offset of 50.0 V were used. Acquisition was done in the MRM mode. The selected precursor ion for EntF* was *m/z 865.7* with two selected product ions at *m/z 202.08* (36 eV, b_2_ fragment) as quantifier and *m/z 315.17* (31 eV, b_3_ fragment) as qualifier.

A sample was considered positive for the presence of EntF* when the following criteria were met: correct retention time, quantifier/qualifier peak area ratio between 2.0 and 4.0, both quantifier and qualifier peaks with a signal-to-noise ratio > 3.0, and a peptide concentration > 100 pM.

### RP-UPLC-QTOF-MS (LC_1_-MS_2_) analysis

Chromatographic separation was achieved on a Waters Acquity® UPLC HSS T3 Column (100 Å, 1.8 μm, 2.1 mm × 100 mm), and products were detected using the Waters SYNAPT G2-Si High Definition Mass Spectrometry with electrospray ionization (operated in the positive ionization mode). Gradient composition and UPLC-MS settings were the same as with the LC_1_-MS_1_ method; a TOF-MS/MS mode was applied with a fixed mass on the quadrupole of *m/z 865.157*, a fixed trap collision energy of 30 eV, and an acquired MS/MS over the range of 100–1450 m/z (scan time 1 s). EntF* retention time was between 3.40-3.50 min. When at least four EntF* daughter ions (m/z ± 0.05) were detected at the expected retention time and at least three of the most abundant isotope parent peaks (m/z ± 0.05) were detected, the sample was considered EntF* positive.

### RP-UPLC-Q-Orbitrap-MS (LC_1_-MS_3_) analysis

While the UPLC separation system was the same as the LC_1_-MS_1_ method, the third detection system consisted of a Thermo Fisher Q Exactive™ Hybrid Quadrupole-Orbitrap. The mass spectrometer was operated using a heated electrospray ionization source with the following settings: capillary temperature set at 300 °C, S-Lens RF level set at 50, spray voltage set at 3.00 kV, and auxiliary gas flow set at 20.

A full MS/MS mode was applied with a fixed mass on the quadrupole of *m/z 865.157*, a fixed trap collision energy of 30 eV and 35 eV and an acquired MS/MS over the range of 100-1800 m/z. EntF* retention time was 4.13-4.16 min. When at least two daughter ions (m/z ± 0.005) of EntF* were detected at the expected retention time and at least four of the most abundant isotope parent peaks (m/z ± 0.005) were detected, the sample was considered positive for the presence of EntF*.

### HILIC-UPLC-TQ-MS (LC_2_-MS_1_) analysis

Chromatographic separation was achieved on a Waters Acquity® UPLC BEH Amide Column (130 Å, 1.7 μm, 2.1 mm × 100 mm). Mobile phase composition, sample volume, flow rate, and MS settings were as described for the LC_1_-MS_1_ method. For EntF* quantification in mouse sera, the gradient program started with 10% of mobile phase A for 2 min, followed by a linear gradient to 40% of mobile phase A for 3.0 min. Gradient was then changed to 85% mobile phase A at 6 min, followed by a 1 min equilibration, before starting conditions were applied. EntF* retention time was 4.85–4.95 min. A sample was considered positive for the presence of EntF* when following criteria were met: correct retention time, both daughter fragments [b_2_ (quantifier) and b_3_ (qualifier) fragment ions] with a signal-to-noise ratio > 3.0, and quantifier/qualifier peak area ratio between 2.0 and 4.0.

For the quantification of EntF in the culture medium, the gradient program started with 100% of mobile phase B for 2 min, followed by a linear gradient to 40% of mobile phase B for 7 min, cleaning at 85% B and re-equilibration at starting conditions. Acquisition was done in the MRM mode. The selected precursor ion for EntF was *m/z 667.1* with three selected product ions: *m/z 129.0* (30 eV, b_2_ fragment) and *m/z 662.6* (22 eV, b_25_ fragment), both as qualifier, and *m/z 949.4* (22 eV, y_17_ fragment) as quantifier.

### DNA extraction from feces

Twenty to 40 mg of fecal material were mixed with 500 mg of unwashed glass beads, 0.5 mL CTAB buffer (hexadecyltrimethylammonium bromide 5% w/v, 0.35 M NaCl, 120 mM K_2_HPO_4_) and 0.5 mL phenol/chloroform/isoamyl alcohol (25/24/1). The mixture was twice homogenized for 1.5 min at 22.5 Hz using a TissueLyser II (Qiagen, Belgium). The mixture was then centrifuged for 10 min at 8000 rpm, and 300 μL of the resulting supernatant was transferred to a new Eppendorf tube. The original feces/bead sample was again mixed with 0.25 mL CTAB buffer, homogenized, and centrifuged for 10 min at 8000 rpm. Three hundred μL of the supernatant was added to the supernatant collected in the previous step. Phenol was removed by adding an equal volume of chloroform/isoamyl alcohol (24/1) followed by centrifugation at 16,000*g* for 10 s. The aqueous phase was transferred to a new tube. Nucleic acids were precipitated with 2 volumes of a PEG-6000 solution (polyethylene glycol 30% w/v, 1.6 M NaCl) for 2 h at room temperature. The sample was centrifuged at 13,000*g* for 20 min and the resulting DNA pellet was washed with 1 mL of ice-cold 70% (v/v) ethanol. After centrifugation at 13,000*g* for 20 min, the pellet was dried and resuspended in 50 μL de-ionized water. The quality and concentration of the DNA were determined spectrophotometrically.

### Real time quantitative PCR (qPCR) on DNA from fecal samples

qPCR was performed using the Bio-Rad CFX-384 Real Time PCR system. Amplification was carried out in 12 μL (final reaction volume) containing 2x SYBR-green master mix, 2 μL DNA sample, and 0.5 μM of each primer. Each reaction was run in six replicates. Amplification was performed as follows: 95 °C for 10 min, 40 cycles at 95 °C for 30 s, 60 °C for 30 s, and stepwise increase of the temperature from 65° to 95 °C (at 10 s/0.5 °C). Melting curve data were analyzed to confirm the specificity of the reaction. Samples with nonspecific melting peaks were excluded from further analyses. DNA copy number was determined by comparing sample Ct values to the standard curve. The DNA product used to generate the standard curve, was amplified from *E. faecium* strain 100-1 with the EntF-specific PCR primers listed in Additional file 5: Fig. S2. After PCR product purification (MSB Spin PCRapace, Stratec Molecular, Berlin, Germany) and determination of the DNA concentration, a 10-fold serial dilution ranging from 1 × 10^7^ to 1 × 10^1^ DNA copies per μL was created and used to generate the standard curve. Since the Cq values of the PCR product were close to the LOD, with a significant amount of left-truncated data (data below LOD), a maximum likelihood (ML) approach was used to find the best estimation of mean and standard deviation for each sample.

### EntF production in germ-free mice

Mice were obtained from the Ghent Germfree and Gnotobiotic mouse facility (LA1400637), maintained in a sterile environment under controlled conditions (light–dark cycle with light from 7:00 h to 17:00 h, temperature 21–25 °C, and humidity 45–65%). Twelve germ-free female C57BL6 mice were divided into 3 groups and each group (*n* = 4) was kept in a separate cage. At day 0, the placebo group was treated with the cell medium (BHI), the control group received a mixture of 3 EntF-negative *E. faecium* strains (NCIMB 10415; W54 and LMG S-28935 each 10^8^ CFU per strain) suspended in 300 μL of BHI, while the test group received a mixture of 3 EntF-producing *E. faecium* strains (LMG 23236; T-110 and ATCC 8459 each 10^8^ CFU/strain) suspended in 300 μL of BHI. After 4 days, feces were collected, mice were sacrificed, and the blood and whole colon, including the content, were obtained. Blood samples were immediately transferred to 1.5 ml Eppendorf tubes, put on ice for 10 to 30 min and centrifuged at 1000*g* for 10 min. Serum samples were stored at − 80 °C and subsequently analyzed for the presence of EntF* using LC_1_-MS_1_. Colon contents were immediately transferred to 15/50 ml tubes, put on dry ice for 1 to 3 h and stored at − 80 °C. Samples were further analyzed for the presence of EntF* using LC_1_-MS_1_.

### Standard protein BLAST

The amino acid sequence of the EntF* peptide was blasted against the NCBI non-redundant (nr) database by Basic Local Alignment Search Tool protein (BLASTp). The blast search was limited to bacterial sequences (taxid:2). Only alignment hits with a 100% coverage and 100% identity were considered.

### Orthotopic mouse model of colorectal cancer

All in vivo experiments were performed according to the Ethical Committee principles of laboratory animal welfare, approved by our institute (Ghent University, Faculty of Medicine and Health Sciences, approval number ECD 17-90). Mice were maintained in a sterile environment under controlled conditions (light–dark cycle with light from 7:00 h to 17:00 h, temperature 21–25 °C and humidity 45–65%). Before the experiment, mice were allowed to acclimate to the abovementioned conditions for a minimum of 7 days.

Six-week-old female athymic nude mice (Swiss nu/nu) were anesthetized, and a minimally invasive midline laparotomy was performed to localize the cecum. The cecum was then gently pulled out, and luciferase-transfected HCT-8/E11 cells (1 × 10^6^ cells) in 20 μL serum-free DMEM medium with Matrigel (1:1) were injected into the cecal wall. Before implantation, cells were treated with EntF* (10^2^ nM), Phr0662 (10^2^ nM), vehicle (PBS), or TGFα positive control (0.1 μg mL^−1^) solutions for 5 days. The cecum was then carefully placed back into the abdominal cavity and the surgical incision was closed with two layers of sutures with PDS 6-0. The day after injection, mice were treated daily with vehicle (PBS; *n* = 15), EntF* (10^2^ nmol kg^−1^; *n* = 38), Phr0662 (10^2^ nmol kg^−1^; *n* = 5), or EGF positive control (100 μg kg^−1^; *n* = 18) for 6 weeks. Once a week, mice were examined for tumor growth and metastases using bioluminescent imaging with the IVIS Lumina II (Perkin Elmer, Belgium) after i.p. injection with 200 μL luciferin (150 mg kg^−1^). After 6 weeks, mice were euthanized using cervical dislocation, and the liver, diaphragm, lungs, cecum, duodenum, and peritoneum were macroscopically examined for the presence of tumor nodules. Liver and lung tissues were then fixed in formalin for 24 h and stored in 70% ethanol for maximum 3 days before embedding in paraffin. Next, a hematoxylin and eosin (H&E) staining was performed on 8 μm sections, and 3 sections per mouse were visualized using microscopy. Slides from tumor-bearing mice were analyzed by two blinded, independent investigators using the scoring system described in Additional file 5: Table S3. In case of differences, slides were scored by a third blinded, independent investigator for consensus.

To calculate daily peptide exposure, female Swiss nu/nu mice (*n* = 14) were i.p. injected with 25 μL of 100 μM EntF*, their serum withdrawn at different time points and analyzed by LC_1_-MS_1_. A distribution and early exponential phase (*α*, 0-30 min), followed by a terminal elimination phase (*β*, 30–180 min) could be distinguished. The exposure was determined over a period of 24 h (x) as follows: $Exposure\ \left( nM\ x\ \min \right)=\int_0^{30}A\ {e}^{-\alpha t}+\int_{30}^xB\ {e}^{-\beta t}$.

### PCR on E. faecium strains

*E. faecium* was grown in BHI medium, bacterial DNA was extracted using alkaline lysis, and the EntF-encoding DNA fragment was amplified in a Mastercycler PCR system (Eppendorf, Belgium). Each reaction was prepared in 10 μL (final reaction volume) containing 2x BioMix (Bioline, Belgium) and 1 μL of DNA 0.5 μM of each primer (EntF*-PCR primers, Additional file 5: Fig. S2b). Amplification was performed as follows: 1 cycle of 94 °C for 5 min, followed by 30 cycles of 94 °C for 30 s, 55 °C for 30 s, and 72 °C for 1 min. Final elongation was performed at 72 °C for 10 min, after which PCR products were kept at 4 °C. The PCR amplification products were resolved on 1.5% agarose gel.

### Statistical analyses

The Kolmogorov-Smirnov test was used to assess if data obtained were normally distributed. For sample size *n* < 10, non-parametric tests (Mann-Whitney *U* test) were performed directly. Slope comparison was based on linear regression analysis. Bootstrapped medians and Hedges *G*-values were used to calculate the effect size when sample sizes were different between the groups. Cohen’s *d* values were calculated as a measure of the effect size when similar standard deviations for both groups were found, and sample sizes were the same.

## Supplementary Information

**Additional file 1.** Raw data Excel files.

**Additional file 2.** Raw data files formation EntF*.

**Additional file 3.** Raw data files Caco-2 experiments.

**Additional file 4.** Raw data LC_1_-MS_1_ files mice samples.

**Additional file 5: Figure S1.** Verification of the LC_1_-MS_1_ method. **Figure S2.** qPCR detection of EntF. **Figure S3.** Effect of EntF* and EntF* analogues on E-cadherin expression. **Figure S4.** Representative images of Western blot analyses. **Figure S5.** Overview of E-cadherin-regulating pathways. **Figure S6.** In vivo effects of the Phr0662 quorum sensing peptide in an orthotopic mouse model of colorectal cancer. **Table S1.** Concentration of EntF* in mice serum measured using the LC_1_-MS_1_ method and confirmation of the results using additional chromatographic method and qPCR. **Table S2.** Synopsis of known receptors involved in E-cadherin downregulation, their natural ligands, natural ligands’ active domains and alignment scores with EntF* peptide. **Table S3.** Presence of EntF gene and peptide in different *E. faecium* strains. **Table S4.** Histopathological scoring system.

**Additional file 6.** Raw data LC_1_-MS_1_ verification files.

**Additional file 7.** Raw data LC_1_-MS_3_ files mice samples.

**Additional file 8.** Raw data LC_1_-MS_2_ files mice samples.

**Additional file 9.** Raw data LC_2_-MS_1_ files mice samples.

**Additional file 10.** Raw data files gnotobiotic mice experiment.

**Additional file 11.** Raw data files Western Blot Alanine analogues.

**Additional file 12.** Raw data files Western Blot D-amino acid analogues.

**Additional file 13.** Raw data files Western Blot antagonist study.

**Additional file 14.** Raw data files Western Blot cell lines.

**Additional file 15.** Raw data files exposure study.

**Additional file 16.** Raw data bioluminescence files.

**Additional file 17.** Raw data microscopy files.

## Data Availability

All data generated or analyzed during this study are included in this article or its supplementary information files. Mass spectrometry data are also publicly available through the MassIVE repository, accession numbers MSV000088315 [35] and MSV000088317 [36].

## Abbreviations

CRC: Colorectal cancer
mCRC: Colorectal cancer metastasis
EMT: Epithelial-to-mesenchymal transition
CAC: Colitis-associated colon cancer
IBD: Inflammatory bowel disease
SEM: Standard error of the mean
RP-UPLC-TQ-MS: Reversed phase-ultra-high performance liquid chromatography–tandem quadrupole-mass spectrometry
LOQ: Limit of quantification
LoD: Limit of detection
CXCR4: C-X-C chemokine receptor type 4
IL-6: Interleukin 6
VEGF: Vascular endothelial growth factor
CXCL12: C-X-C motif chemokine 12
PBS: Phosphate-buffered saline
TGFα: Transforming growth factor α
EGF: Epidermal growth factor
i.p.: Intraperitoneal
MRM: Multiple reaction monitoring
BSA: Bovine serum albumin
HILIC-UPLC-TQ-MS: Hydrophilic interaction liquid chromatography–ultra-high performance liquid chromatography–tandem quadrupole-mass spectrometry
RP-UPLC-QTOF-MS: Reversed phase–ultra-high performance liquid chromatography–quadrupole time of flight–mass spectrometry
RP-UPLC-QOrbitrap-MS: Reversed phase-ultra-high performance liquid chromatography–QOrbitrap–mass spectrometry
KH: Krebs-Henseleit
FBS: Fetal bovine serum
ALA-scan: Alanine-scan
HBSS: Hank’s balanced salt solution
TEER: Transepithelial electrical resistance
H&E: Hematoxylin and eosin
