# Supplementary material for: The quorum sensing peptide EntF* promotes colorectal cancer metastasis in mice: a new factor in the host-microbiome interaction
Source: BMC Biol. 2022 Jun 27;20:151. doi: 10.1186/s12915-022-01317-z (PMC9238271; doi:10.1186/s12915-022-01317-z)
Supplement: Supplementary file 5 — Additional file 5: Figure S1. Verification of the LC1-MS1 method. Figure S2. qPCR detection of EntF. Figure S3. Effect of EntF* and EntF* analogues on E-cadherin expression. Figure S4. Representative images of Western blot analyses. Figure S5. Overview of E-cadherin-regulating pathways. Figure S6. In vivo effects of the Phr0662 quorum sensing peptide in an orthotopic mouse model of colorectal cancer. Table S1. Concentration of EntF* in mice serum measured using the LC1-MS1 method and confirmation of the results using additional chromatographic method and qPCR. Table S2. Synopsis of known receptors involved in E-cadherin downregulation, their natural ligands, natural ligands’ active domains and alignment scores with EntF* peptide. Table S3. Presence of EntF gene and peptide in different E. faecium strains. Table S4. Histopathological scoring system. [file 12915_2022_1317_MOESM5_ESM.docx]

**SUPPLEMENTARY INFORMATION**

**THE QUORUM SENSING PEPTIDE ENTF* PROMOTES COLORECTAL CANCER METASTASIS IN MICE: A NEW FACTOR IN THE MICROBIOME-HOST INTERACTION**

Evelien Wynendaele^1,*^, Nathan Debunne^1,*^, Yorick Janssens^1^, Anton De Spiegeleer^1,2^, Frederick Verbeke^1^, Liesa Tack^1^, Sophie Van Welden^2^, Evy Goossens^3^, Daniel Knappe^4^, Ralf Hoffmann^4^, Christophe Van De Wiele^5^, Debby Laukens^2^, Peter Van Eenoo^5^, Filip Van Immerseel^3^, Olivier De Wever^6^ & Bart De Spiegeleer^1^

^1^ Drug Quality and Registration Group, Faculty of Pharmaceutical Sciences, Ghent University, Ghent, Belgium.

^2^ [Department of Internal Medicine and Pediatrics](https://telefoonboek.ugent.be/en/faculties/ge35), Faculty of Medicine and Health Sciences, Ghent University, Ghent, Belgium.

^3^ [Department of Pathology, Bacteriology and Poultry diseases](https://telefoonboek.ugent.be/en/faculties/di05), Faculty of Veterinary Medicine, Ghent University, Ghent, Belgium.

^4^ Center of Biotechnology and Biomedicine, Faculty of Chemistry and Mineralogy, Universität Leipzig, Leipzig, Germany.

^5^ [Department of Diagnostic](https://telefoonboek.ugent.be/en/faculties/ge35) Sciences, Faculty of Medicine and Health Sciences, Ghent University, Ghent, Belgium.

^6^ [Department of Human](https://telefoonboek.ugent.be/en/faculties/ge35) Structure and Repair, Faculty of Medicine and Health Sciences, Ghent University, Ghent, Belgium.

* These authors contributed equally to this work.

Corresponding author e-mail: Bart.DeSpiegeleer@UGent.be

**Supplementary Figures**

**Fig. S1. Verification of the LC_1_-MS_1_ method.** Calibration curve was created using 7 measurements from independent preparations of one serum sample, spiked at 3 different concentrations: 100 pM (*n=*4), 250 pM (*n=*1) and 1 nM (*n=*2). The best-fitted regression line represents the calibration curve with indicated R²-value. The accuracy (± 17.1%) and the precision (RSD = ± 10.3%) were determined out of the QC samples measured at 500 pM (*n=*3, Red). A precision of 31.7% was measured at the limit of quantification. The shaded bar represents the area measured in negative samples.

**Fig. S2. qPCR detection of EntF. a,** Representative standard curve for EntF primers. **b,** DNA was extracted from 20-40 mg of fecal material and six technical qPCR replicates were run using the indicated inner primer pair (red). The standard curve was generated by amplifying the DNA sequence delimited by the outer primer pair (blue).


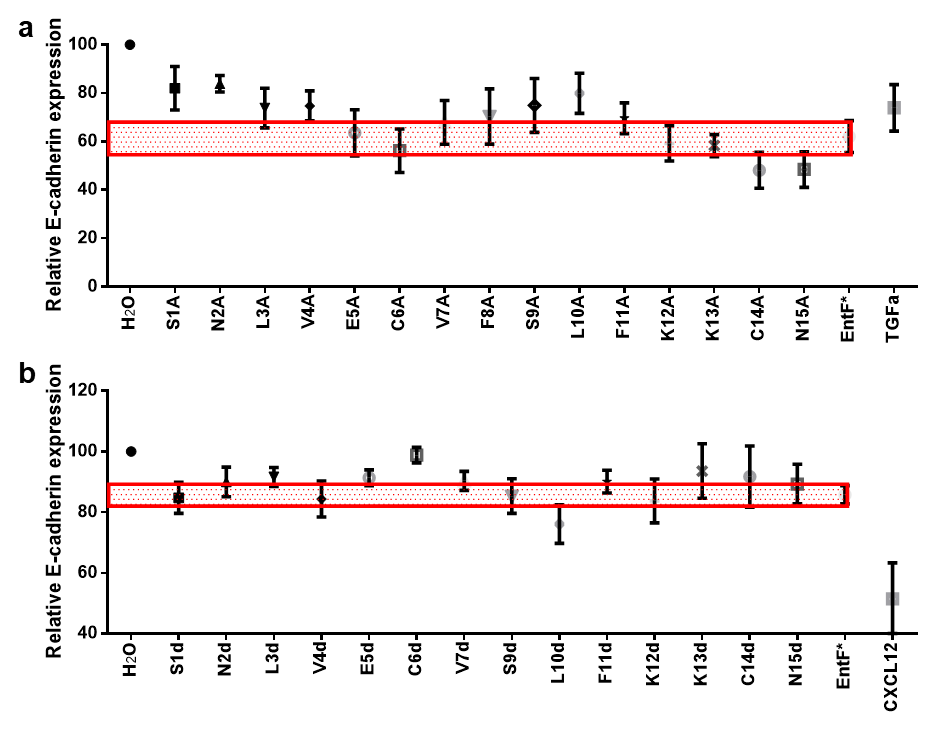


**Fig. S3. Effect of EntF* and EntF* analogues on E-cadherin expression. a**, Relative E-cadherin expression levels in response to ALA-scanning peptides and EntF* treatments (red area) in HCT-8 cells compared to negative (H_2_O) and positive (TGFα) controls (mean ± SEM; n=12), obtained by densitometric image quantification of Western blots (see Figure S5). **b,** Relative E-cadherin levels in response to EntF* (red area) and EntF* D-amino acid isomers treatments in HCT-8 cells, compared to negative (H_2_O) and positive (CXCL12) controls (mean ± SEM; n=11), obtained by densitometric image quantification of Western blots (see Figure S5). Significant different E-cadherin levels were observed between EntF* and EntF*d6, where the sixth amino acid of EntF* was replaced by its D-amino acid isomer.

**
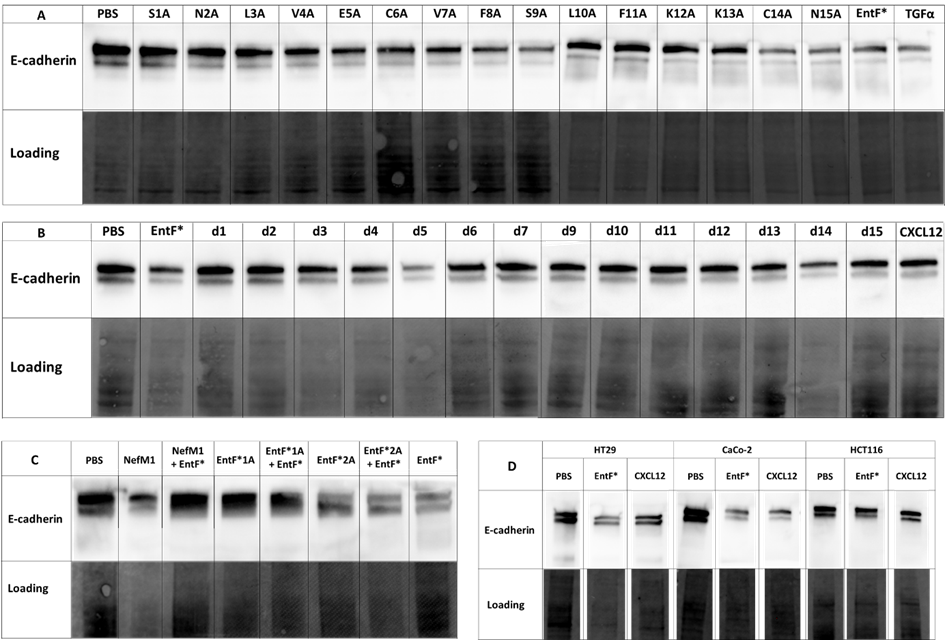
**

**Fig. S4. Representative images of Western blot analyses. a,** E-cadherin levels in untreated (PBS), EntF*-derived ALA-scanning peptide-treated (S1A to N15A), and EntF*-treated HCT-8 cells. TGFα treatments were used as positive control. **b,** E-cadherin levels in untreated (PBS), EntF*-derived D-scan peptide-treated (d1 to d15), and EntF*-treated HCT-8 cells. CXCL12-treatments were used as positive controls. **c,** E-cadherin levels in HCT-8 cells treated with EntF* alone, antagonist molecules alone (NefM1, EntF*1A, and EntF*2A), or together with EntF* (NefM1+ EntF*; EntF*1A+EntF*; EntF*2A+EntF*). PBS represents the untreated control. **d,** E-cadherin levels in untreated, EntF*-treated and CXCL12-treated HT29, Caco-2 and HCT116 cell lines. Loading control images were obtained using a stain-free gel technology (Bio-Rad) allowing for total protein visualization and quantification**.**


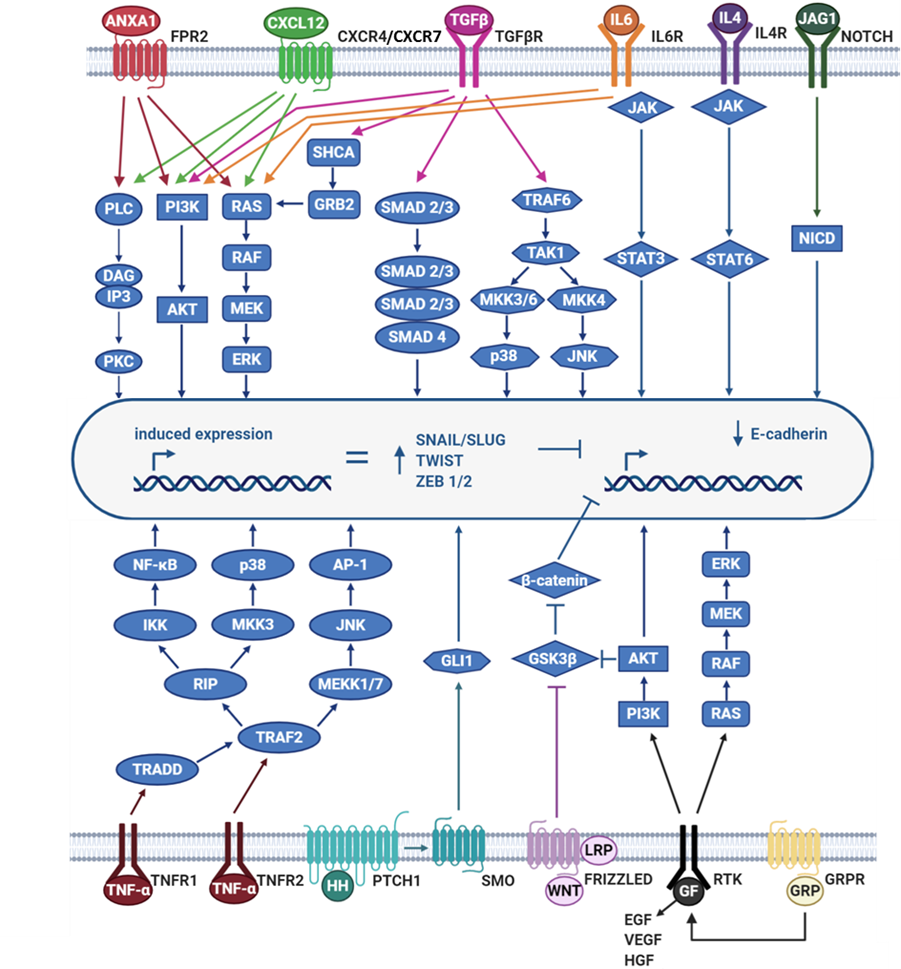


**Fig. S5.**  **Overview of E-cadherin-regulating pathways.** Schematic representation of receptors and signaling pathways leading to the activation of EMT transcription factors, followed by the downregulation of E-cadherin expression.

**Fig. S6. *In vivo* effects of the Phr0662 quorum sensing peptide in an orthotopic mouse model of colorectal cancer.** Representative images of bioluminescence activity in mice treated with vehicle (n = 7), 100 nmol kg^-1^ Phr0662 (n = 5), or 100 µg kg^-1^ EGF (n = 8). Mice were injected with 150 mg kg^‑1^ luciferin and imaged after 10 minutes in supine position. No significant differences in tumor progression were observed between Phr0662 and placebo treatments.

# **Supplementary Tables**

| **Sample** | **ID** | **Detection** | **Confirmation** | | | |
| --- | --- | --- | --- | --- | --- | --- |
|  |  | **EntF* concentration (pM) LC_1_-MS_1_** | **LC_1_-MS_2_** | **LC_1_-MS_3_** | **LC_2_-MS_1_** | **qPCR**  **(DNA copy n/g feces)** |
| 1 | 20180827S1 | <LOQ |  |  |  |  |
| 2 | 20180827S2 | <LOQ |  |  |  |  |
| 3 | 20180827S3 | <LOQ |  |  |  |  |
| 4 | 20180827S5 | <LOQ |  |  |  |  |
| 5 | 20180827S6 | <LOQ |  |  |  |  |
| 6 | 20180827S7 | <LOQ |  |  |  |  |
| 7 | 20180827S8 | <LOQ |  |  |  |  |
| 8 | 20180827S9 | <LOQ |  |  |  |  |
| 9 | 20180827S10 | <LOQ |  |  |  |  |
| 10 | 20180829S2 | <LOQ |  |  |  |  |
| 11 | 20180829S3 | <LOQ |  |  |  |  |
| 12 | 20180829S4 | <LOQ |  |  |  |  |
| 13 | 20180829S5 | <LOQ |  |  |  |  |
| 14 | 20180829S6 | <LOQ |  |  |  |  |
| 15 | 20180829S7 | <LOQ |  |  |  |  |
| 16 | 20180829S8 | <LOQ |  |  |  |  |
| 17 | 20180829S9 | <LOQ |  |  |  |  |
| 18 | 20181011S1 | <LOQ |  |  |  |  |
| 19 | 20181011S2 | <LOQ |  |  |  |  |
| 20 | 20181011S3 | <LOQ |  |  |  |  |
| **21** | **20181011S4** | **389** |  |  |  |  |
| 22 | 20181011S5 | <LOQ |  |  |  |  |
| 23 | 20181011S6 | <LOQ |  |  |  |  |
| **24** | **20181011S7** | **2146** | **+** | **+** | **+** | 588 |
| 25 | 20181011S8 | <LOQ | - | - | - | 612 |
| **26** | **20181011S9** | **3127** | **+** | **+** | **+** | 3060 |
| 27 | 20181011S10 | <LOQ | - | - | - | < LOQ |
| 28 | 20181011S11 | <LOQ | - | - | - | 500 |
| **29** | **20181011S12** | **2489** | **+** | **+** | **+** | 1079 |
| 30 | 20181011S13 | <LOQ | - | - | - | 2300 |
| 31 | 20181011S14 | <LOQ |  |  |  |  |
| 32 | 20181011S15 | <LOQ |  |  |  |  |
| **33** | **20181011S16** | **3148** | **+** | **+** | **+** | 1858 |
| 34 | 20181011S17 | <LOQ |  |  |  |  |
| **35** | **20181011S18** | **200** |  |  |  |  |

**Table S1. Concentration of EntF* in mice serum measured using the LC_1_-MS_1_ method and confirmation of the results using additional chromatographic method and qPCR.** Out of 35 serum samples, 6 tested positive for the presence of EntF* (indicated in bold), using the LC_1_-MS_1_ method. Four positive and four negative samples (indicated in red) were used for further analyses. The concentration of EntF* in mouse serum was 329 ± 150 pM when all samples (n=35) were included in the analysis. In this case, samples with EntF* concentration < LOQ were counted as zero. If only the six positive samples are considered, then EntF* concentration is 1.91 ± 0.54 nM (mean ± SEM: n=6). The presence of EntF* in the 6 samples was confirmed using 3 different chromatographic methods. Using qPCR analysis, DNA sequences encoding EntF were found in all LC-MS positive samples (20181011S7, 20181011S9, 20181011S12, 20181011S16); no DNA sequences encoding EntF could be detected in sample 20181011S10. +: present; -: not present; grey: not investigated.

| **Receptor** | **Natural ligand** | **Active domain of natural ligands** | **Alignment score with EntF*** |
| --- | --- | --- | --- |
| CXCR4 | CXCL12 | K_22_ – C_30_ | 27 |
| IL-6R | IL-6 | S_204_ – M_212_ | 21 |
| VEGFR | VEGF | D_89_ – T_103_ | 19 |
| HGFR | HGF | Q_32_ – C_206_ | 16 |
| Frizzled | Wnt | C_347_ – C_357_ | 15 |
| Notch | Jagged-1 | V_185_ – D_229_ | 15 |
| FPR2 | Annexin A1 | A_2_ – S_26_ | 14 |
| Patched | Hedgehog | C_24_ – A_197_ | 13 |
| EGFR | EGF | C_976_ – C_1001_ | 13 |
| TβR | TGF-β | Y_299_ – A_319_ | 13 |
| IL-4R | IL-4 | I_29_ – W_115_ | 12 |
| GRPR | GRP | n.a. | 9 |
| TNFR | TNF-α | W_108_ – N_110_ | 8 |

**Table S2. Synopsis of known receptors involved in E-cadherin downregulation, their natural ligands, natural ligands’ active domains and alignment scores with EntF* peptide.**

| **Bacterial strain** | **Origin** | **Presence of EntF gene** | **Presence of EntF peptide** |
| --- | --- | --- | --- |
| *E. faecium* LMG 20720 | Human feces | Yes | < LOD |
| *E. faecium* LMG 23236 | Human feces (healthy) | Yes | < LOD |
| *E. faecium* LMG 15710 | Human feces (diarrhea) | No | < LOD |
| *E. faecium* ATCC 8459 | Dairy product (cheese) | Yes | 477 nM |

**Table S3. Presence of EntF gene and peptide in different *E. faecium* strains.** Four different, commercially available strains of *E. faecium* were examined for the presence of the EntF gene using PCR. EntF gene was detected in 3 strains, while it was absent in the LMG 15710 strain. The presence of the EntF peptide in the culture media was also examined: among the PCR-positive strains, only ATCC 8459 produced EntF *in vitro* (LOD = 1.5 nM).

| **Liver scoring system** | | | |
| --- | --- | --- | --- |
| **Score** | **Parameters** | **10x Magnification** | **40x Magnification** |
| 0 | - Well-structured cells - Absence of nodular infiltrates - Absence of necrotic tissue | 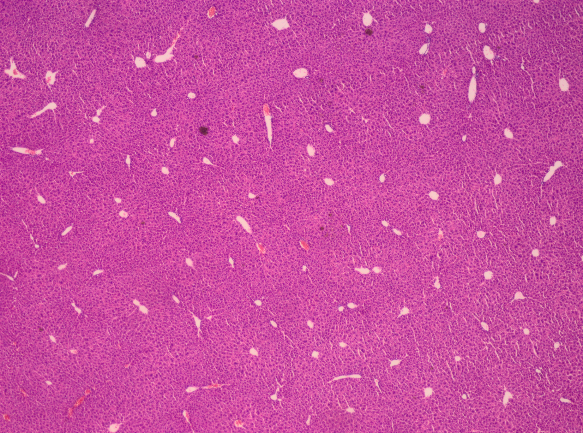 | 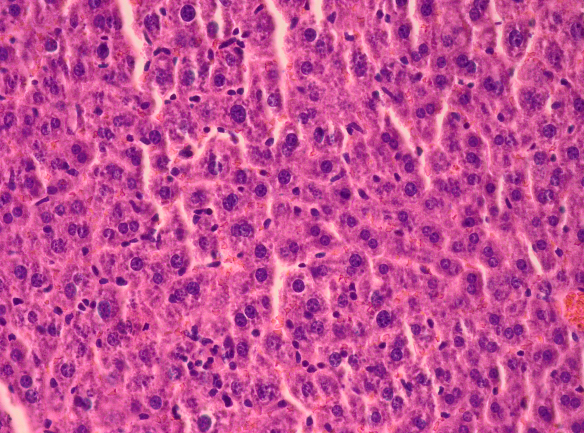 |
| 1 | - Nodular infiltrates - No capsular organization - Absence of necrotic tissue | 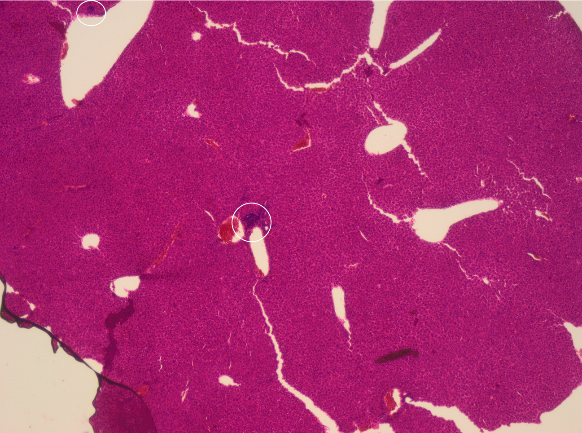 | 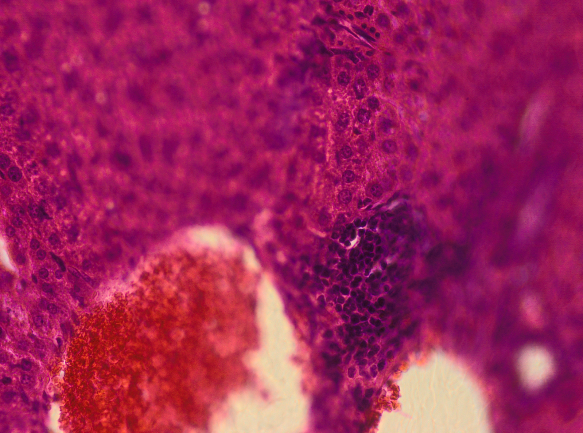 |
| 2 | - Nodular infiltrates - Capsular organization around infiltrates - Absence of necrotic tissue | 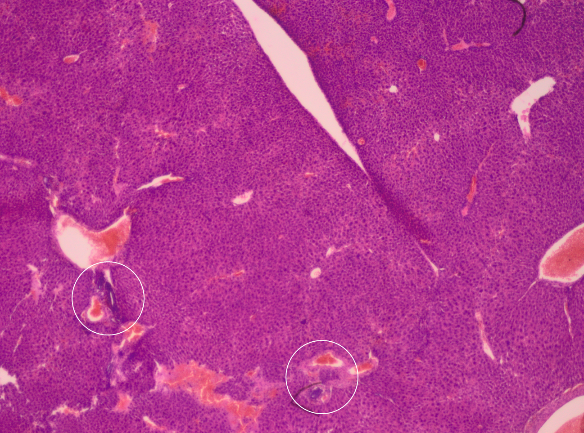 | 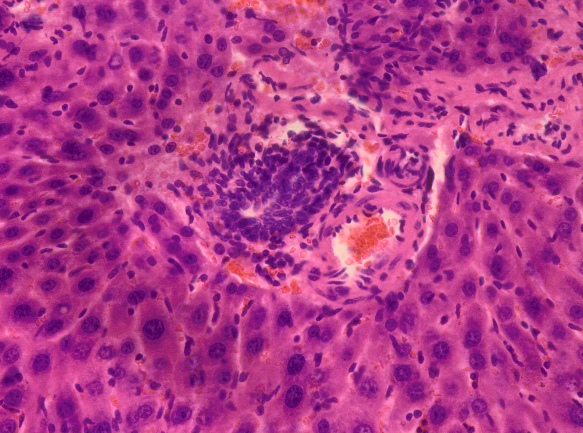 |
| 3 | - Nodular infiltrates - Presence of necrotic tissue | 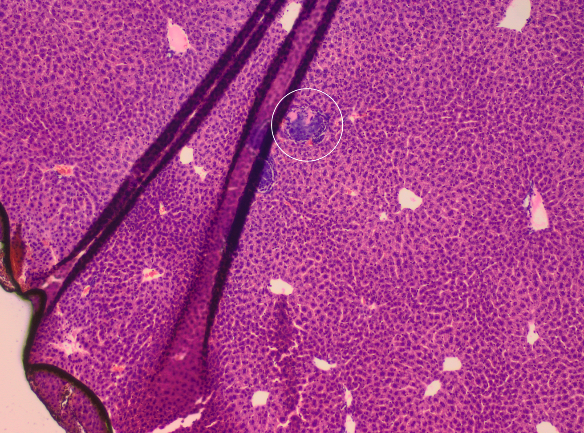 | 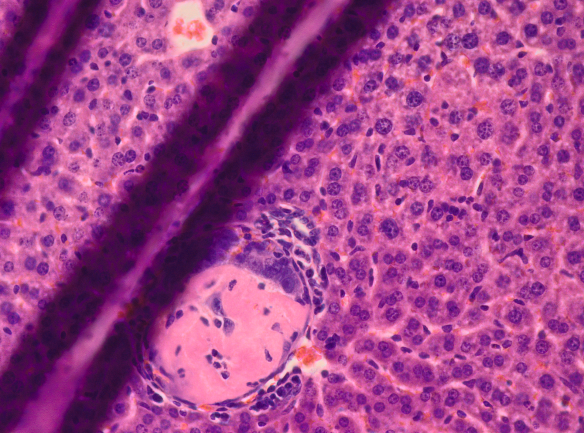 |
| 4 | - Large nodular infiltrates - Large patches of necrotic tissue in nodules - Clear distinct capsular organization - Less than ¼ of the liver coupe consist of tumor | 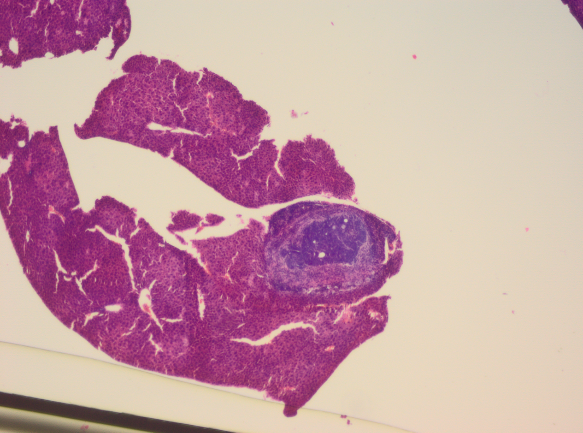 | 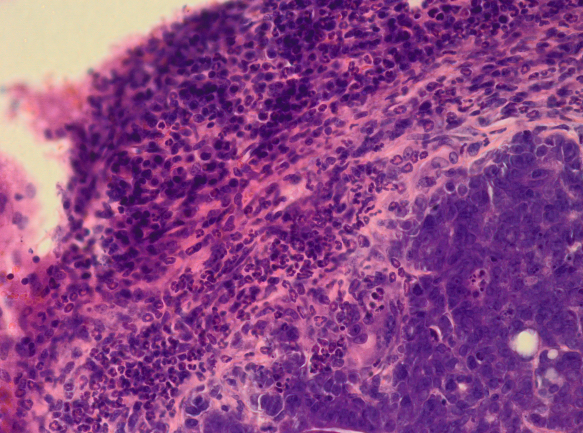 |
| 5 | - Large nodular infiltrates - Large patches of necrotic tissue in nodules - Clear distinct capsular organization - More than ¼ of the liver coupe consist of tumor | 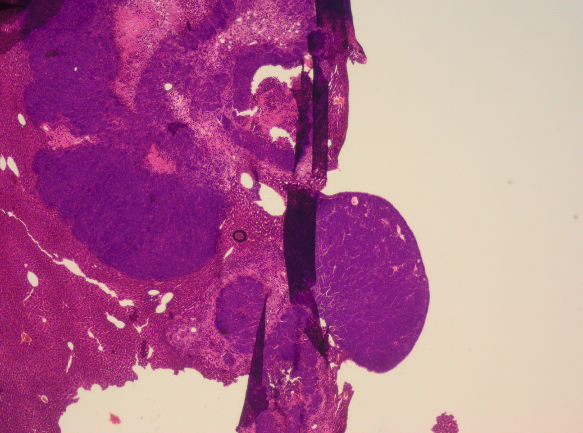 | 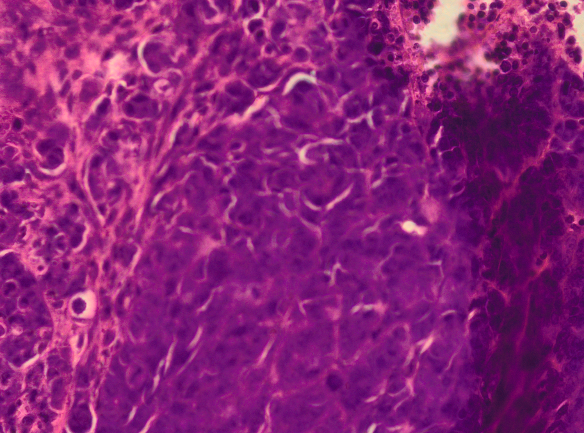 |

| **Lung scoring system** | | | |
| --- | --- | --- | --- |
| **Score** | **Parameters** | **10x Magnification** | **40x Magnification** |
| 0 | - Well-structured cells - Absence of tumor nodule(s) - Absence of necrotic tissue | 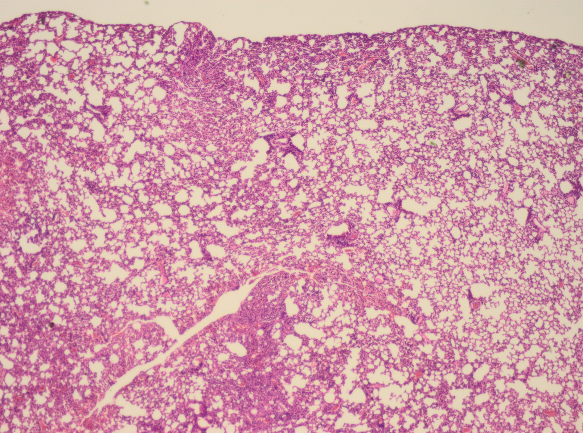 |  |
| 1 | - Nodular loose infiltrates - Absence of necrotic tissue | 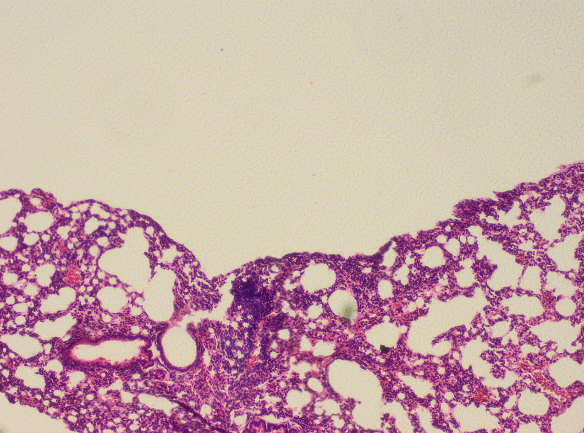 | 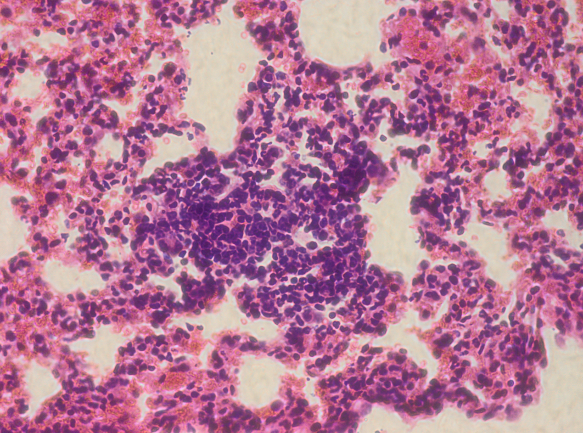 |
| 2 | - Nodular dense infiltrates - Decrease of cytoplasm/nucleus ratio - Absence of necrotic tissue | 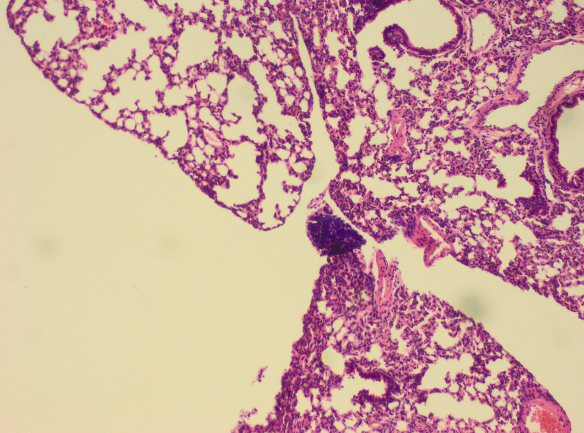 | 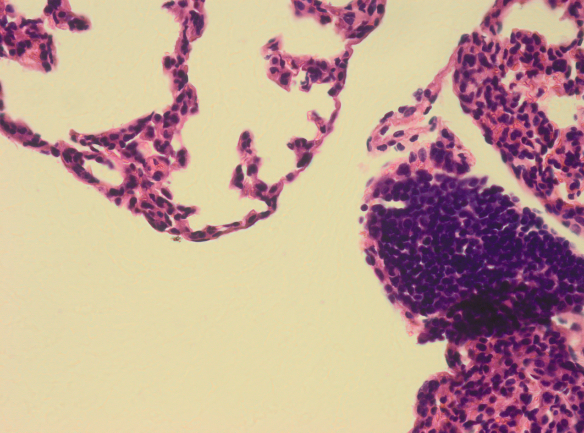 |
| 3 | - Large nodular infiltrates - Cytoplasmic basophilia - Absence of necrotic tissue | 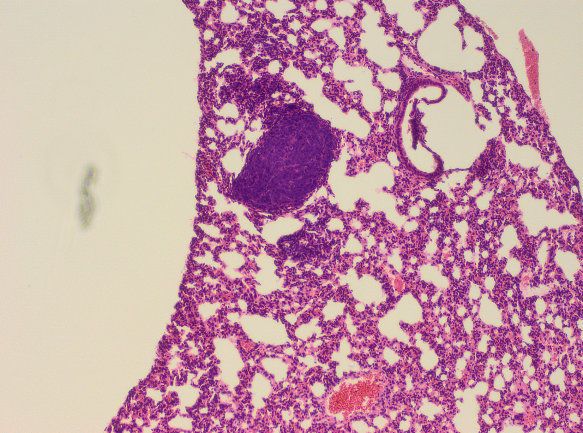 | 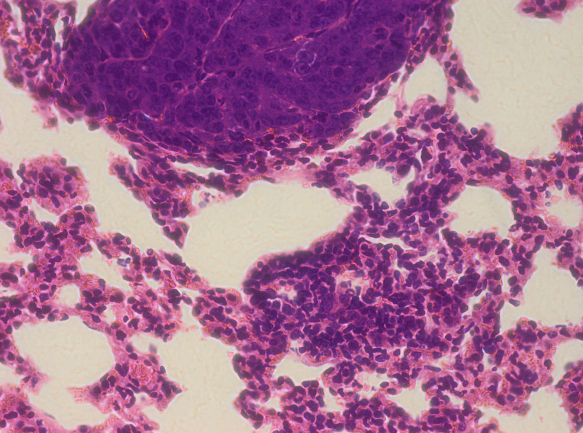 |
| 4 | - Nodular infiltrates - Presence of necrotic tissue | 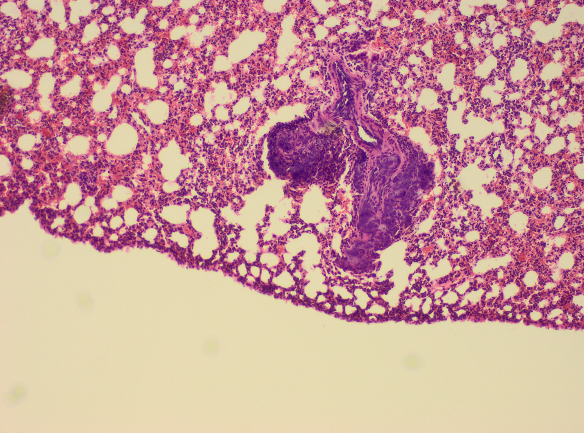 | 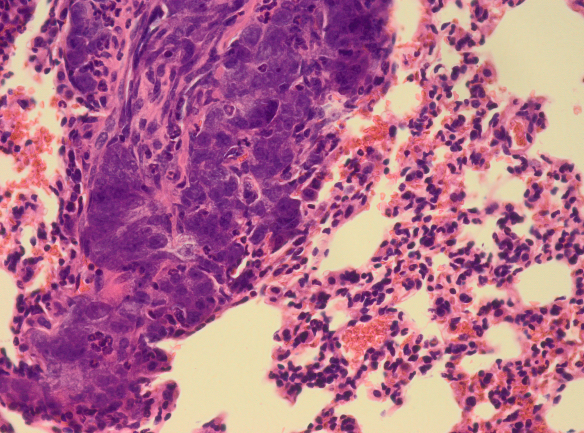 |
| 5 | - More than one necrotic infiltrate | 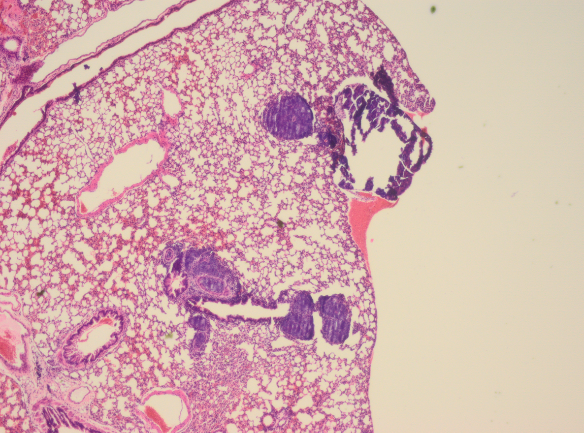 | 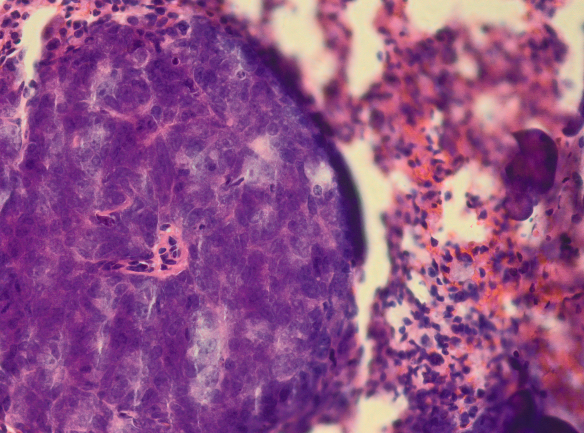 |

**Table S4. Histopathological scoring system.** For both liver and lung tissues, a scoring system was developed to evaluate the presence and severity of tumor nodules and necrotic tissue. Tissues were classified based on the severity of histopathological features: normal tissues were given a score = 0, while the presence of clear (large) and numerous tumor nodules and necrotic tissues corresponded to a score = 5.
