# Supplementary figures and images for: The quorum sensing peptide EntF* promotes colorectal cancer metastasis in mice: a new factor in the host-microbiome interaction (part 1 of 2)
Source: BMC Biol. 2022 Jun 27;20:151. doi: 10.1186/s12915-022-01317-z (PMC9238271; doi:10.1186/s12915-022-01317-z)

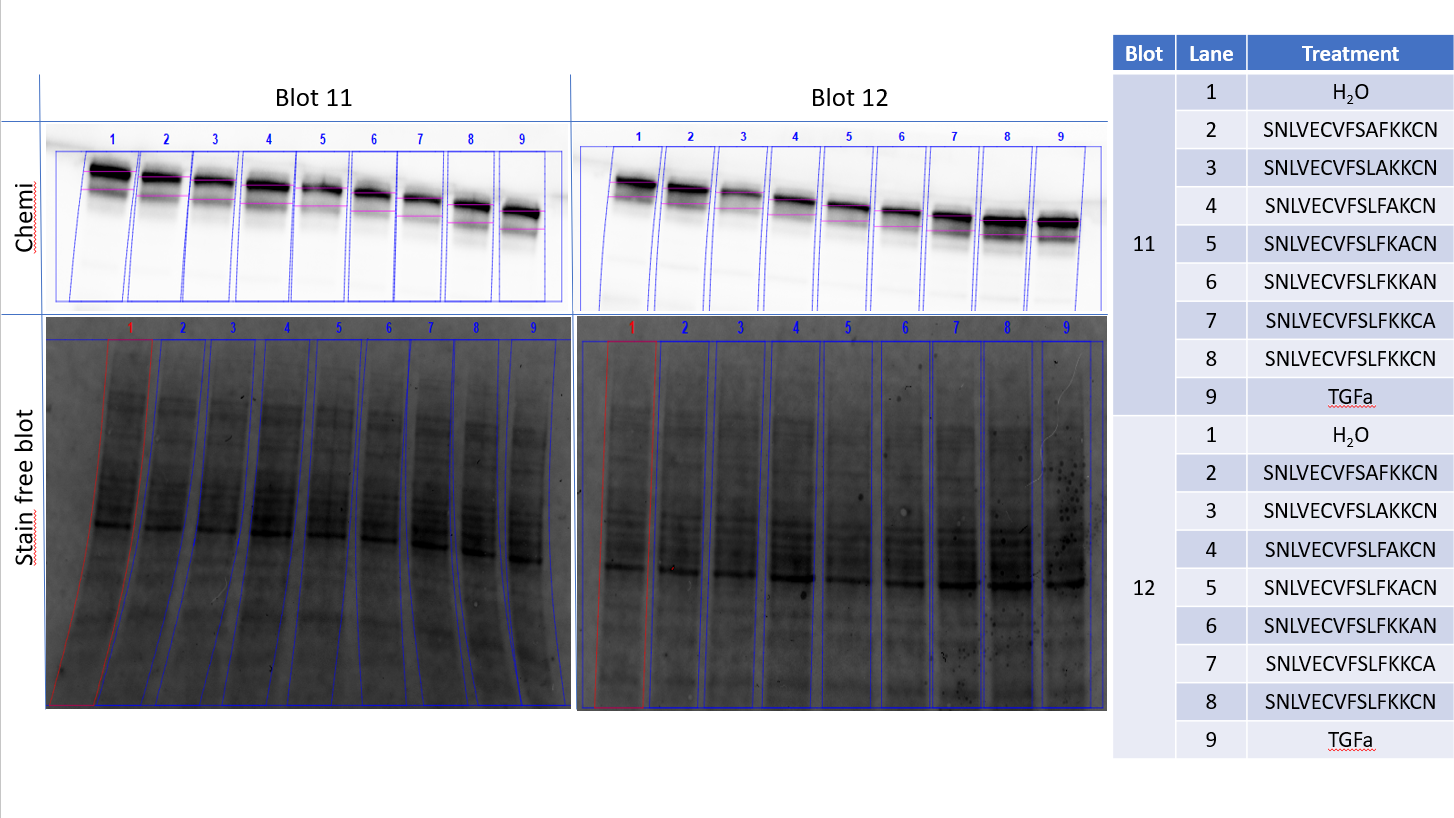

Supplement: Supplementary file 11 — Additional file 11. Raw data files Western Blot Alanine analogues. [file 12915_2022_1317_MOESM11_ESM.zip › AlanineSCAN/Blot11and12.PNG]

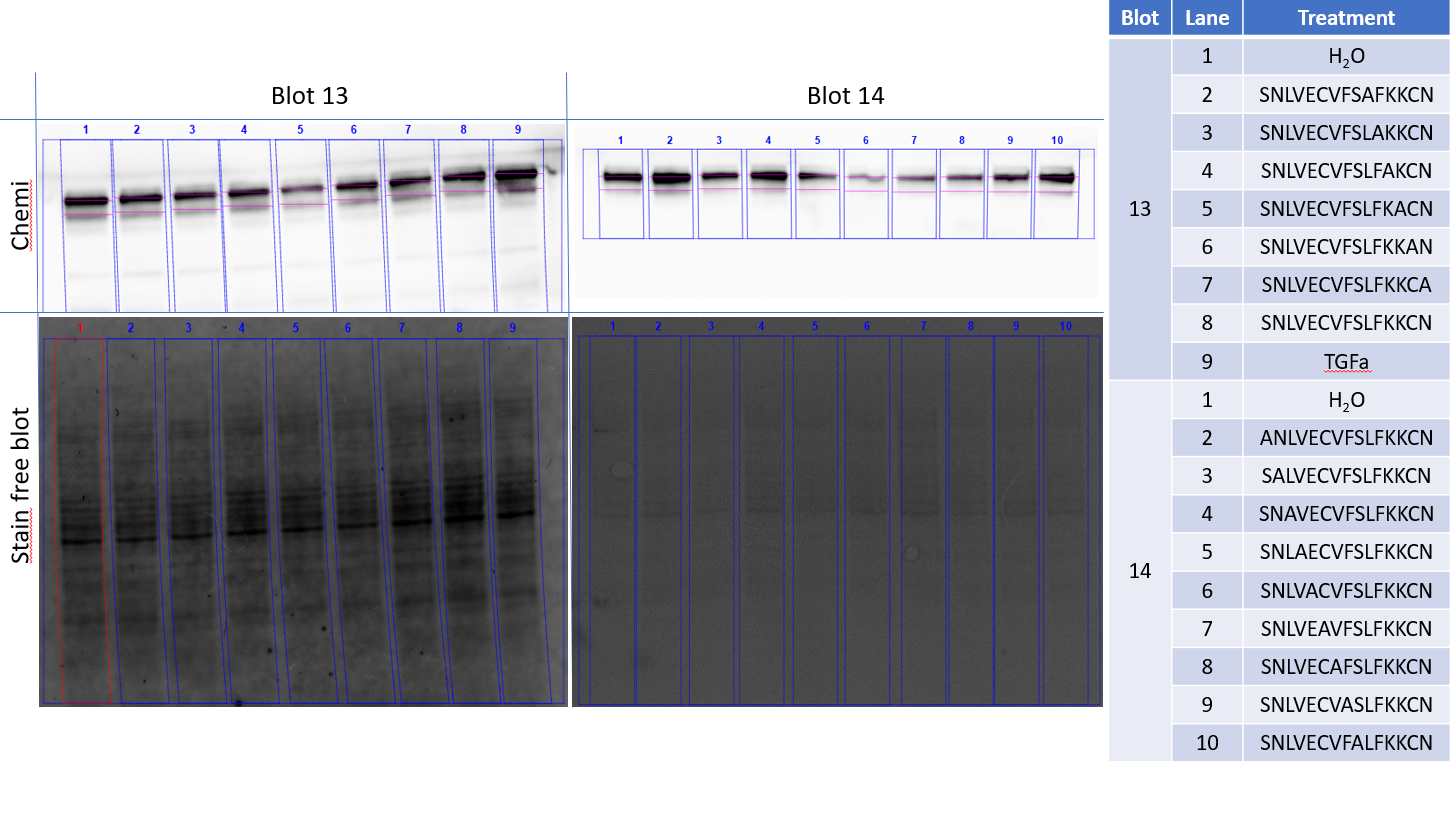

Supplement: Supplementary file 11 — Additional file 11. Raw data files Western Blot Alanine analogues. [file 12915_2022_1317_MOESM11_ESM.zip › AlanineSCAN/Blot13and14.PNG]

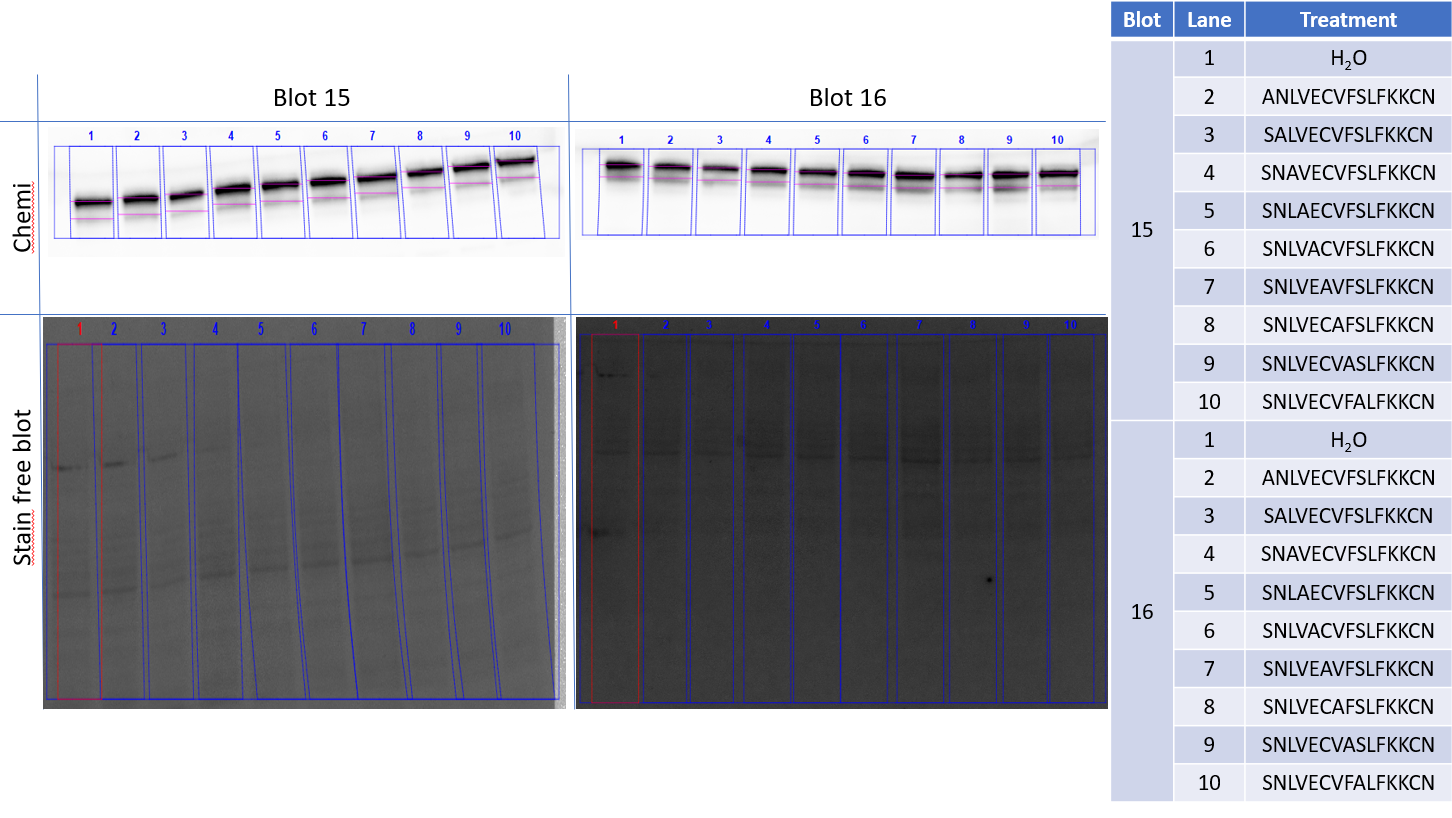

Supplement: Supplementary file 11 — Additional file 11. Raw data files Western Blot Alanine analogues. [file 12915_2022_1317_MOESM11_ESM.zip › AlanineSCAN/Blot15and16.PNG]

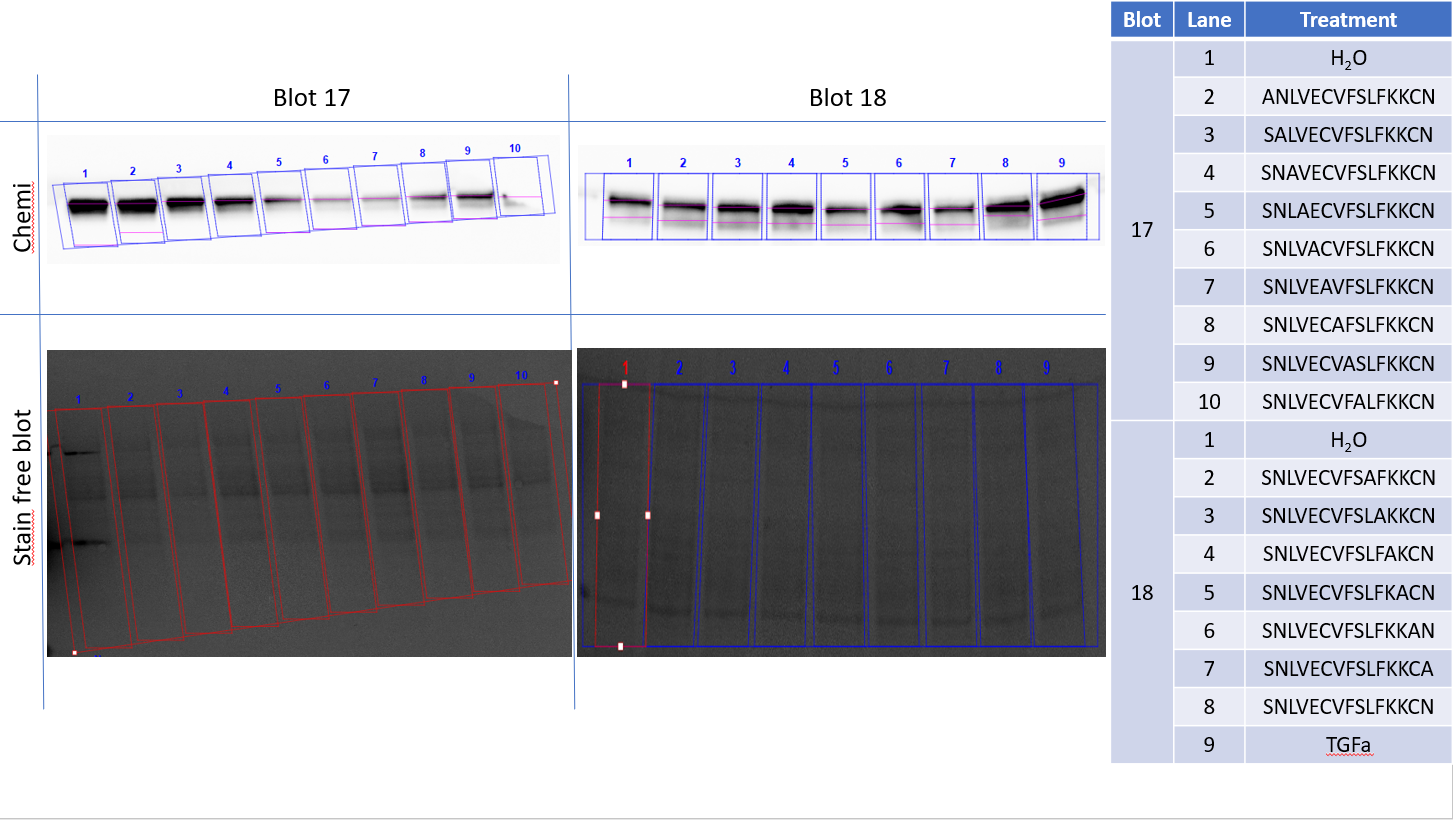

Supplement: Supplementary file 11 — Additional file 11. Raw data files Western Blot Alanine analogues. [file 12915_2022_1317_MOESM11_ESM.zip › AlanineSCAN/Blot17and18.PNG]

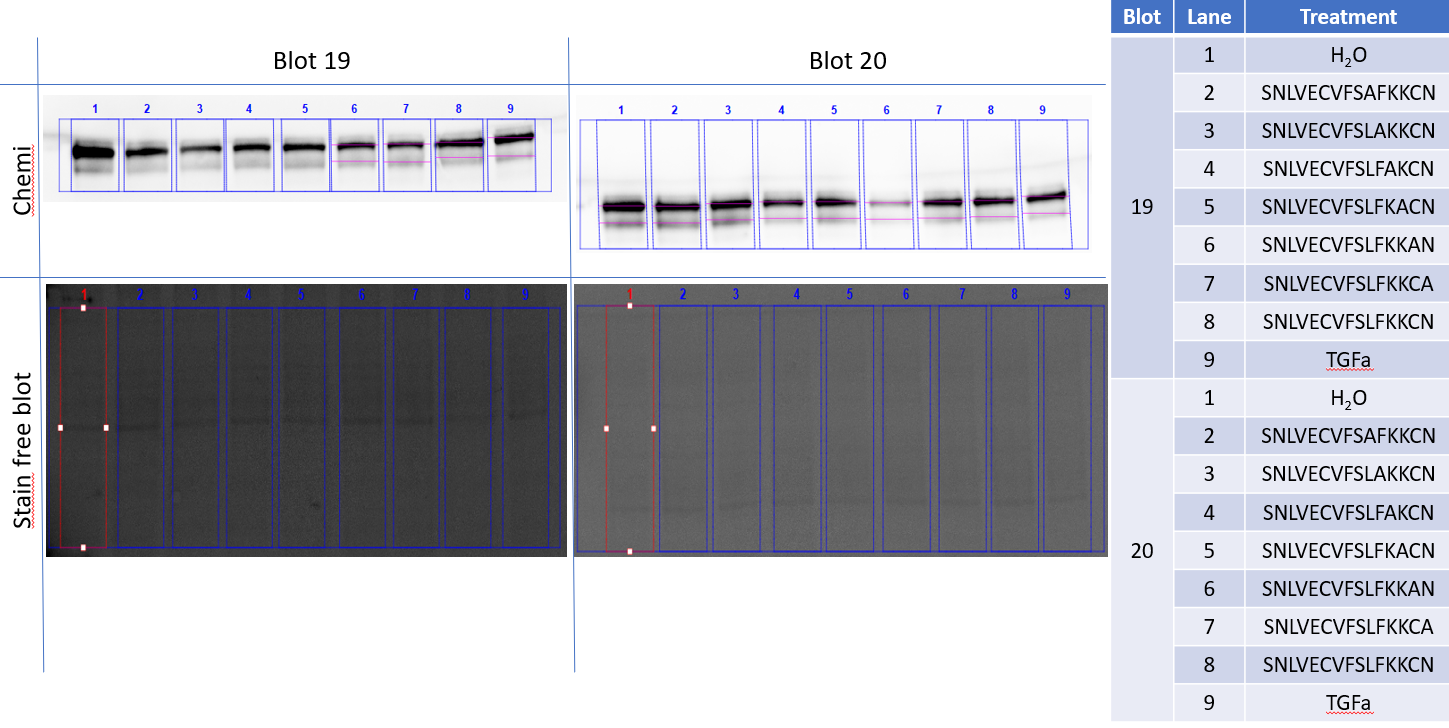

Supplement: Supplementary file 11 — Additional file 11. Raw data files Western Blot Alanine analogues. [file 12915_2022_1317_MOESM11_ESM.zip › AlanineSCAN/Blot19and20.PNG]

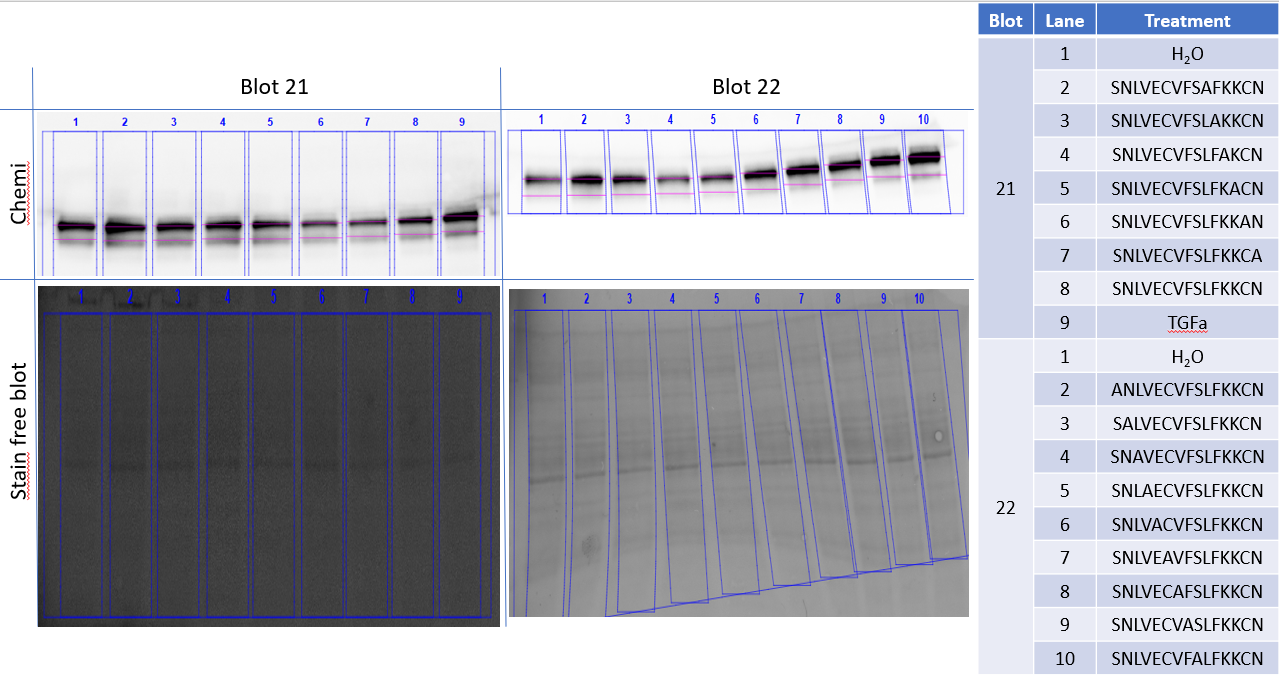

Supplement: Supplementary file 11 — Additional file 11. Raw data files Western Blot Alanine analogues. [file 12915_2022_1317_MOESM11_ESM.zip › AlanineSCAN/Blot21and22.PNG]

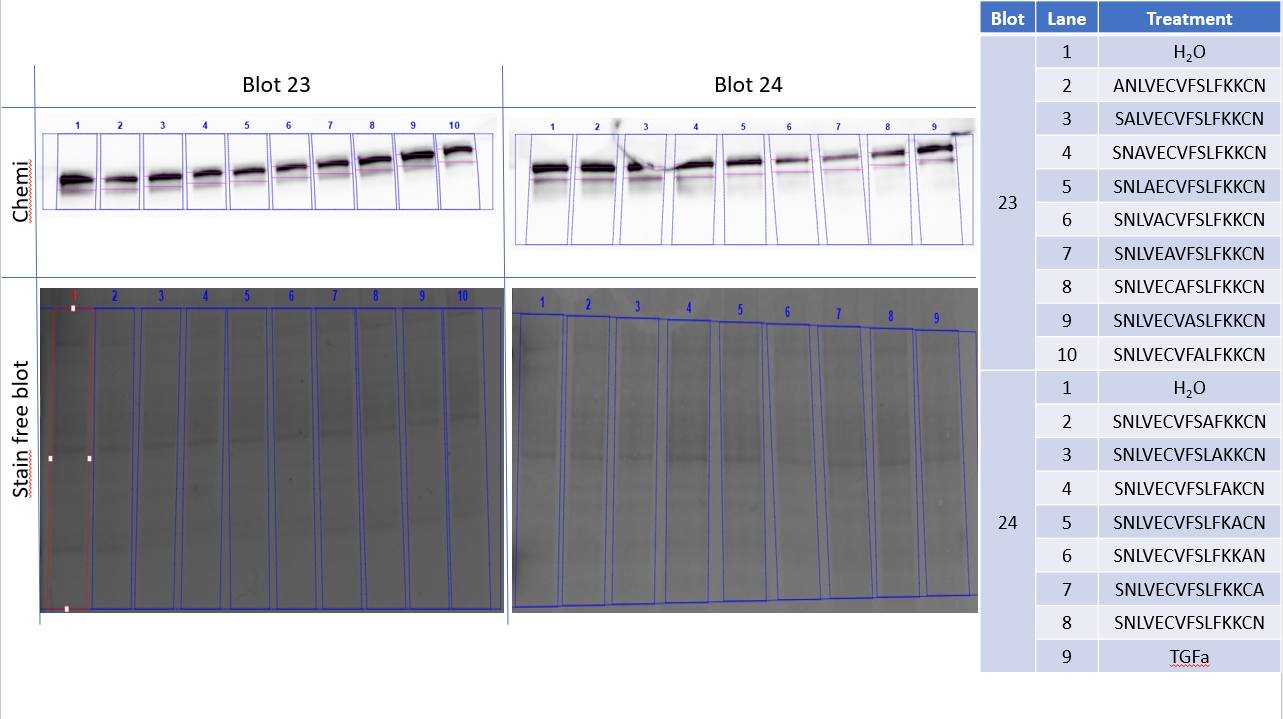

Supplement: Supplementary file 11 — Additional file 11. Raw data files Western Blot Alanine analogues. [file 12915_2022_1317_MOESM11_ESM.zip › AlanineSCAN/Blot23and24.PNG]

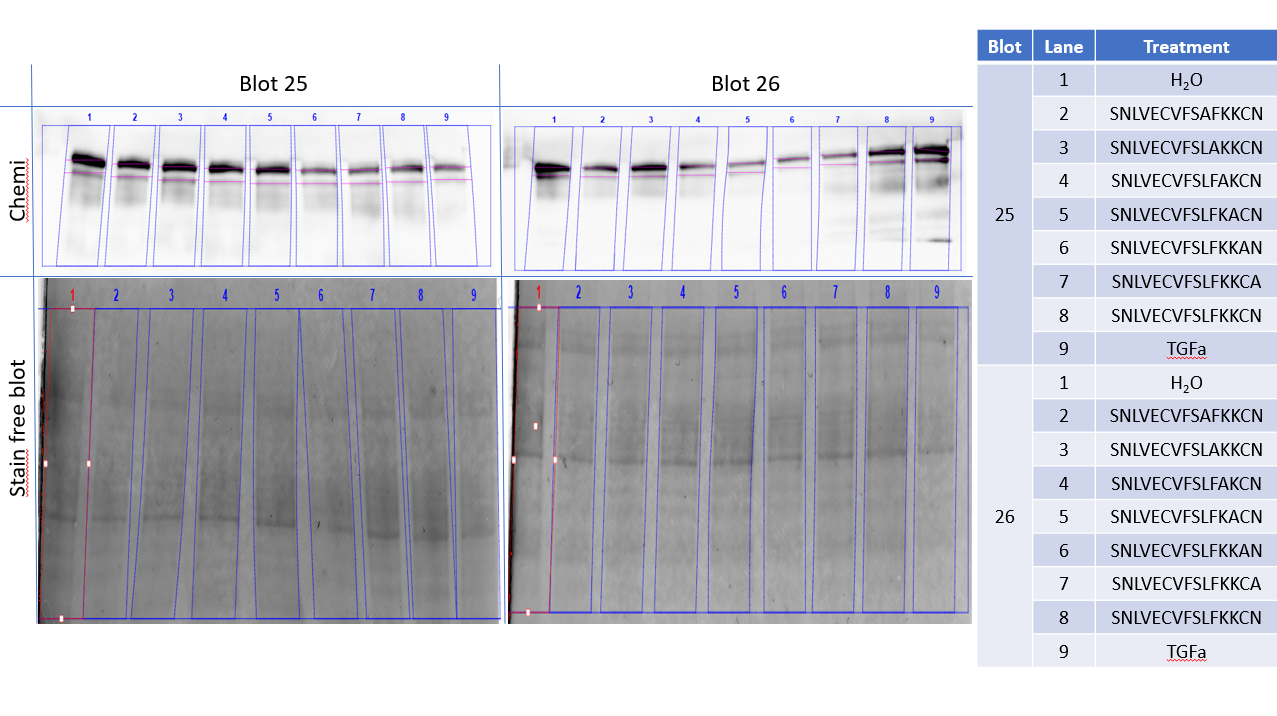

Supplement: Supplementary file 11 — Additional file 11. Raw data files Western Blot Alanine analogues. [file 12915_2022_1317_MOESM11_ESM.zip › AlanineSCAN/Blot25and26.PNG]

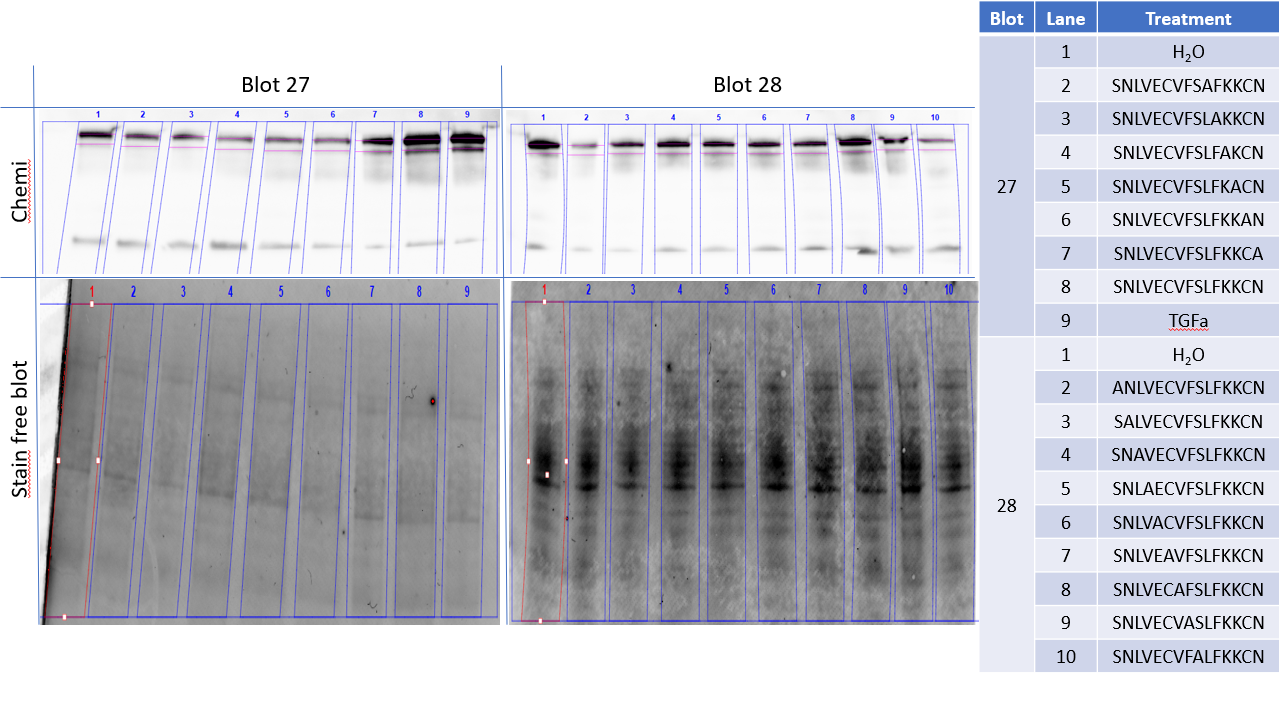

Supplement: Supplementary file 11 — Additional file 11. Raw data files Western Blot Alanine analogues. [file 12915_2022_1317_MOESM11_ESM.zip › AlanineSCAN/Blot27and28.PNG]

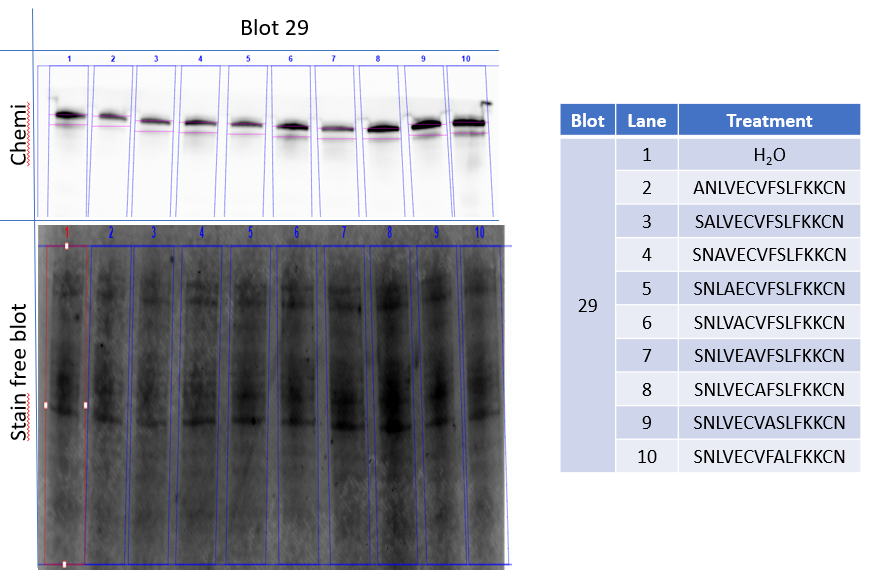

Supplement: Supplementary file 11 — Additional file 11. Raw data files Western Blot Alanine analogues. [file 12915_2022_1317_MOESM11_ESM.zip › AlanineSCAN/Blot29.PNG]

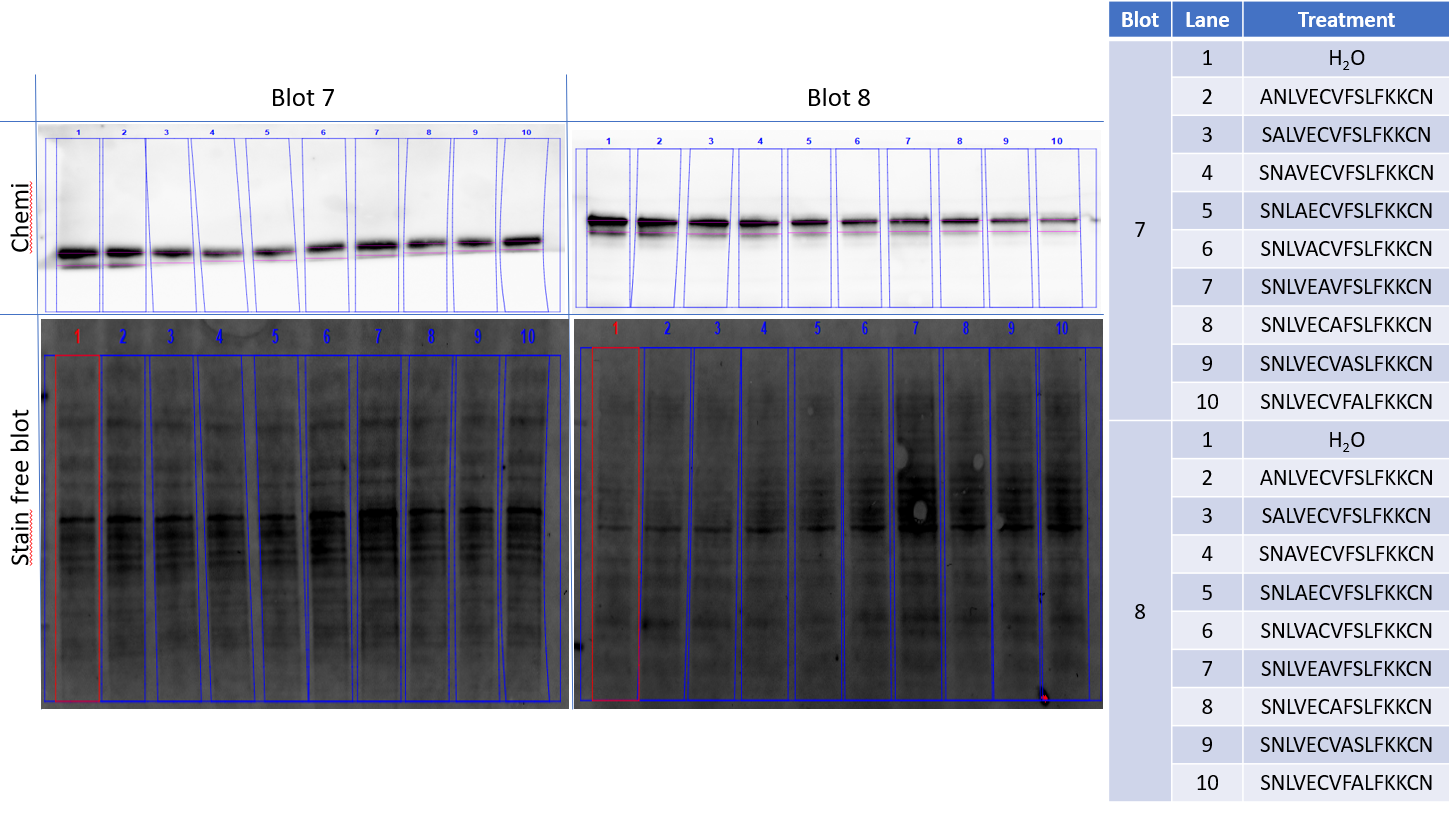

Supplement: Supplementary file 11 — Additional file 11. Raw data files Western Blot Alanine analogues. [file 12915_2022_1317_MOESM11_ESM.zip › AlanineSCAN/Blot7and8.PNG]

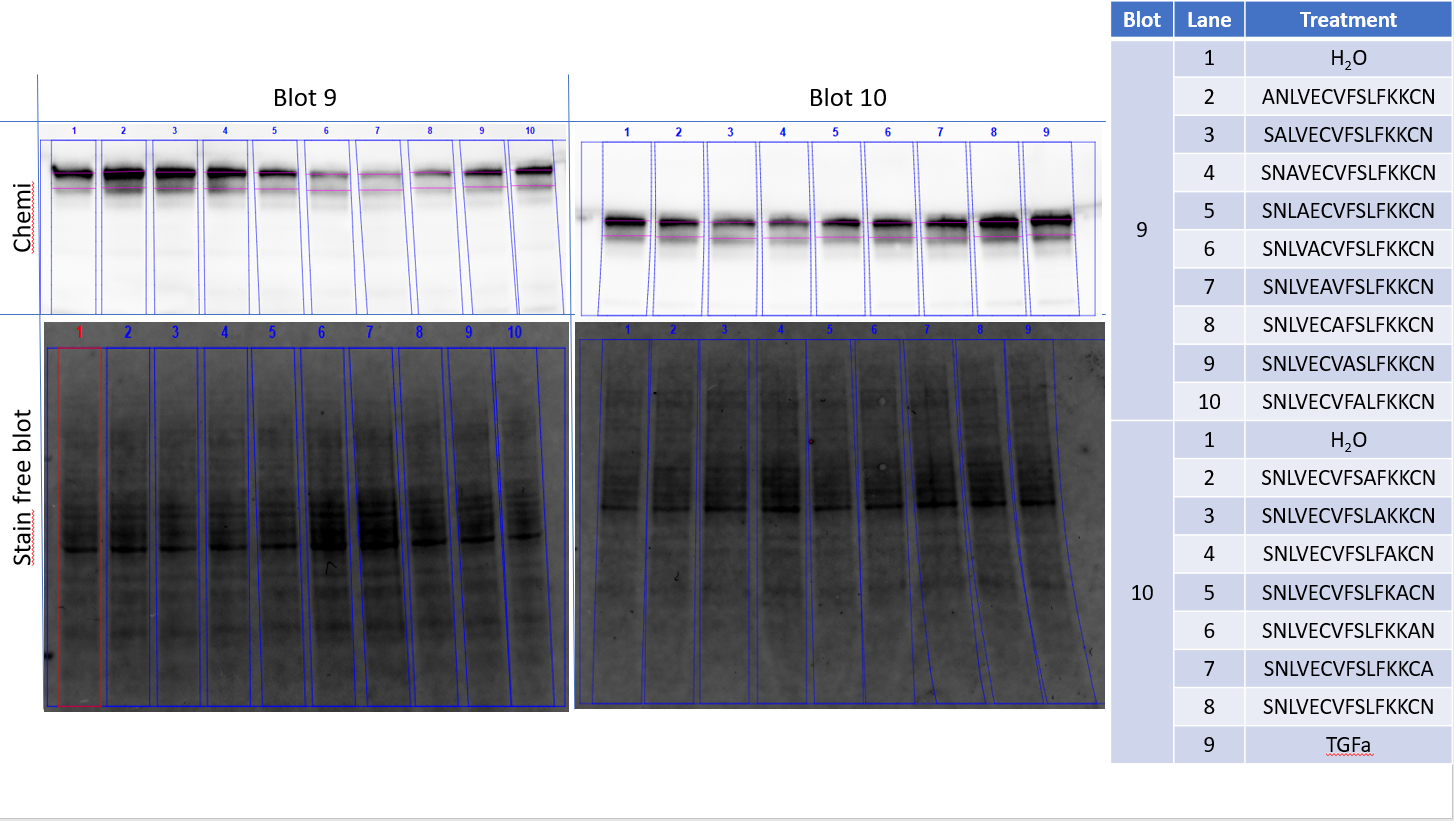

Supplement: Supplementary file 11 — Additional file 11. Raw data files Western Blot Alanine analogues. [file 12915_2022_1317_MOESM11_ESM.zip › AlanineSCAN/Blot9and10.PNG]

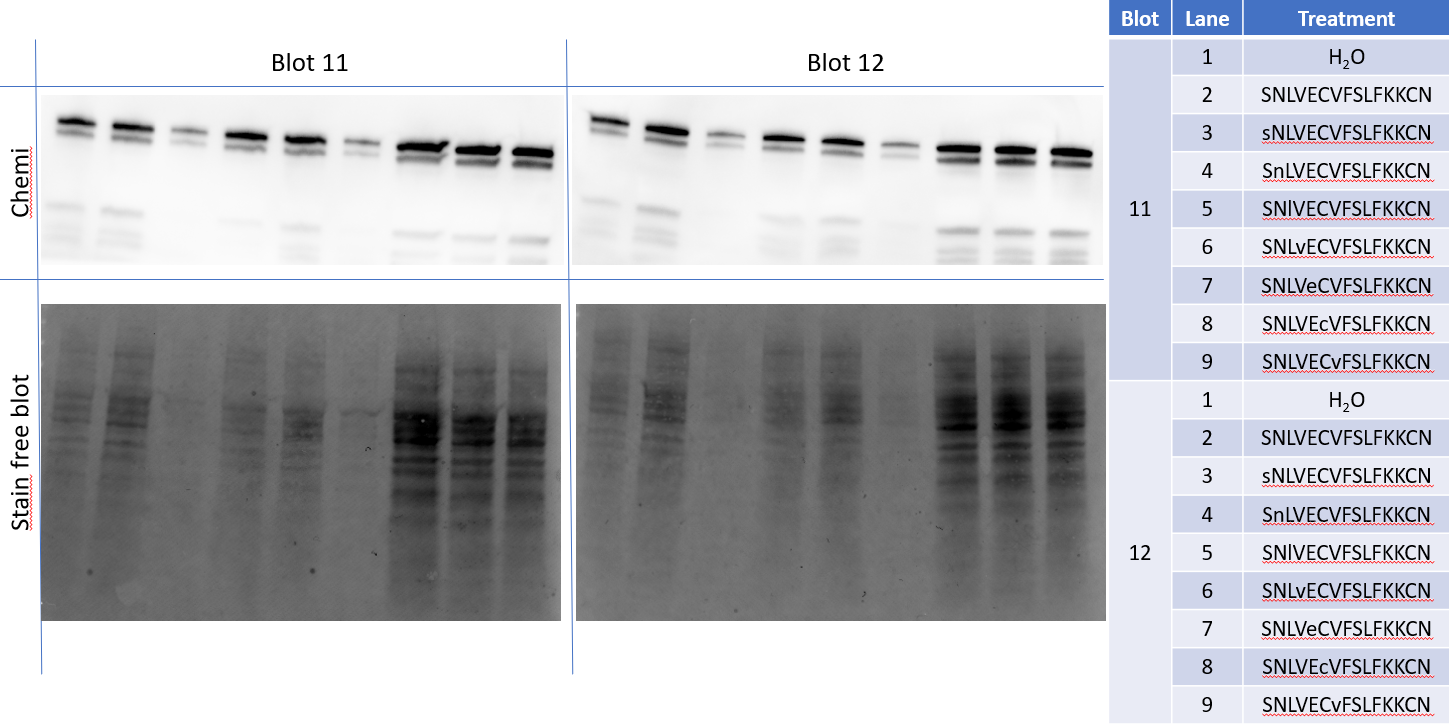

Supplement: Supplementary file 12 — Additional file 12. Raw data files Western Blot D-amino acid analogues. [file 12915_2022_1317_MOESM12_ESM.zip › Dscan/blot11and12.PNG]

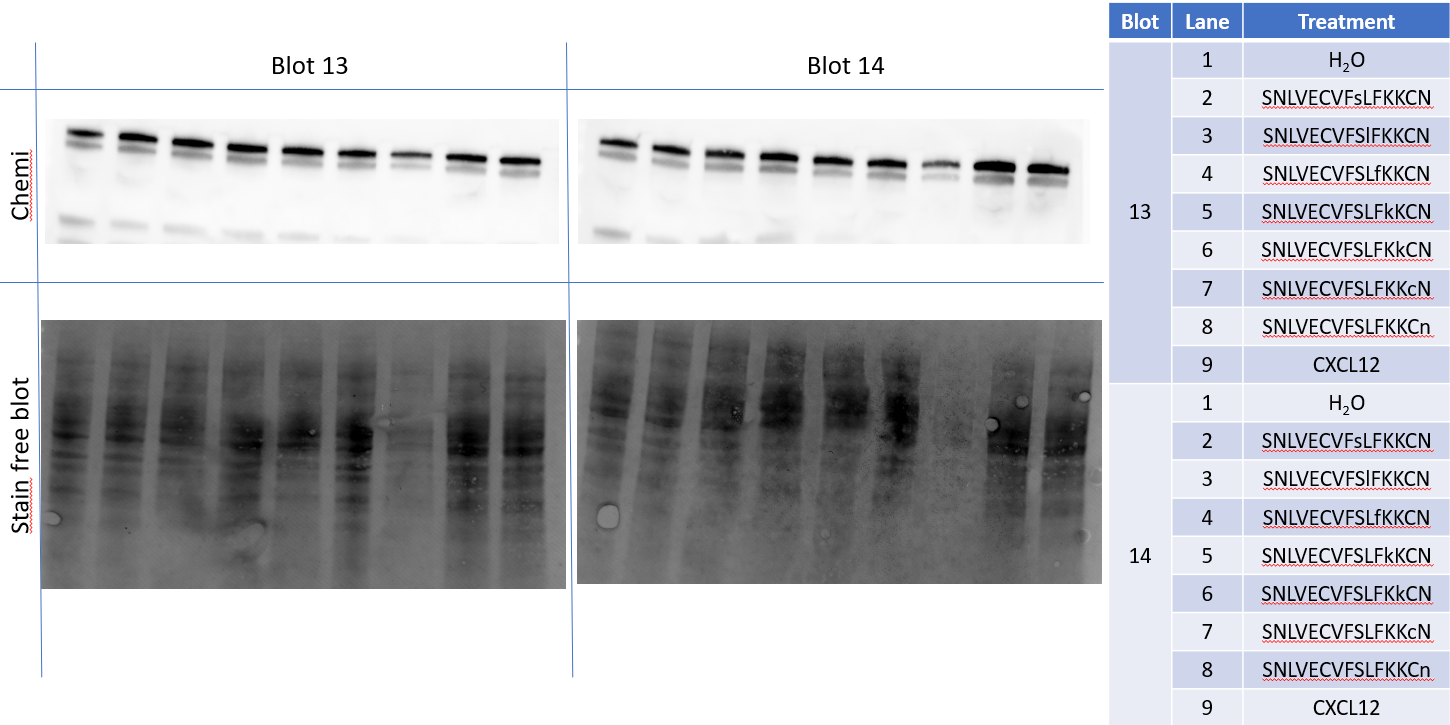

Supplement: Supplementary file 12 — Additional file 12. Raw data files Western Blot D-amino acid analogues. [file 12915_2022_1317_MOESM12_ESM.zip › Dscan/blot13and14.PNG]

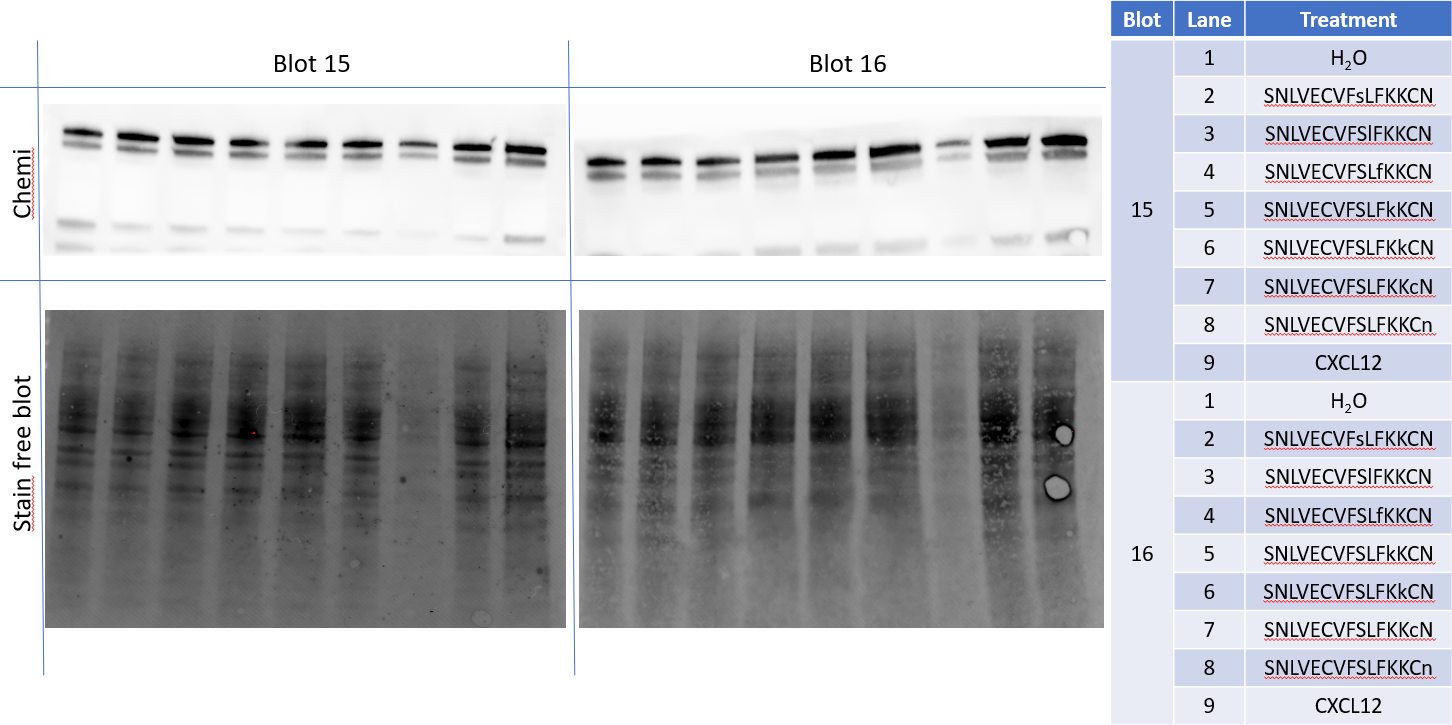

Supplement: Supplementary file 12 — Additional file 12. Raw data files Western Blot D-amino acid analogues. [file 12915_2022_1317_MOESM12_ESM.zip › Dscan/blot15and16.PNG]

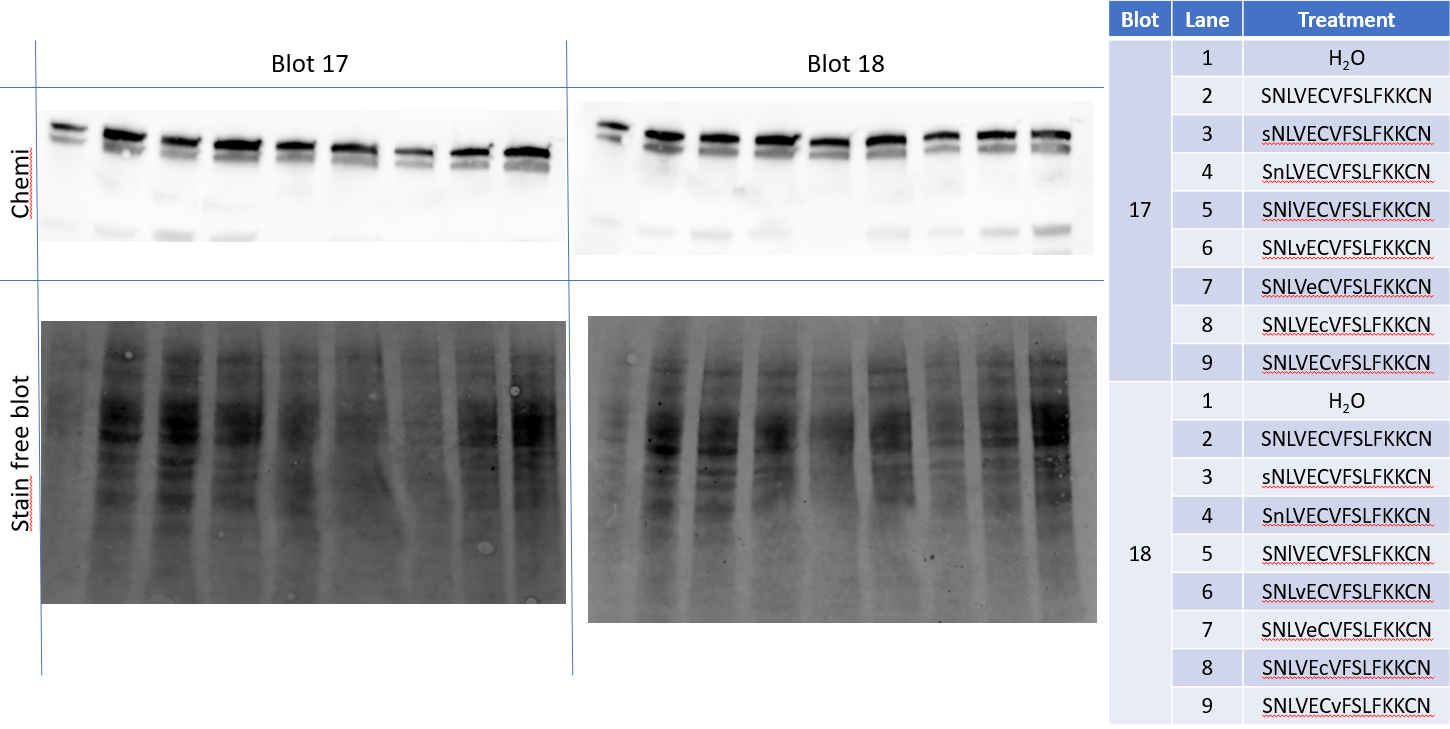

Supplement: Supplementary file 12 — Additional file 12. Raw data files Western Blot D-amino acid analogues. [file 12915_2022_1317_MOESM12_ESM.zip › Dscan/blot17and18.PNG]

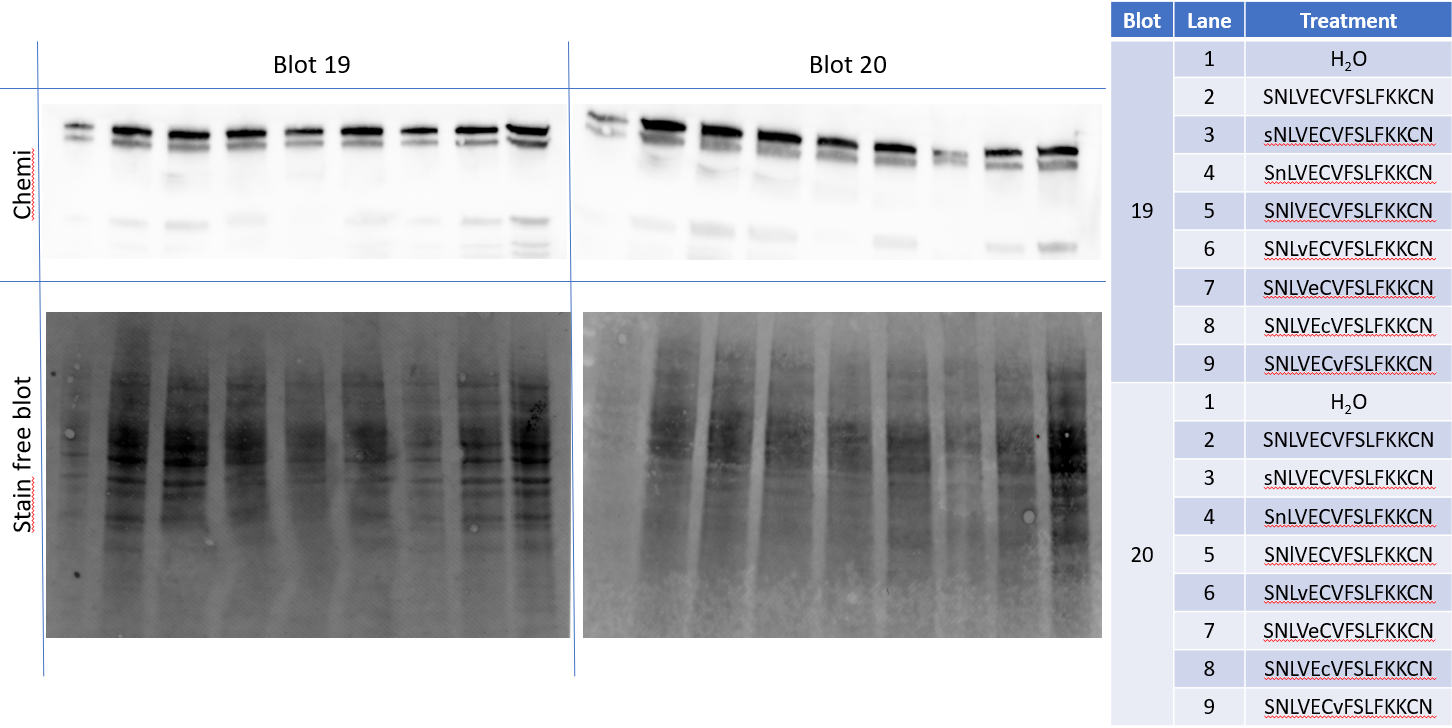

Supplement: Supplementary file 12 — Additional file 12. Raw data files Western Blot D-amino acid analogues. [file 12915_2022_1317_MOESM12_ESM.zip › Dscan/blot19and20.PNG]

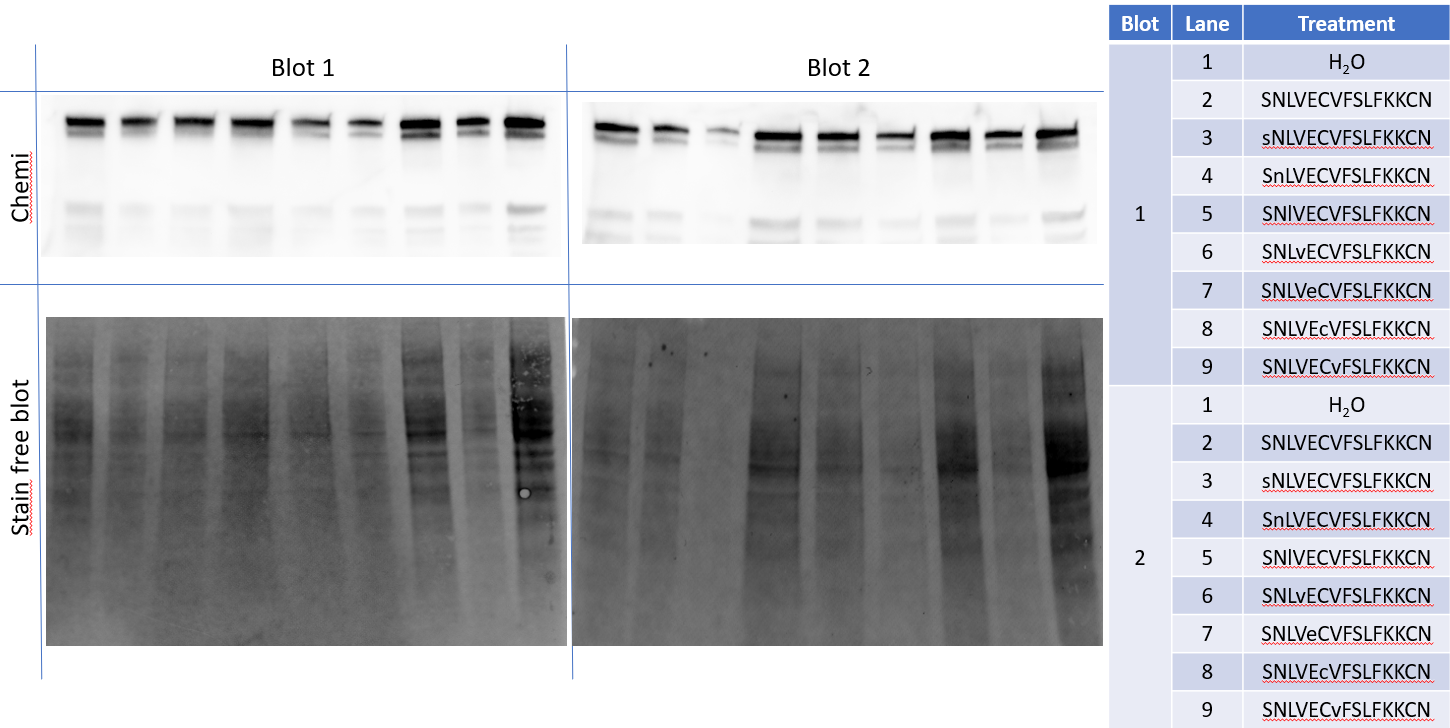

Supplement: Supplementary file 12 — Additional file 12. Raw data files Western Blot D-amino acid analogues. [file 12915_2022_1317_MOESM12_ESM.zip › Dscan/blot1and2.PNG]

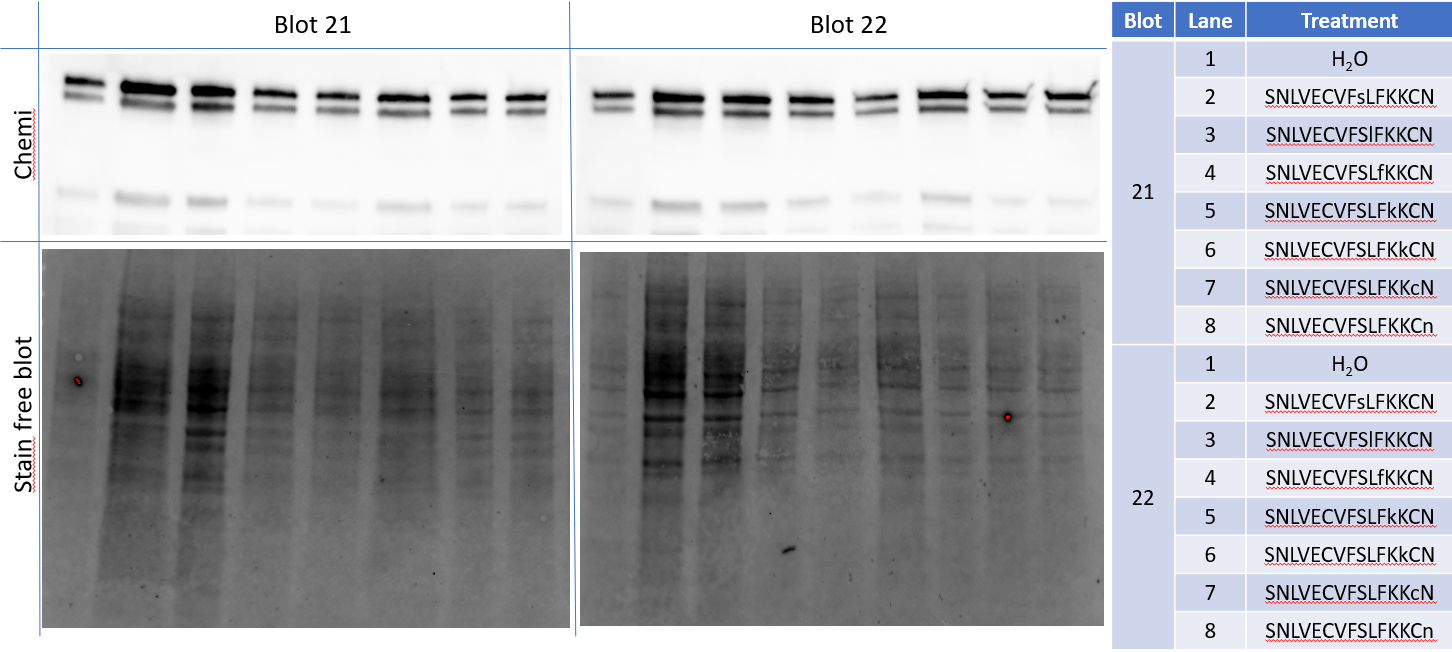

Supplement: Supplementary file 12 — Additional file 12. Raw data files Western Blot D-amino acid analogues. [file 12915_2022_1317_MOESM12_ESM.zip › Dscan/blot21and22.PNG]

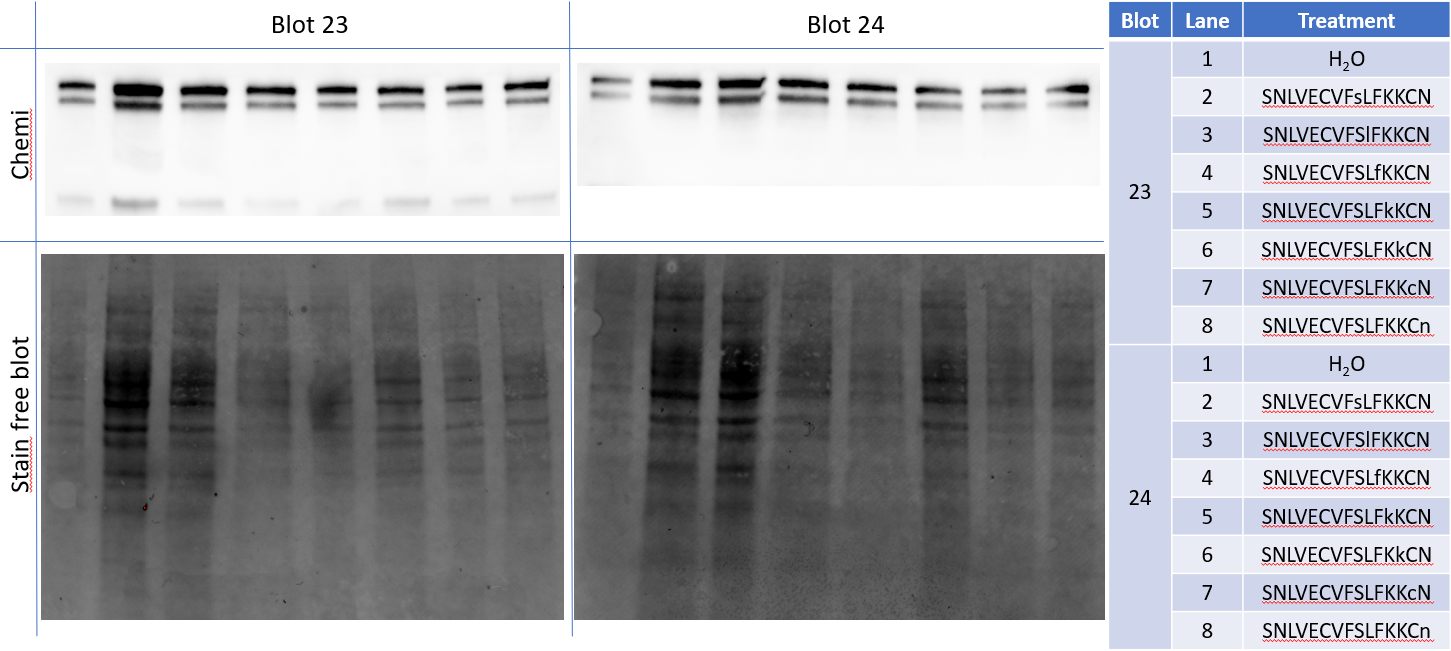

Supplement: Supplementary file 12 — Additional file 12. Raw data files Western Blot D-amino acid analogues. [file 12915_2022_1317_MOESM12_ESM.zip › Dscan/blot23and24.PNG]

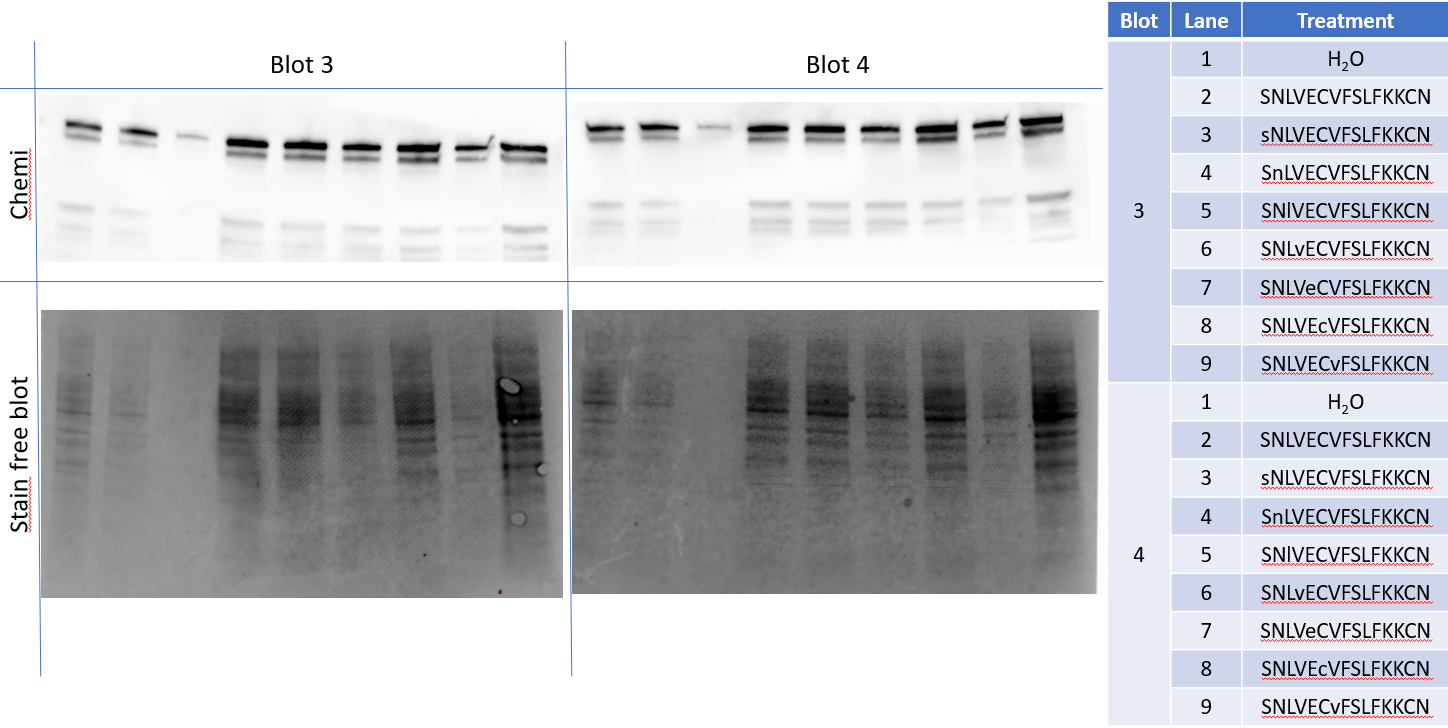

Supplement: Supplementary file 12 — Additional file 12. Raw data files Western Blot D-amino acid analogues. [file 12915_2022_1317_MOESM12_ESM.zip › Dscan/blot3and4.PNG]

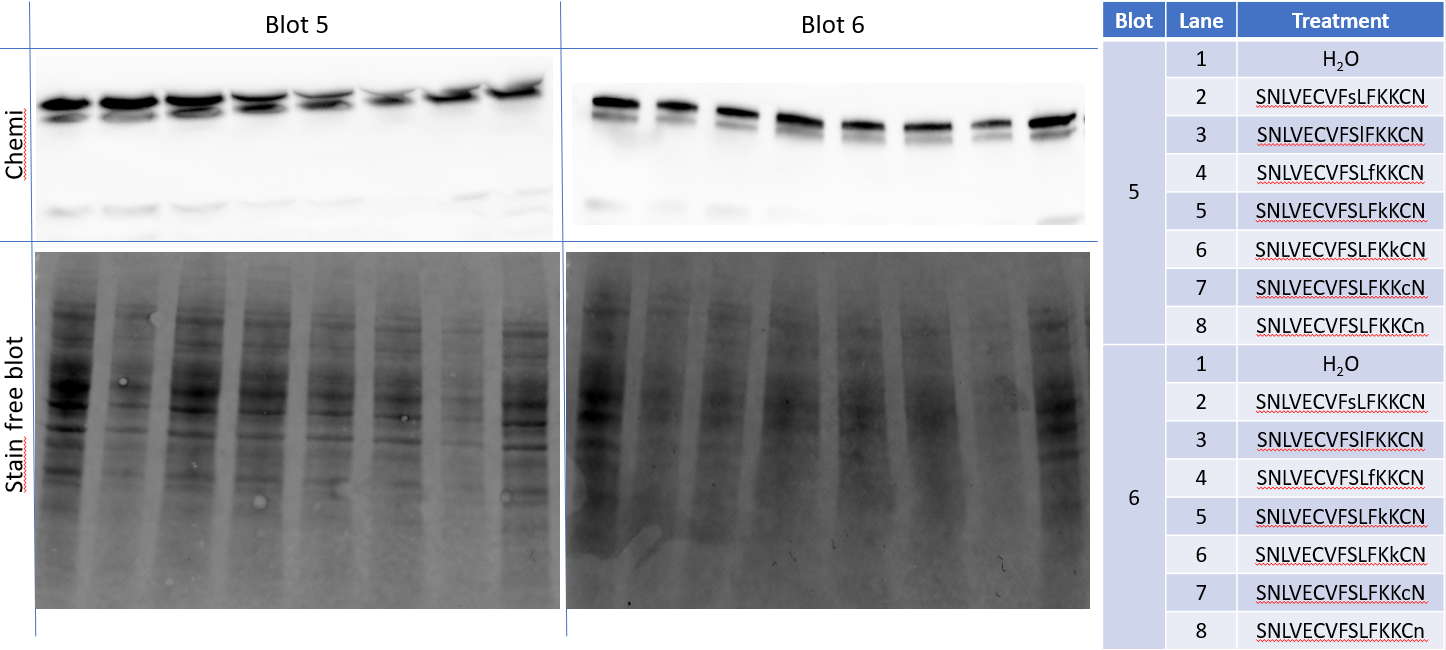

Supplement: Supplementary file 12 — Additional file 12. Raw data files Western Blot D-amino acid analogues. [file 12915_2022_1317_MOESM12_ESM.zip › Dscan/blot5and6.PNG]

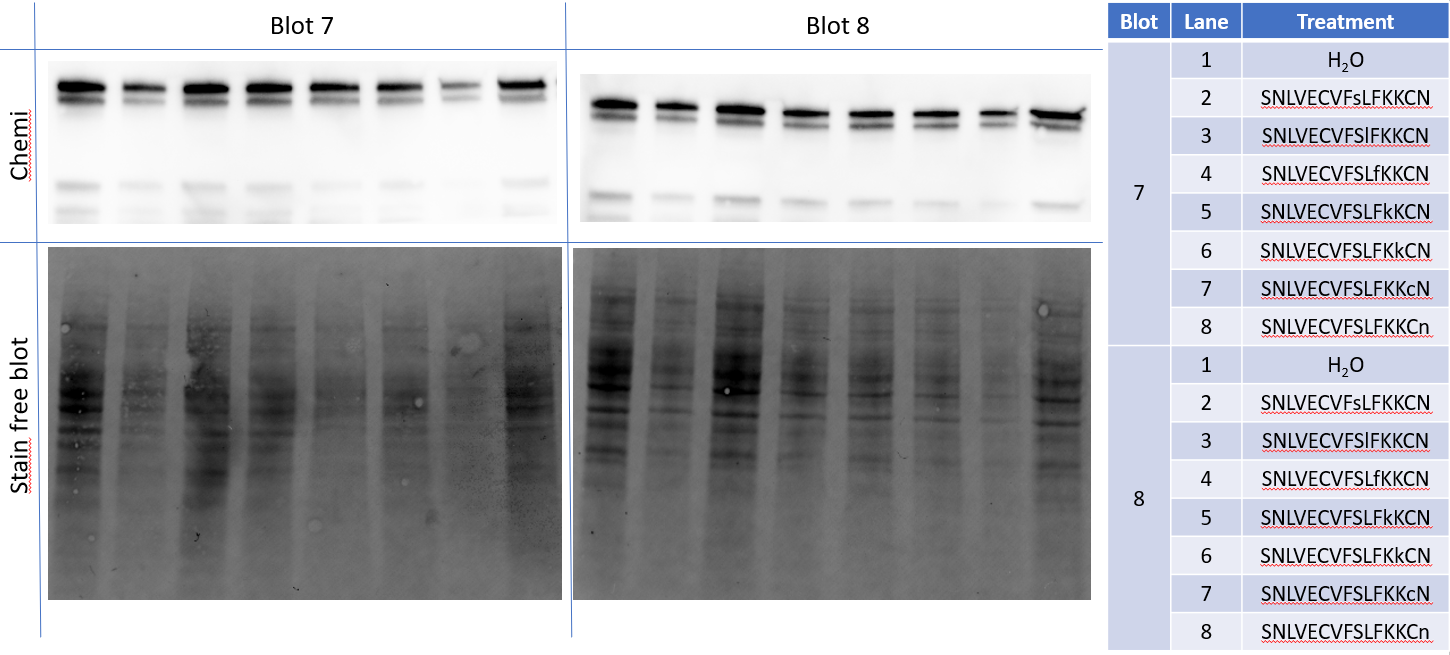

Supplement: Supplementary file 12 — Additional file 12. Raw data files Western Blot D-amino acid analogues. [file 12915_2022_1317_MOESM12_ESM.zip › Dscan/blot7and8.PNG]

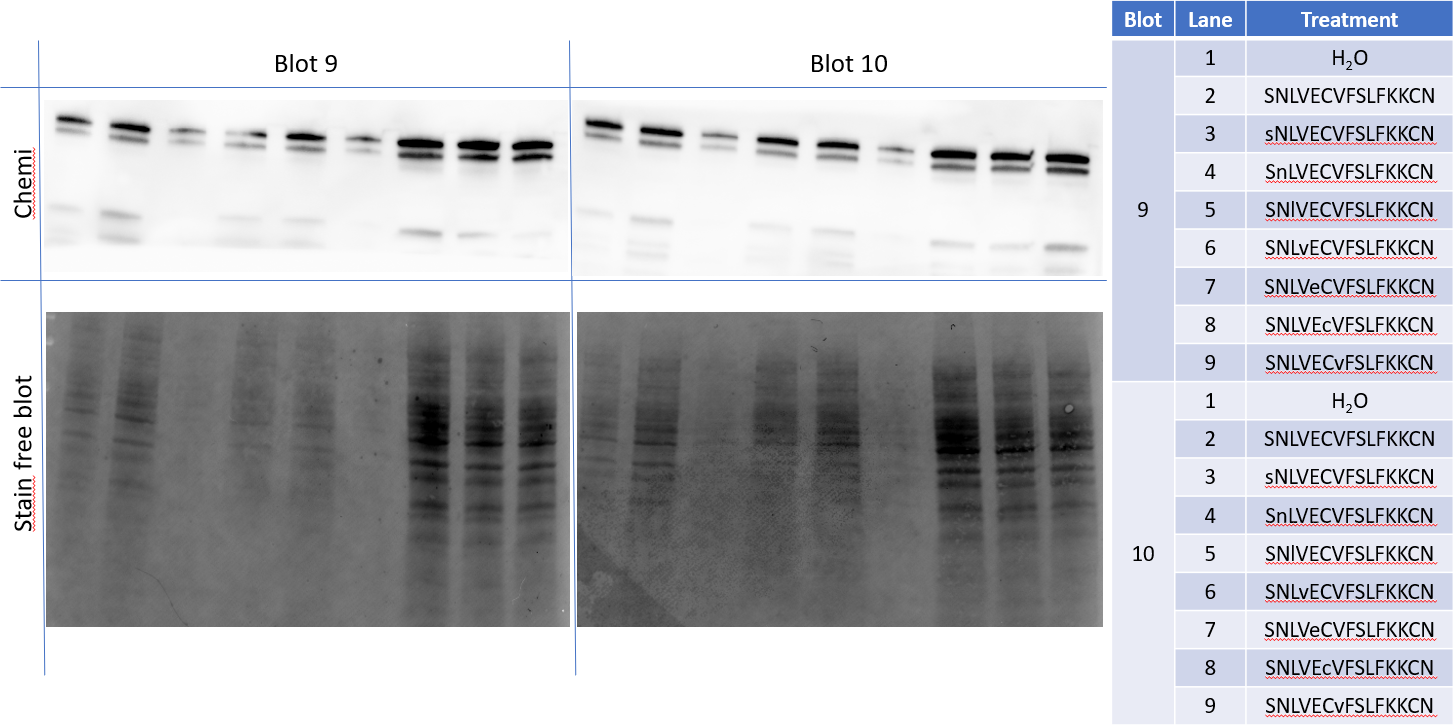

Supplement: Supplementary file 12 — Additional file 12. Raw data files Western Blot D-amino acid analogues. [file 12915_2022_1317_MOESM12_ESM.zip › Dscan/blot9and10.PNG]

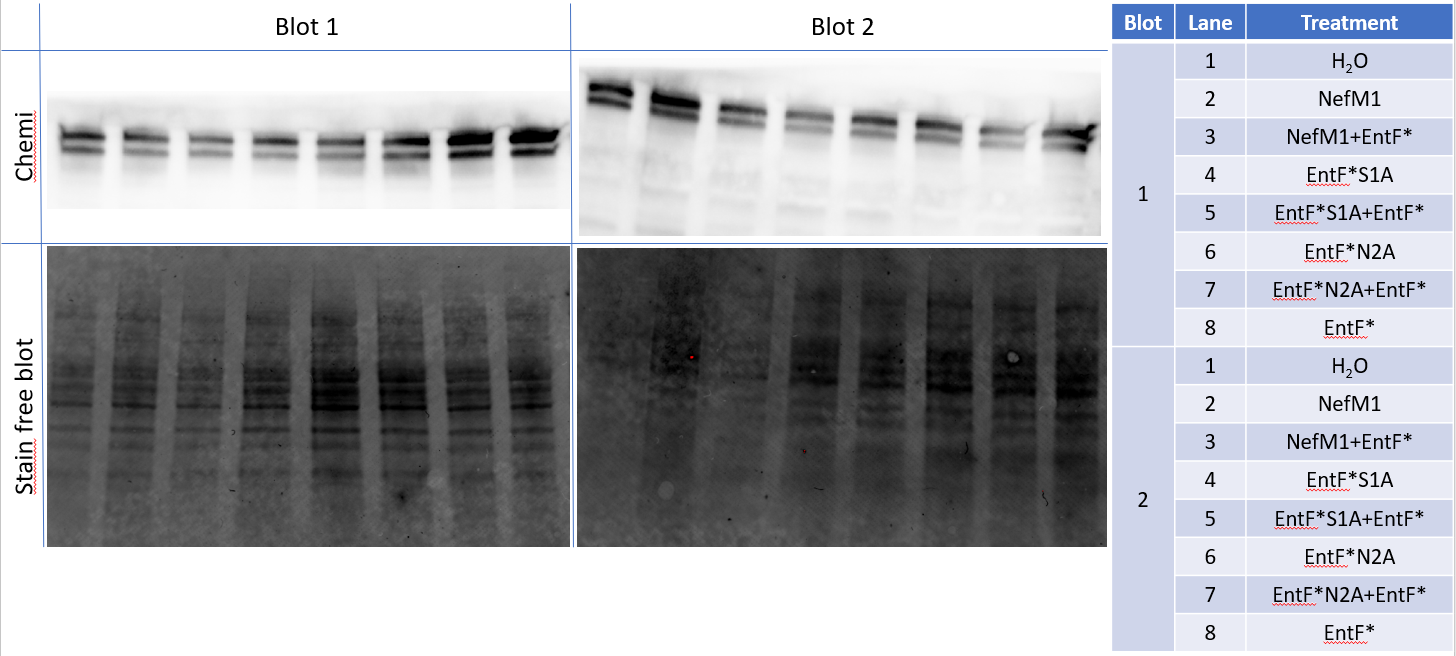

Supplement: Supplementary file 13 — Additional file 13. Raw data files Western Blot antagonist study. [file 12915_2022_1317_MOESM13_ESM.zip › Antagonist/blot1and2.PNG]

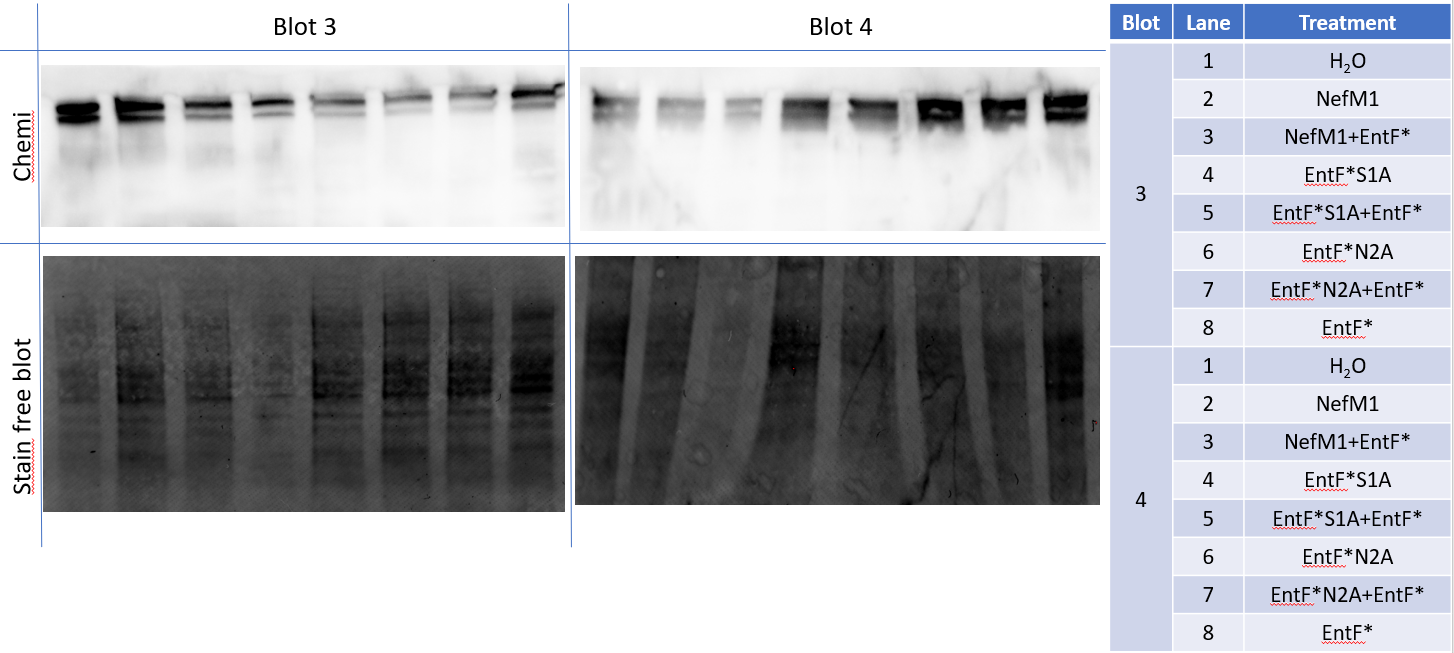

Supplement: Supplementary file 13 — Additional file 13. Raw data files Western Blot antagonist study. [file 12915_2022_1317_MOESM13_ESM.zip › Antagonist/blot3and4.PNG]

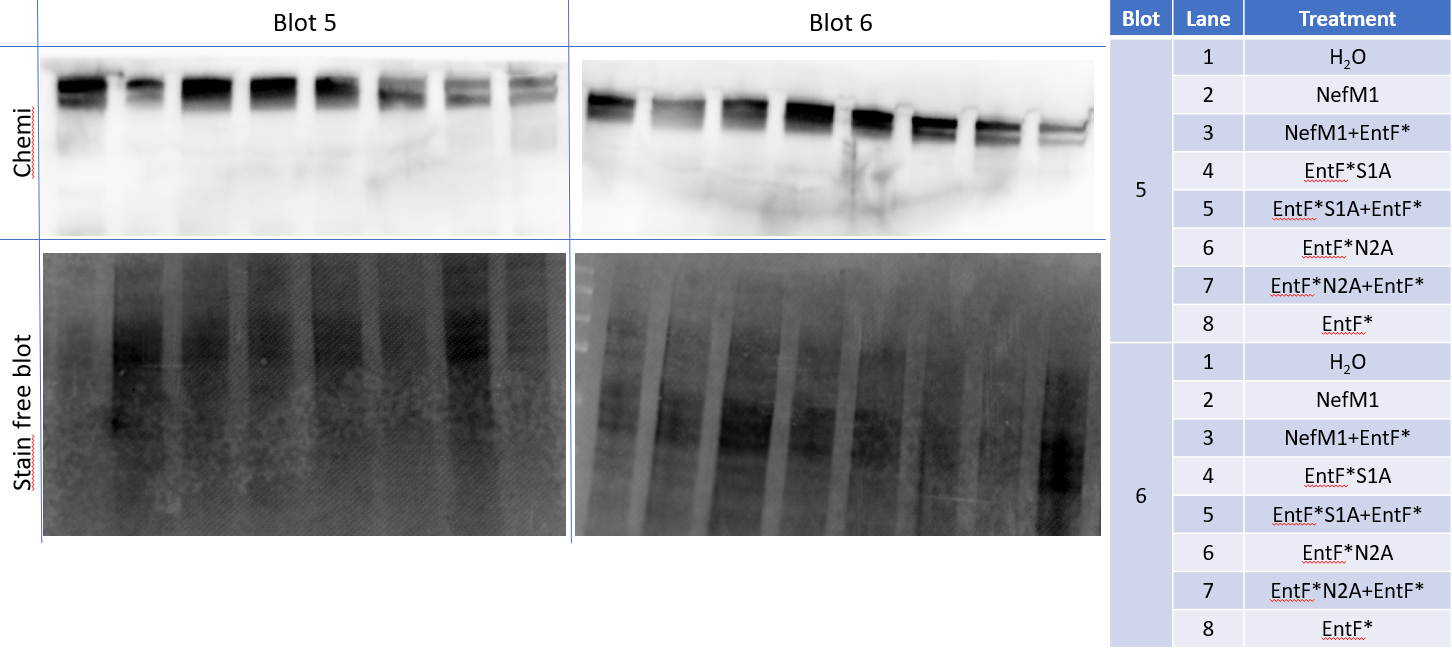

Supplement: Supplementary file 13 — Additional file 13. Raw data files Western Blot antagonist study. [file 12915_2022_1317_MOESM13_ESM.zip › Antagonist/blot5and6.PNG]

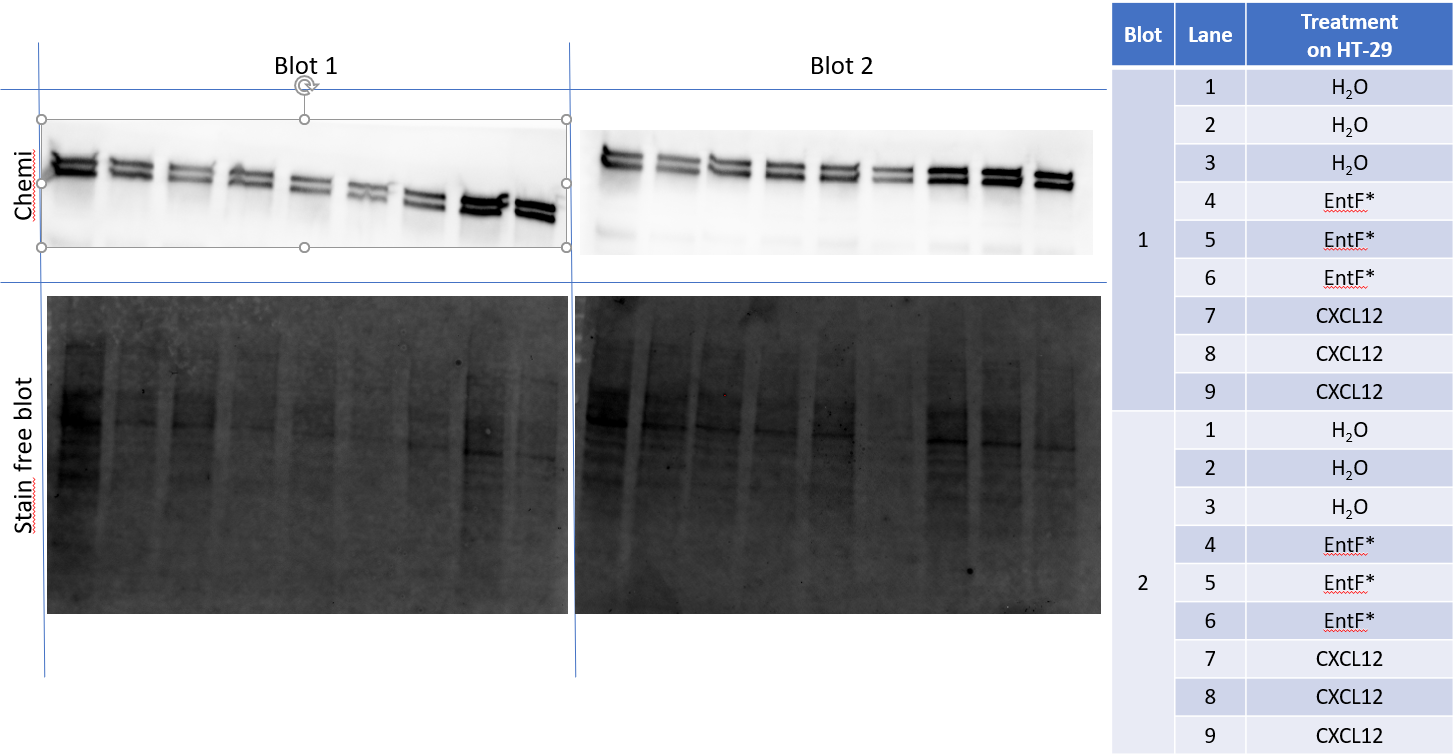

Supplement: Supplementary file 14 — Additional file 14. Raw data files Western Blot cell lines. [file 12915_2022_1317_MOESM14_ESM.zip › Othercells/blot1and2.PNG]

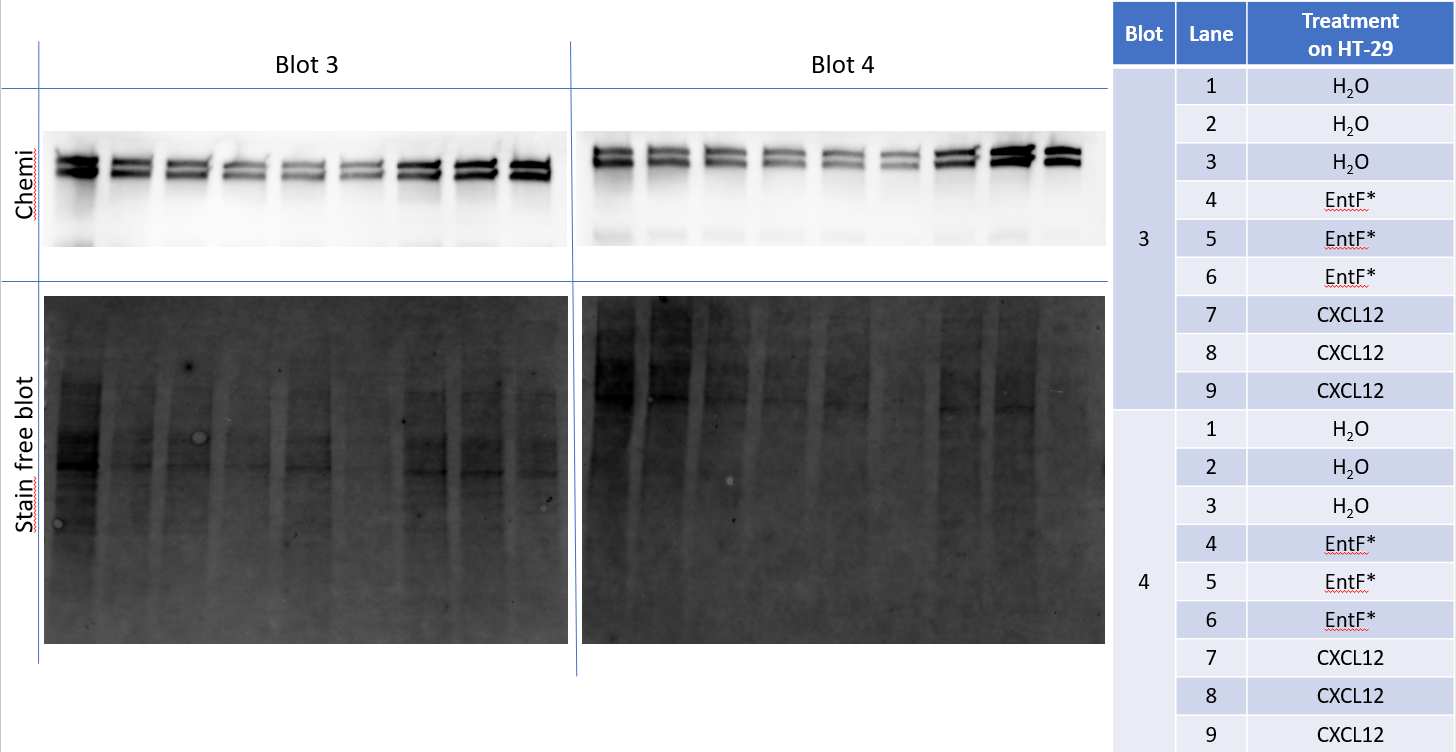

Supplement: Supplementary file 14 — Additional file 14. Raw data files Western Blot cell lines. [file 12915_2022_1317_MOESM14_ESM.zip › Othercells/blot3and4.PNG]

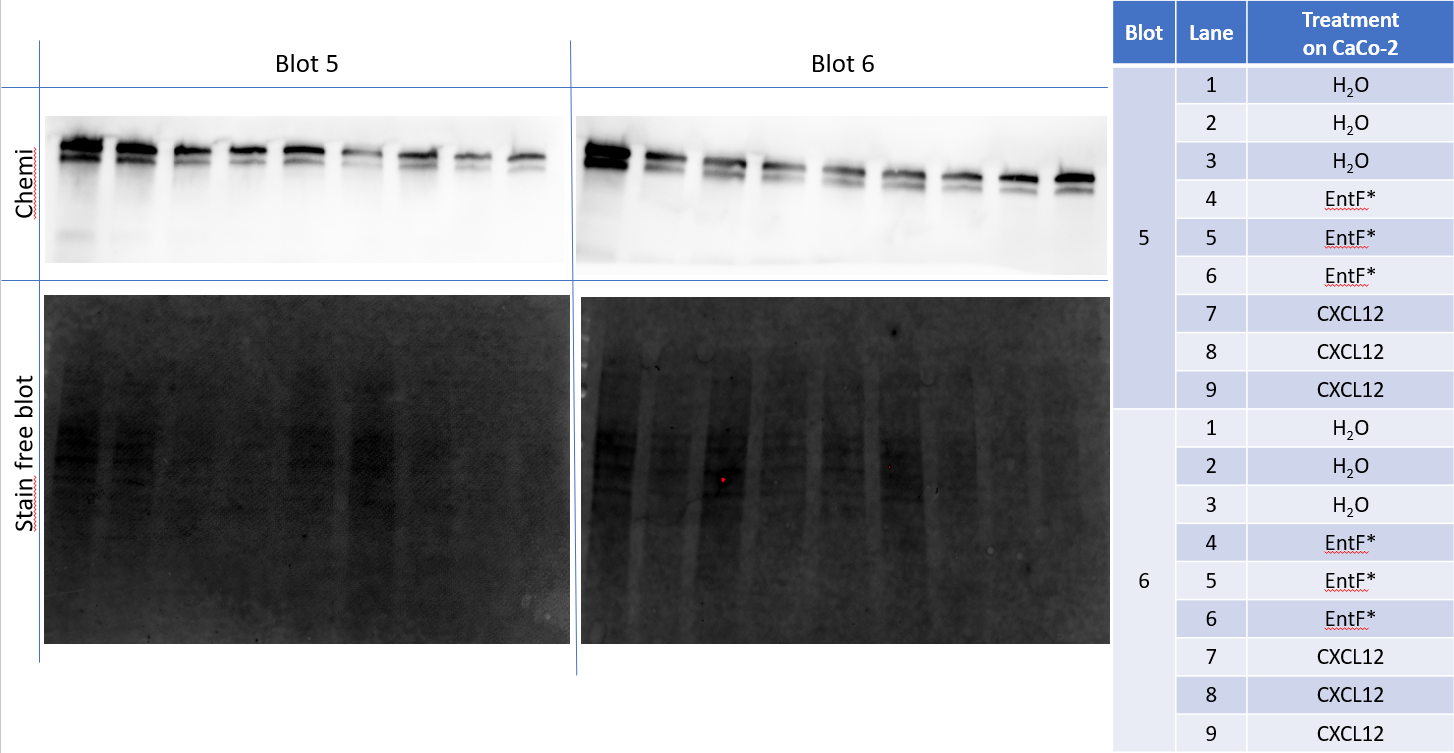

Supplement: Supplementary file 14 — Additional file 14. Raw data files Western Blot cell lines. [file 12915_2022_1317_MOESM14_ESM.zip › Othercells/blot5and6.PNG]

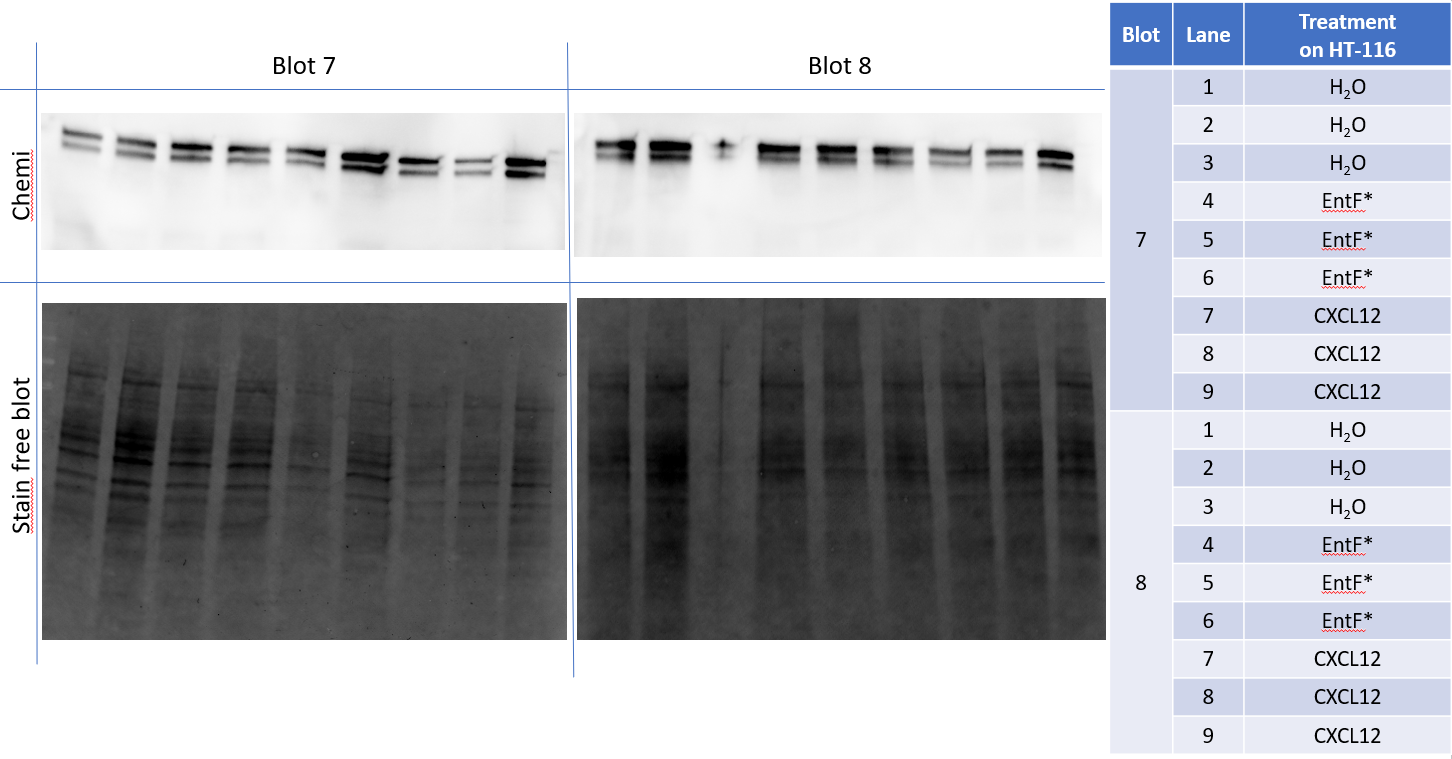

Supplement: Supplementary file 14 — Additional file 14. Raw data files Western Blot cell lines. [file 12915_2022_1317_MOESM14_ESM.zip › Othercells/blot7and8.PNG]

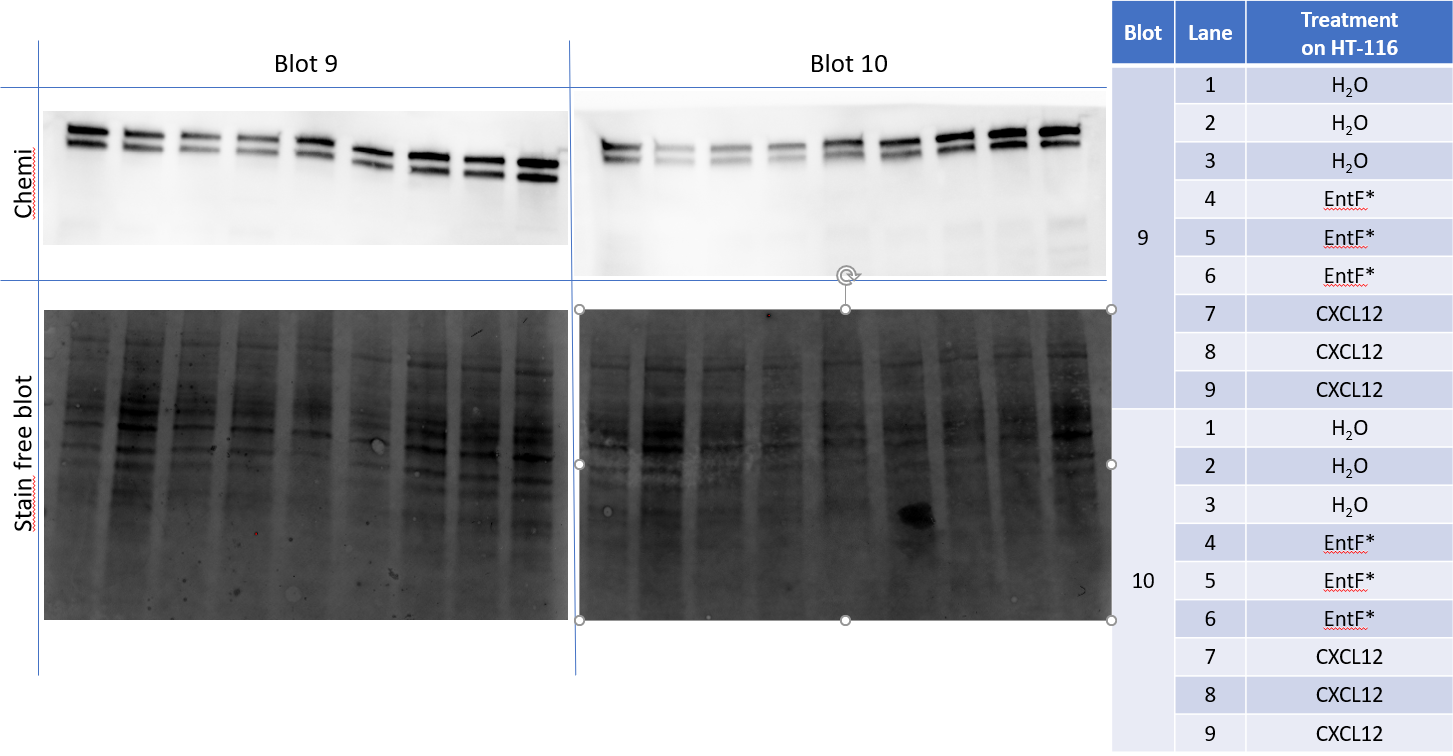

Supplement: Supplementary file 14 — Additional file 14. Raw data files Western Blot cell lines. [file 12915_2022_1317_MOESM14_ESM.zip › Othercells/blot9and10.PNG]

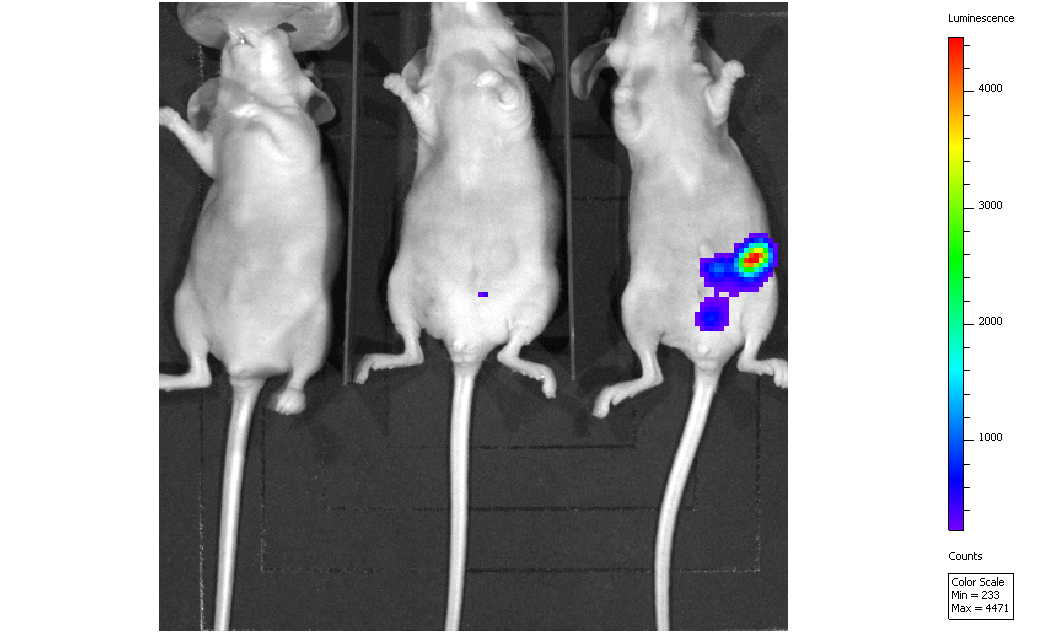

Supplement: Supplementary file 16 — Additional file 16. Raw data bioluminescence files. [file 12915_2022_1317_MOESM16_ESM.zip › Bioluminescence/Raw_Data/20180313/EW20180313113935/EW20180313113935.PNG]

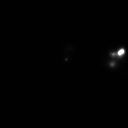

Supplement: Supplementary file 16 — Additional file 16. Raw data bioluminescence files. [file 12915_2022_1317_MOESM16_ESM.zip › Bioluminescence/Raw_Data/20180313/EW20180313113935/luminescent.TIF]

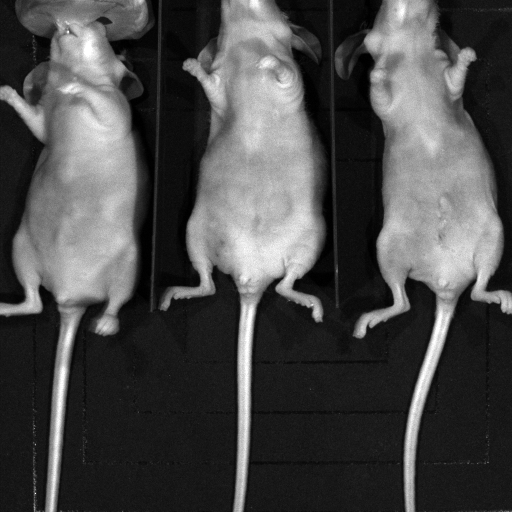

Supplement: Supplementary file 16 — Additional file 16. Raw data bioluminescence files. [file 12915_2022_1317_MOESM16_ESM.zip › Bioluminescence/Raw_Data/20180313/EW20180313113935/photograph.TIF]

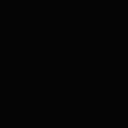

Supplement: Supplementary file 16 — Additional file 16. Raw data bioluminescence files. [file 12915_2022_1317_MOESM16_ESM.zip › Bioluminescence/Raw_Data/20180313/EW20180313113935/readbiasonly.TIF]

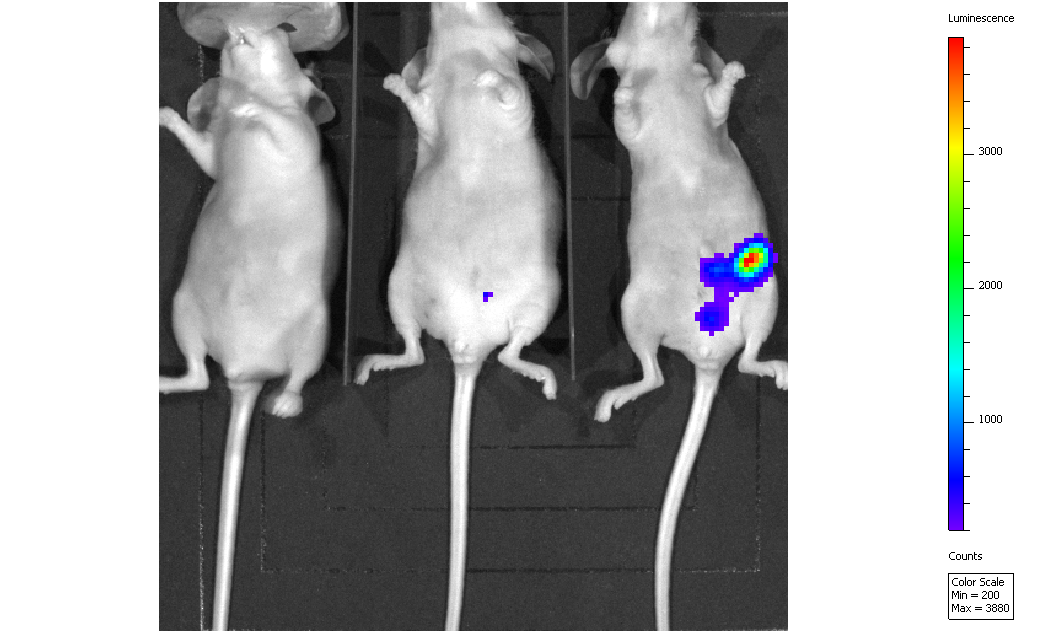

Supplement: Supplementary file 16 — Additional file 16. Raw data bioluminescence files. [file 12915_2022_1317_MOESM16_ESM.zip › Bioluminescence/Raw_Data/20180313/EW20180313114219/EW20180313114219.PNG]

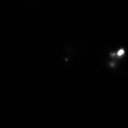

Supplement: Supplementary file 16 — Additional file 16. Raw data bioluminescence files. [file 12915_2022_1317_MOESM16_ESM.zip › Bioluminescence/Raw_Data/20180313/EW20180313114219/luminescent.TIF]

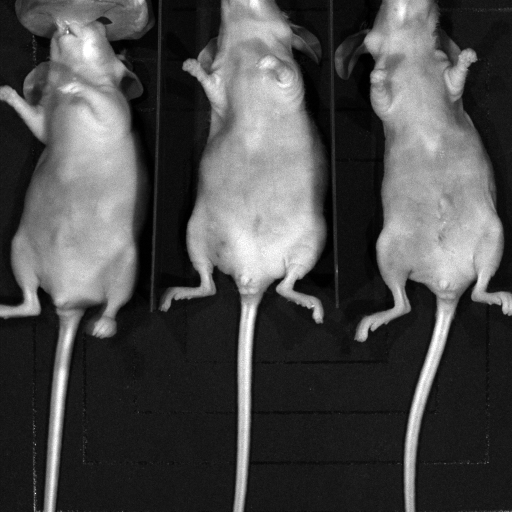

Supplement: Supplementary file 16 — Additional file 16. Raw data bioluminescence files. [file 12915_2022_1317_MOESM16_ESM.zip › Bioluminescence/Raw_Data/20180313/EW20180313114219/photograph.TIF]

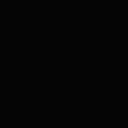

Supplement: Supplementary file 16 — Additional file 16. Raw data bioluminescence files. [file 12915_2022_1317_MOESM16_ESM.zip › Bioluminescence/Raw_Data/20180313/EW20180313114219/readbiasonly.TIF]

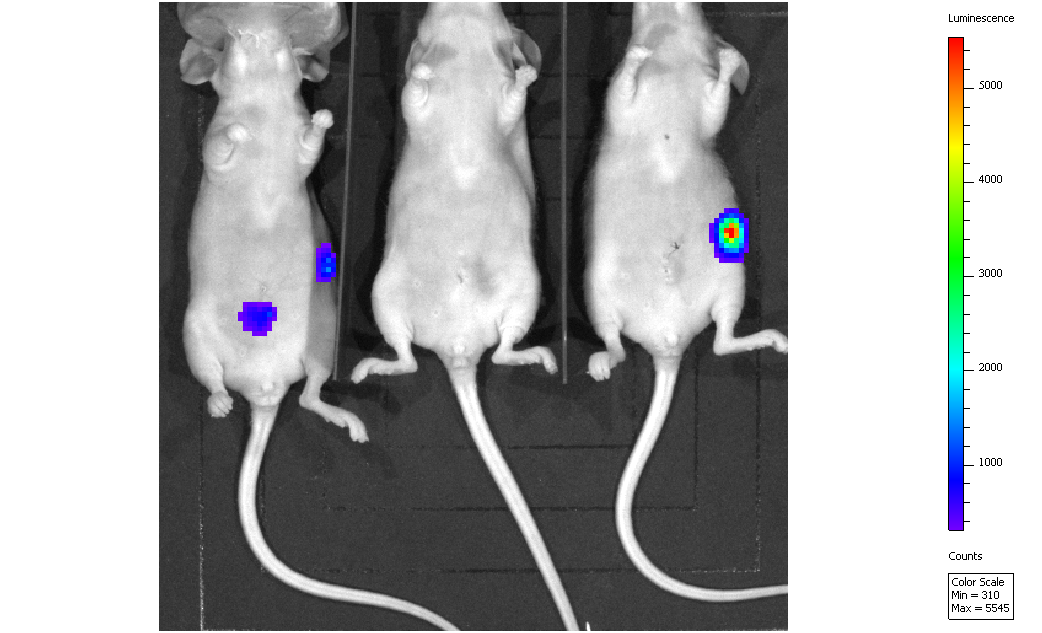

Supplement: Supplementary file 16 — Additional file 16. Raw data bioluminescence files. [file 12915_2022_1317_MOESM16_ESM.zip › Bioluminescence/Raw_Data/20180313/EW20180313115548/EW20180313115548.PNG]

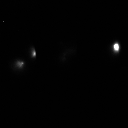

Supplement: Supplementary file 16 — Additional file 16. Raw data bioluminescence files. [file 12915_2022_1317_MOESM16_ESM.zip › Bioluminescence/Raw_Data/20180313/EW20180313115548/luminescent.TIF]

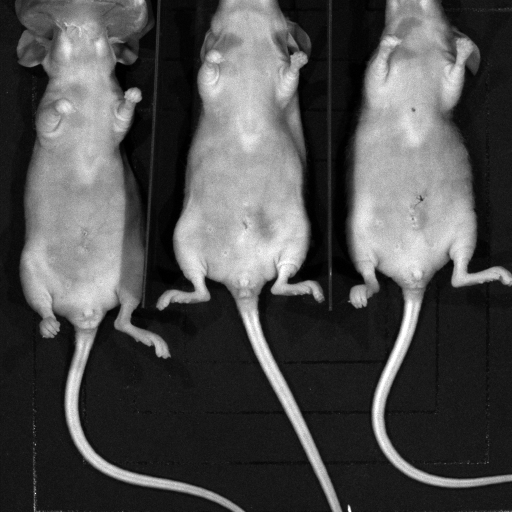

Supplement: Supplementary file 16 — Additional file 16. Raw data bioluminescence files. [file 12915_2022_1317_MOESM16_ESM.zip › Bioluminescence/Raw_Data/20180313/EW20180313115548/photograph.TIF]

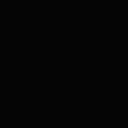

Supplement: Supplementary file 16 — Additional file 16. Raw data bioluminescence files. [file 12915_2022_1317_MOESM16_ESM.zip › Bioluminescence/Raw_Data/20180313/EW20180313115548/readbiasonly.TIF]

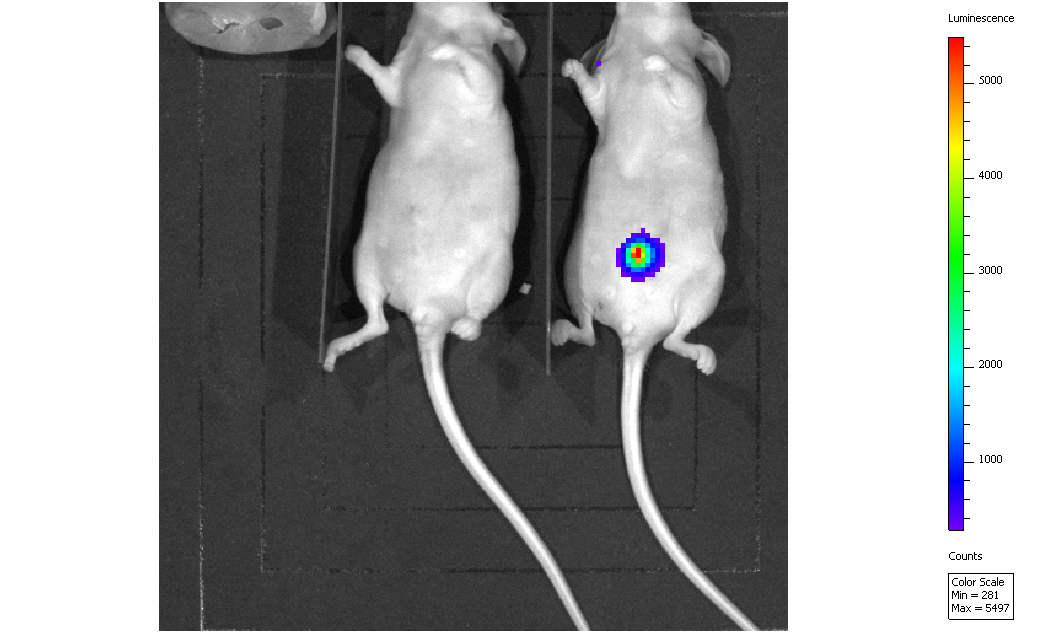

Supplement: Supplementary file 16 — Additional file 16. Raw data bioluminescence files. [file 12915_2022_1317_MOESM16_ESM.zip › Bioluminescence/Raw_Data/20180313/EW20180313120739/EW20180313120739.PNG]

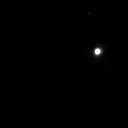

Supplement: Supplementary file 16 — Additional file 16. Raw data bioluminescence files. [file 12915_2022_1317_MOESM16_ESM.zip › Bioluminescence/Raw_Data/20180313/EW20180313120739/luminescent.TIF]

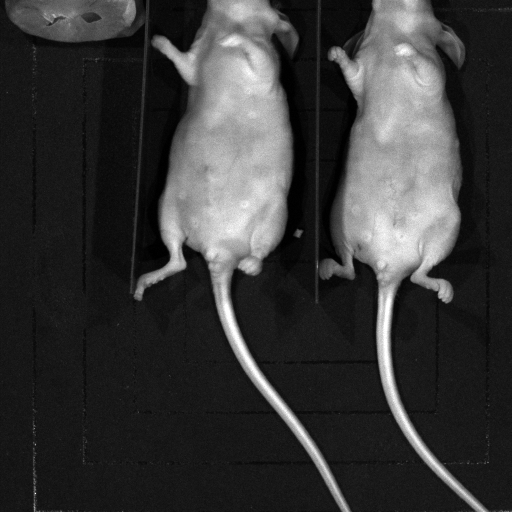

Supplement: Supplementary file 16 — Additional file 16. Raw data bioluminescence files. [file 12915_2022_1317_MOESM16_ESM.zip › Bioluminescence/Raw_Data/20180313/EW20180313120739/photograph.TIF]

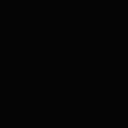

Supplement: Supplementary file 16 — Additional file 16. Raw data bioluminescence files. [file 12915_2022_1317_MOESM16_ESM.zip › Bioluminescence/Raw_Data/20180313/EW20180313120739/readbiasonly.TIF]

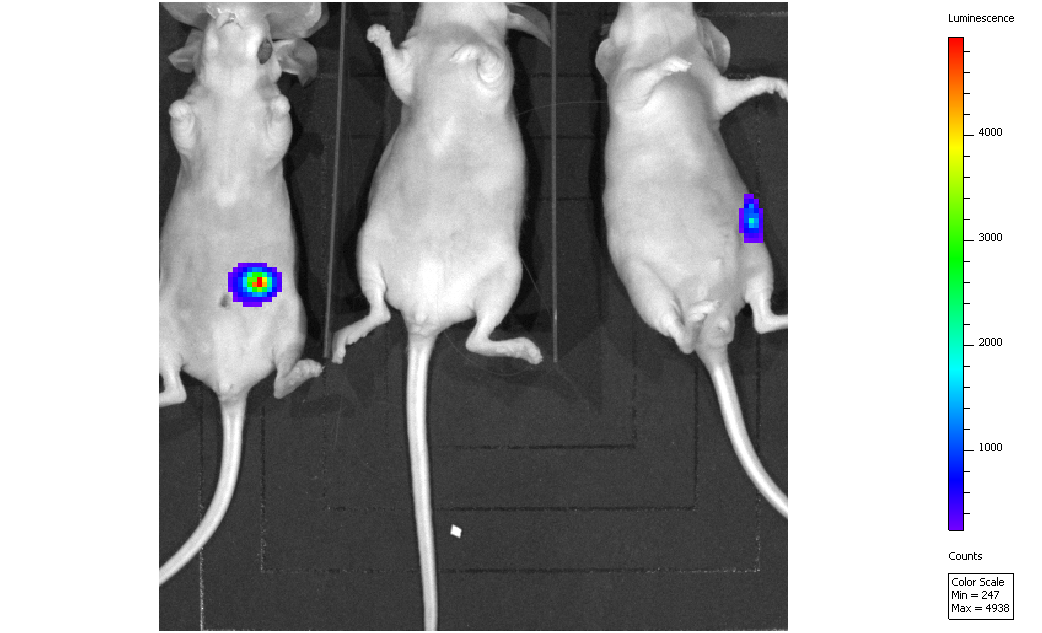

Supplement: Supplementary file 16 — Additional file 16. Raw data bioluminescence files. [file 12915_2022_1317_MOESM16_ESM.zip › Bioluminescence/Raw_Data/20180313/EW20180313121903/EW20180313121903.PNG]

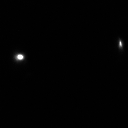

Supplement: Supplementary file 16 — Additional file 16. Raw data bioluminescence files. [file 12915_2022_1317_MOESM16_ESM.zip › Bioluminescence/Raw_Data/20180313/EW20180313121903/luminescent.TIF]

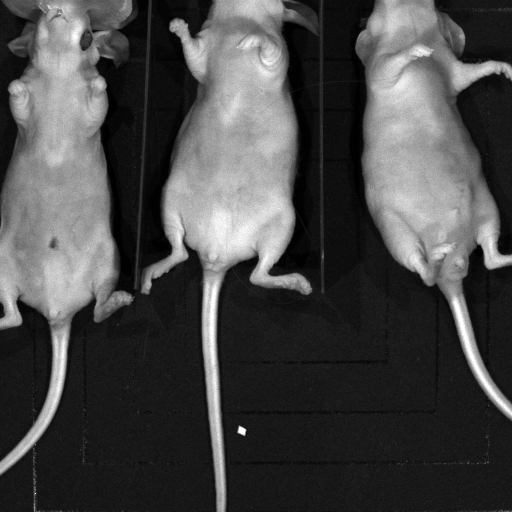

Supplement: Supplementary file 16 — Additional file 16. Raw data bioluminescence files. [file 12915_2022_1317_MOESM16_ESM.zip › Bioluminescence/Raw_Data/20180313/EW20180313121903/photograph.TIF]

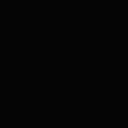

Supplement: Supplementary file 16 — Additional file 16. Raw data bioluminescence files. [file 12915_2022_1317_MOESM16_ESM.zip › Bioluminescence/Raw_Data/20180313/EW20180313121903/readbiasonly.TIF]

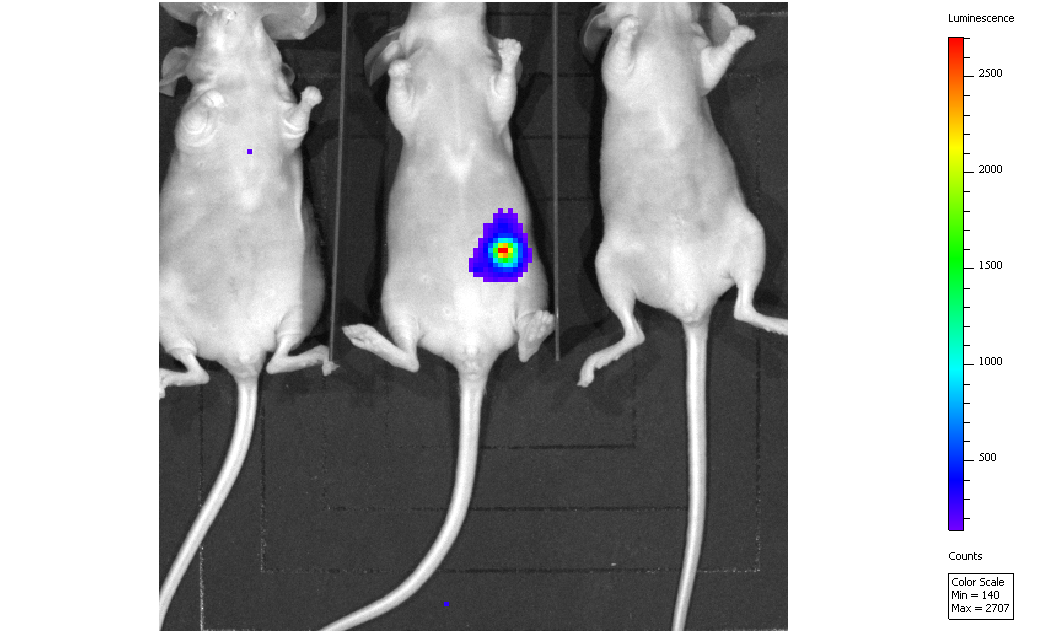

Supplement: Supplementary file 16 — Additional file 16. Raw data bioluminescence files. [file 12915_2022_1317_MOESM16_ESM.zip › Bioluminescence/Raw_Data/20180313/EW20180313122756/EW20180313122756.PNG]

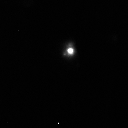

Supplement: Supplementary file 16 — Additional file 16. Raw data bioluminescence files. [file 12915_2022_1317_MOESM16_ESM.zip › Bioluminescence/Raw_Data/20180313/EW20180313122756/luminescent.TIF]

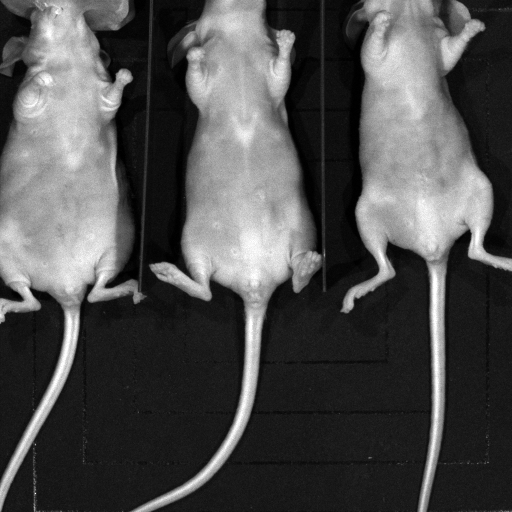

Supplement: Supplementary file 16 — Additional file 16. Raw data bioluminescence files. [file 12915_2022_1317_MOESM16_ESM.zip › Bioluminescence/Raw_Data/20180313/EW20180313122756/photograph.TIF]

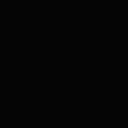

Supplement: Supplementary file 16 — Additional file 16. Raw data bioluminescence files. [file 12915_2022_1317_MOESM16_ESM.zip › Bioluminescence/Raw_Data/20180313/EW20180313122756/readbiasonly.TIF]

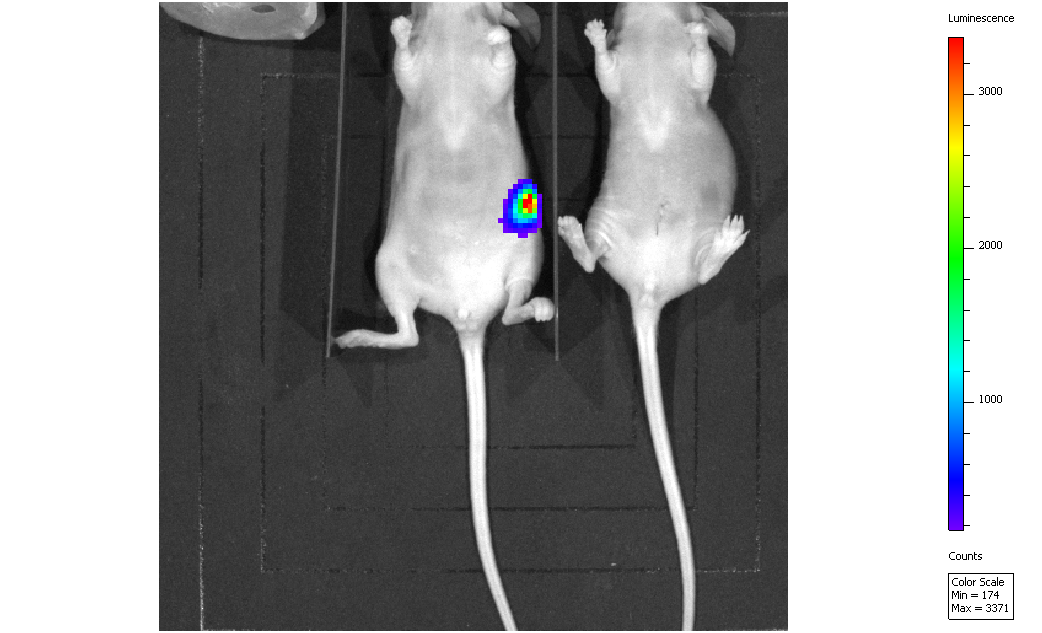

Supplement: Supplementary file 16 — Additional file 16. Raw data bioluminescence files. [file 12915_2022_1317_MOESM16_ESM.zip › Bioluminescence/Raw_Data/20180313/EW20180313123609/EW20180313123609.PNG]

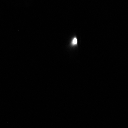

Supplement: Supplementary file 16 — Additional file 16. Raw data bioluminescence files. [file 12915_2022_1317_MOESM16_ESM.zip › Bioluminescence/Raw_Data/20180313/EW20180313123609/luminescent.TIF]

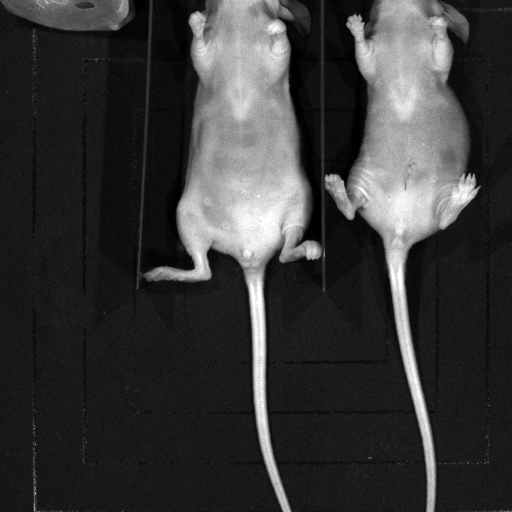

Supplement: Supplementary file 16 — Additional file 16. Raw data bioluminescence files. [file 12915_2022_1317_MOESM16_ESM.zip › Bioluminescence/Raw_Data/20180313/EW20180313123609/photograph.TIF]

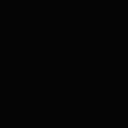

Supplement: Supplementary file 16 — Additional file 16. Raw data bioluminescence files. [file 12915_2022_1317_MOESM16_ESM.zip › Bioluminescence/Raw_Data/20180313/EW20180313123609/readbiasonly.TIF]

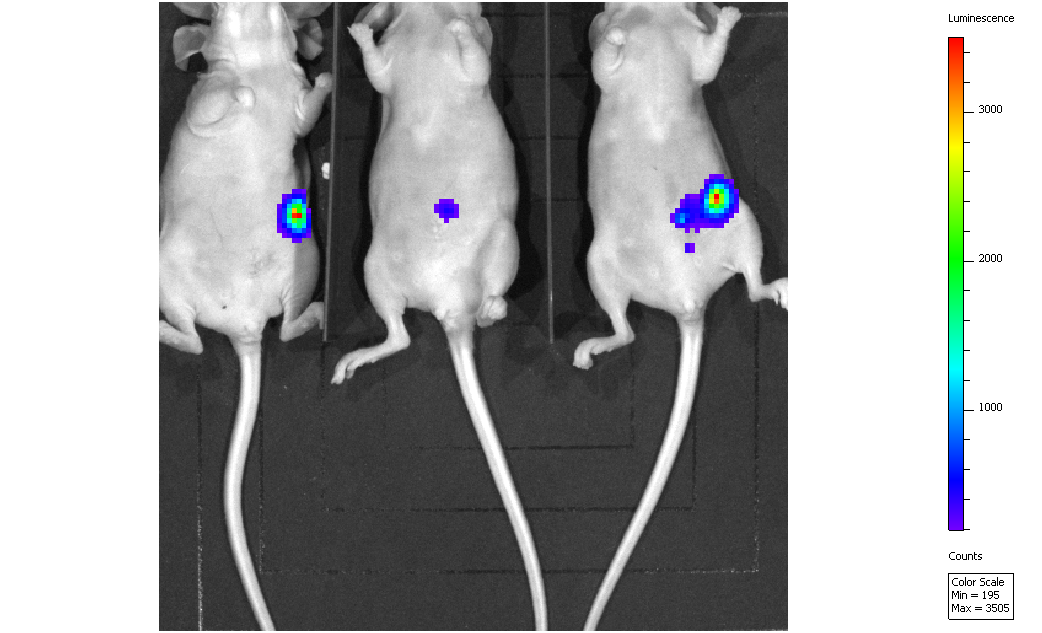

Supplement: Supplementary file 16 — Additional file 16. Raw data bioluminescence files. [file 12915_2022_1317_MOESM16_ESM.zip › Bioluminescence/Raw_Data/20180313/EW20180313124956/EW20180313124956.PNG]

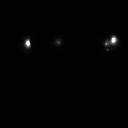

Supplement: Supplementary file 16 — Additional file 16. Raw data bioluminescence files. [file 12915_2022_1317_MOESM16_ESM.zip › Bioluminescence/Raw_Data/20180313/EW20180313124956/luminescent.TIF]

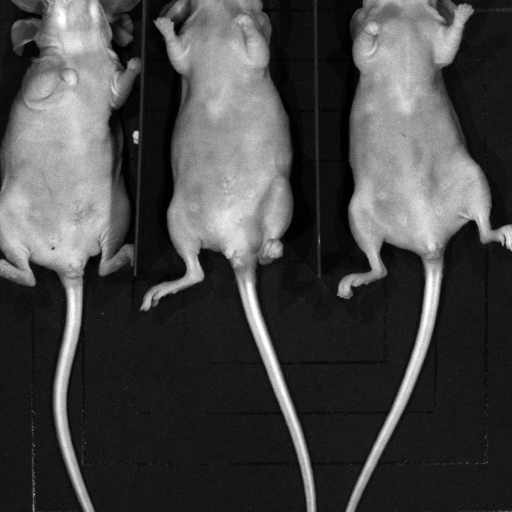

Supplement: Supplementary file 16 — Additional file 16. Raw data bioluminescence files. [file 12915_2022_1317_MOESM16_ESM.zip › Bioluminescence/Raw_Data/20180313/EW20180313124956/photograph.TIF]

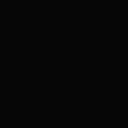

Supplement: Supplementary file 16 — Additional file 16. Raw data bioluminescence files. [file 12915_2022_1317_MOESM16_ESM.zip › Bioluminescence/Raw_Data/20180313/EW20180313124956/readbiasonly.TIF]

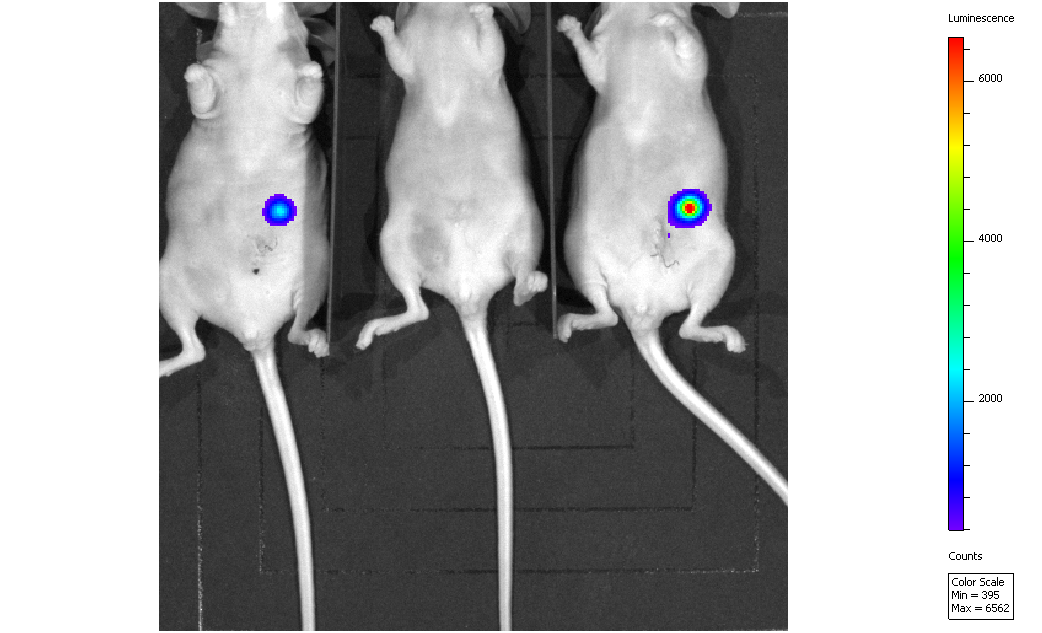

Supplement: Supplementary file 16 — Additional file 16. Raw data bioluminescence files. [file 12915_2022_1317_MOESM16_ESM.zip › Bioluminescence/Raw_Data/20180313/EW20180313125812/EW20180313125812.PNG]

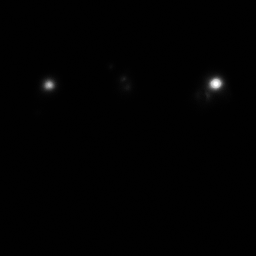

Supplement: Supplementary file 16 — Additional file 16. Raw data bioluminescence files. [file 12915_2022_1317_MOESM16_ESM.zip › Bioluminescence/Raw_Data/20180313/EW20180313125812/luminescent.TIF]

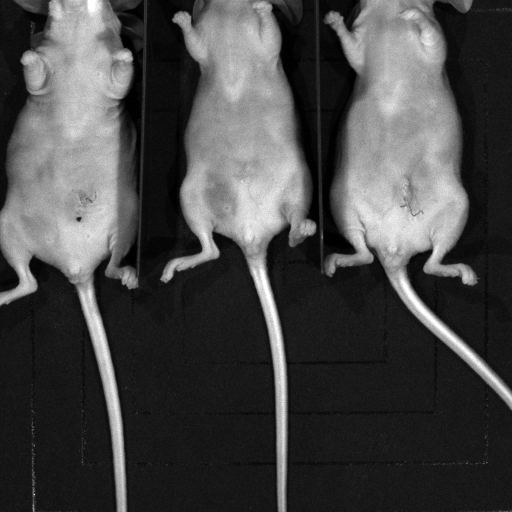

Supplement: Supplementary file 16 — Additional file 16. Raw data bioluminescence files. [file 12915_2022_1317_MOESM16_ESM.zip › Bioluminescence/Raw_Data/20180313/EW20180313125812/photograph.TIF]

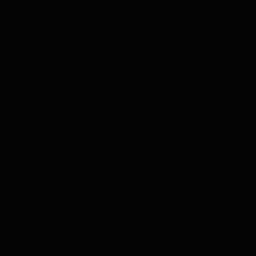

Supplement: Supplementary file 16 — Additional file 16. Raw data bioluminescence files. [file 12915_2022_1317_MOESM16_ESM.zip › Bioluminescence/Raw_Data/20180313/EW20180313125812/readbiasonly.TIF]

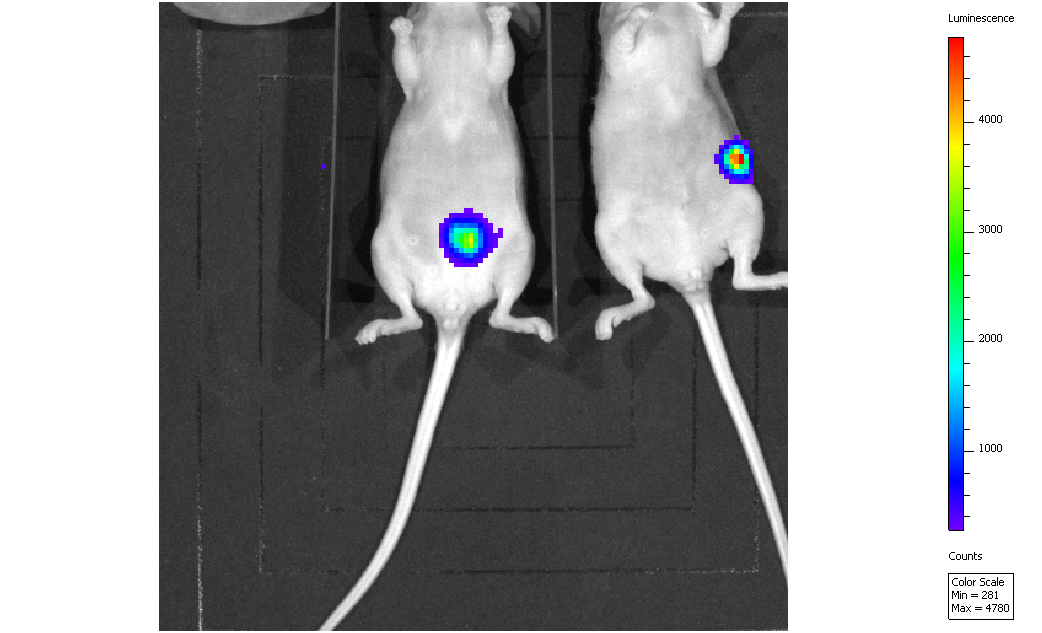

Supplement: Supplementary file 16 — Additional file 16. Raw data bioluminescence files. [file 12915_2022_1317_MOESM16_ESM.zip › Bioluminescence/Raw_Data/20180313/EW20180313130543/EW20180313130543.PNG]

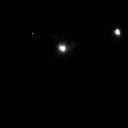

Supplement: Supplementary file 16 — Additional file 16. Raw data bioluminescence files. [file 12915_2022_1317_MOESM16_ESM.zip › Bioluminescence/Raw_Data/20180313/EW20180313130543/luminescent.TIF]

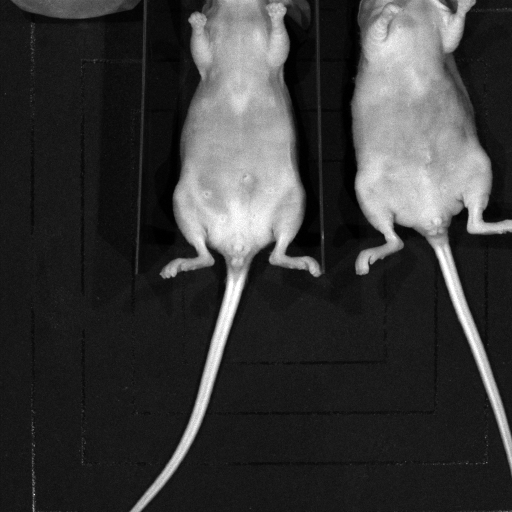

Supplement: Supplementary file 16 — Additional file 16. Raw data bioluminescence files. [file 12915_2022_1317_MOESM16_ESM.zip › Bioluminescence/Raw_Data/20180313/EW20180313130543/photograph.TIF]

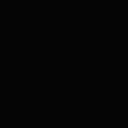

Supplement: Supplementary file 16 — Additional file 16. Raw data bioluminescence files. [file 12915_2022_1317_MOESM16_ESM.zip › Bioluminescence/Raw_Data/20180313/EW20180313130543/readbiasonly.TIF]

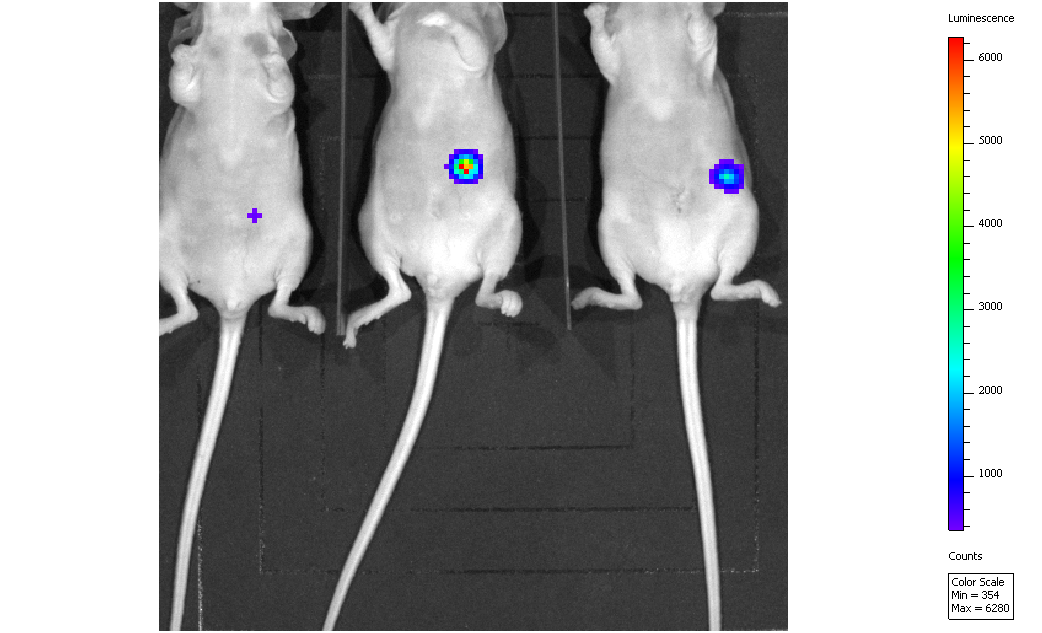

Supplement: Supplementary file 16 — Additional file 16. Raw data bioluminescence files. [file 12915_2022_1317_MOESM16_ESM.zip › Bioluminescence/Raw_Data/20180313/EW20180313131329/EW20180313131329.PNG]

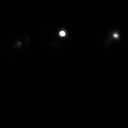

Supplement: Supplementary file 16 — Additional file 16. Raw data bioluminescence files. [file 12915_2022_1317_MOESM16_ESM.zip › Bioluminescence/Raw_Data/20180313/EW20180313131329/luminescent.TIF]

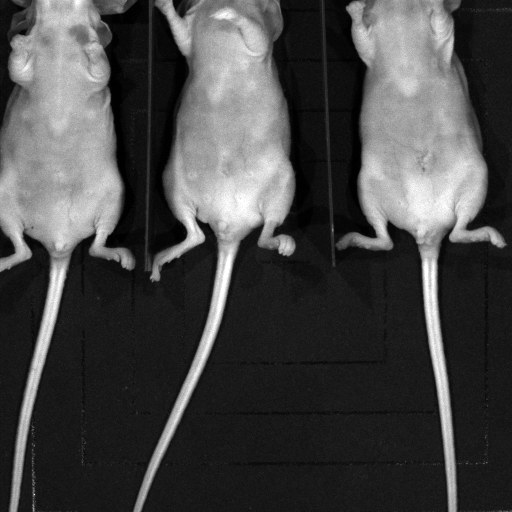

Supplement: Supplementary file 16 — Additional file 16. Raw data bioluminescence files. [file 12915_2022_1317_MOESM16_ESM.zip › Bioluminescence/Raw_Data/20180313/EW20180313131329/photograph.TIF]

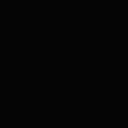

Supplement: Supplementary file 16 — Additional file 16. Raw data bioluminescence files. [file 12915_2022_1317_MOESM16_ESM.zip › Bioluminescence/Raw_Data/20180313/EW20180313131329/readbiasonly.TIF]

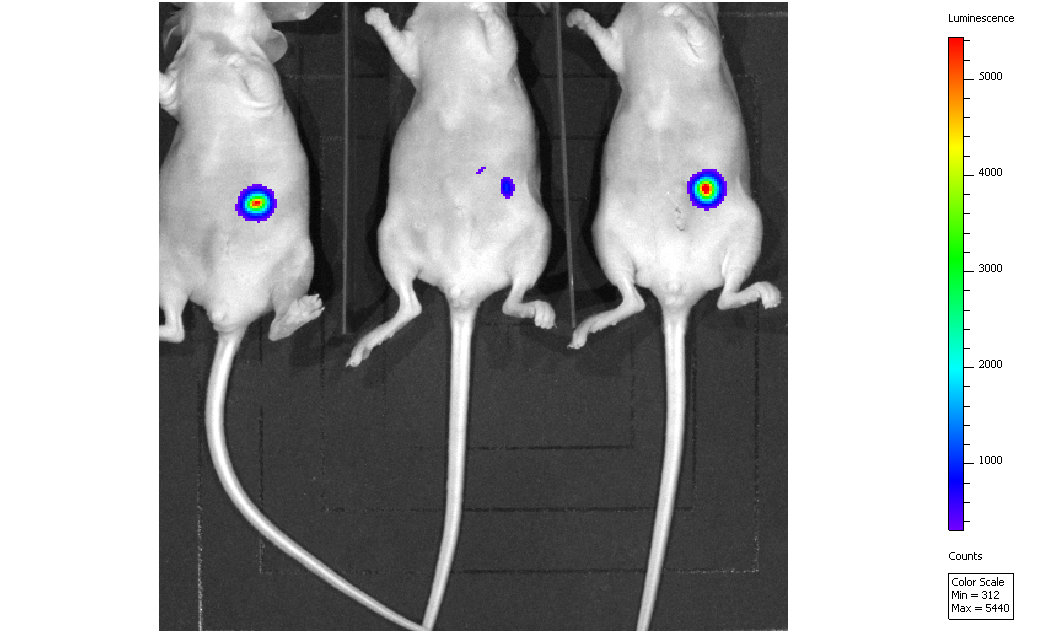

Supplement: Supplementary file 16 — Additional file 16. Raw data bioluminescence files. [file 12915_2022_1317_MOESM16_ESM.zip › Bioluminescence/Raw_Data/20180313/EW20180313132032/EW20180313132032.PNG]

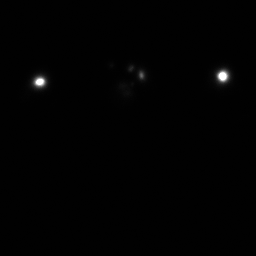

Supplement: Supplementary file 16 — Additional file 16. Raw data bioluminescence files. [file 12915_2022_1317_MOESM16_ESM.zip › Bioluminescence/Raw_Data/20180313/EW20180313132032/luminescent.TIF]

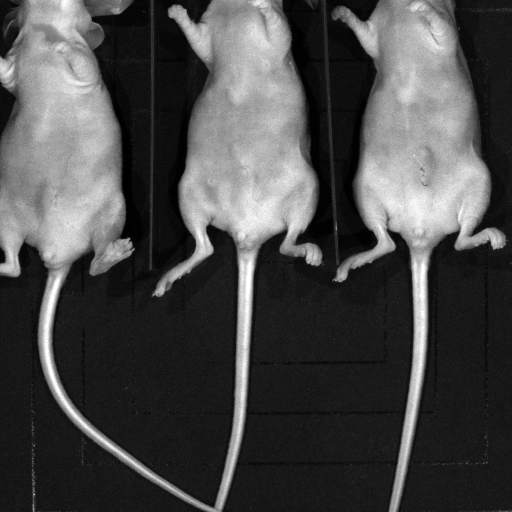

Supplement: Supplementary file 16 — Additional file 16. Raw data bioluminescence files. [file 12915_2022_1317_MOESM16_ESM.zip › Bioluminescence/Raw_Data/20180313/EW20180313132032/photograph.TIF]

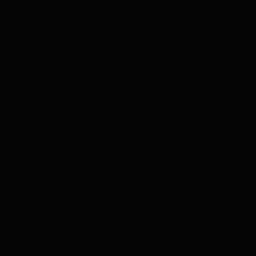

Supplement: Supplementary file 16 — Additional file 16. Raw data bioluminescence files. [file 12915_2022_1317_MOESM16_ESM.zip › Bioluminescence/Raw_Data/20180313/EW20180313132032/readbiasonly.TIF]

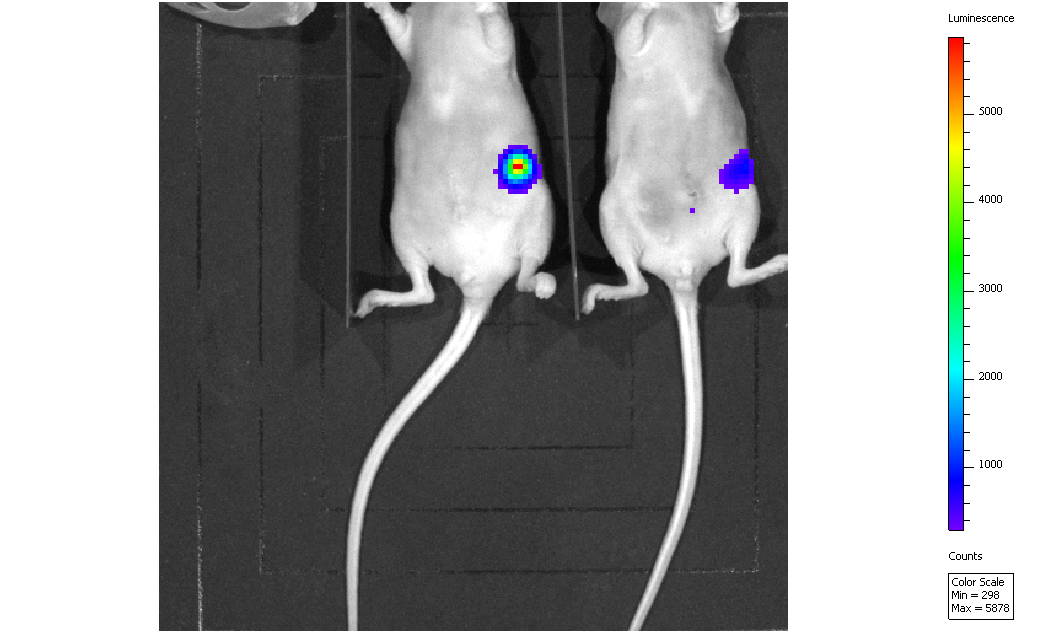

Supplement: Supplementary file 16 — Additional file 16. Raw data bioluminescence files. [file 12915_2022_1317_MOESM16_ESM.zip › Bioluminescence/Raw_Data/20180313/EW20180313132815/EW20180313132815.PNG]

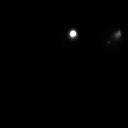

Supplement: Supplementary file 16 — Additional file 16. Raw data bioluminescence files. [file 12915_2022_1317_MOESM16_ESM.zip › Bioluminescence/Raw_Data/20180313/EW20180313132815/luminescent.TIF]

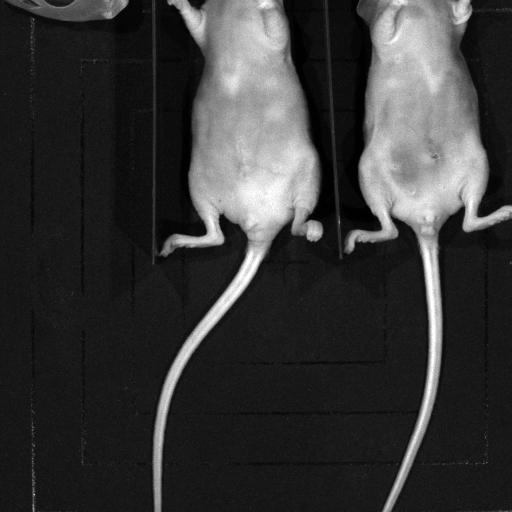

Supplement: Supplementary file 16 — Additional file 16. Raw data bioluminescence files. [file 12915_2022_1317_MOESM16_ESM.zip › Bioluminescence/Raw_Data/20180313/EW20180313132815/photograph.TIF]

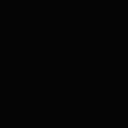

Supplement: Supplementary file 16 — Additional file 16. Raw data bioluminescence files. [file 12915_2022_1317_MOESM16_ESM.zip › Bioluminescence/Raw_Data/20180313/EW20180313132815/readbiasonly.TIF]

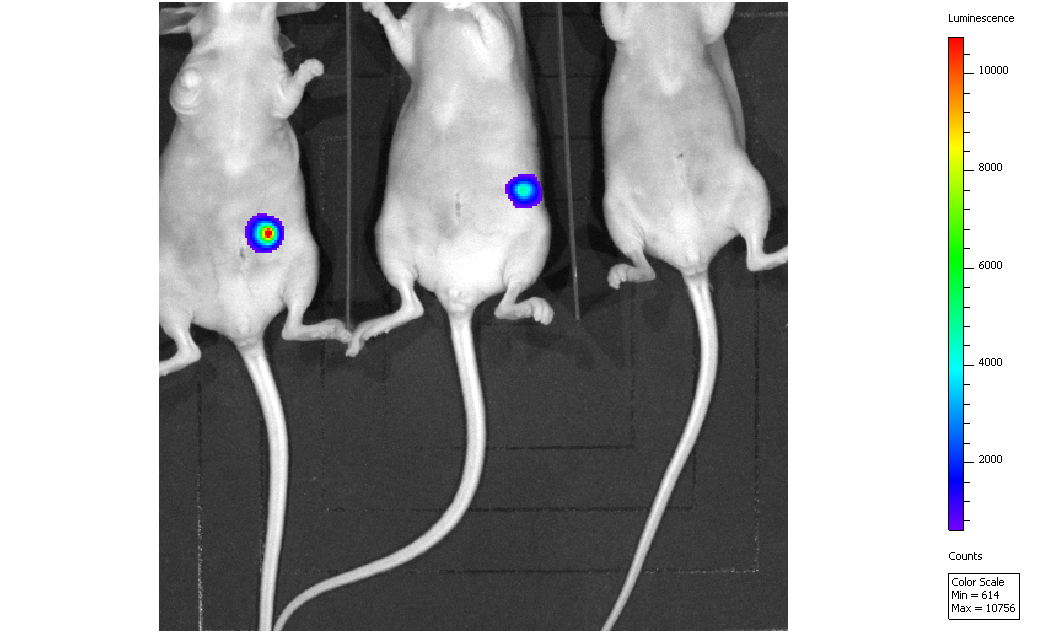

Supplement: Supplementary file 16 — Additional file 16. Raw data bioluminescence files. [file 12915_2022_1317_MOESM16_ESM.zip › Bioluminescence/Raw_Data/20180313/EW20180313133451/EW20180313133451.PNG]

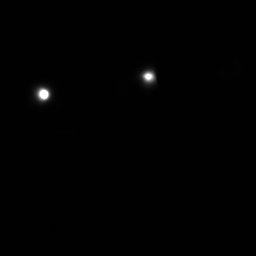

Supplement: Supplementary file 16 — Additional file 16. Raw data bioluminescence files. [file 12915_2022_1317_MOESM16_ESM.zip › Bioluminescence/Raw_Data/20180313/EW20180313133451/luminescent.TIF]

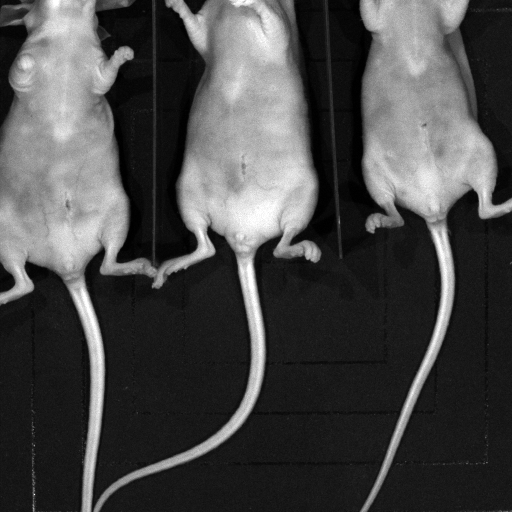

Supplement: Supplementary file 16 — Additional file 16. Raw data bioluminescence files. [file 12915_2022_1317_MOESM16_ESM.zip › Bioluminescence/Raw_Data/20180313/EW20180313133451/photograph.TIF]

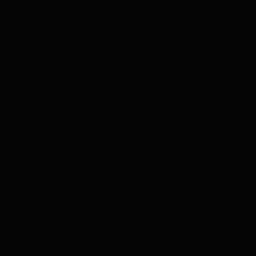

Supplement: Supplementary file 16 — Additional file 16. Raw data bioluminescence files. [file 12915_2022_1317_MOESM16_ESM.zip › Bioluminescence/Raw_Data/20180313/EW20180313133451/readbiasonly.TIF]

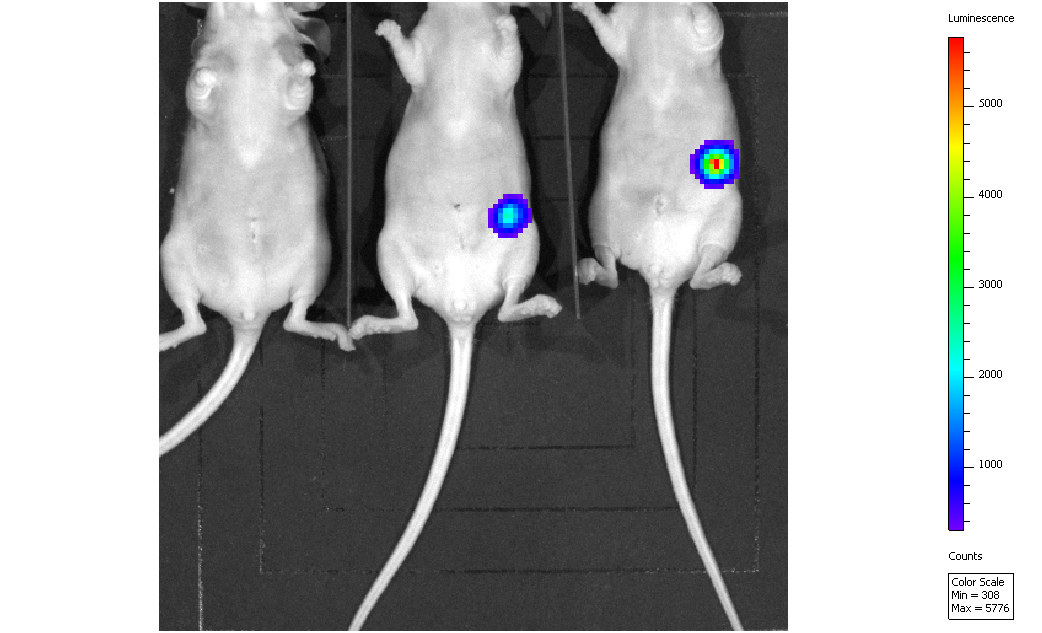

Supplement: Supplementary file 16 — Additional file 16. Raw data bioluminescence files. [file 12915_2022_1317_MOESM16_ESM.zip › Bioluminescence/Raw_Data/20180313/EW20180313134134/EW20180313134134.PNG]

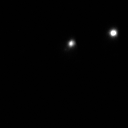

Supplement: Supplementary file 16 — Additional file 16. Raw data bioluminescence files. [file 12915_2022_1317_MOESM16_ESM.zip › Bioluminescence/Raw_Data/20180313/EW20180313134134/luminescent.TIF]

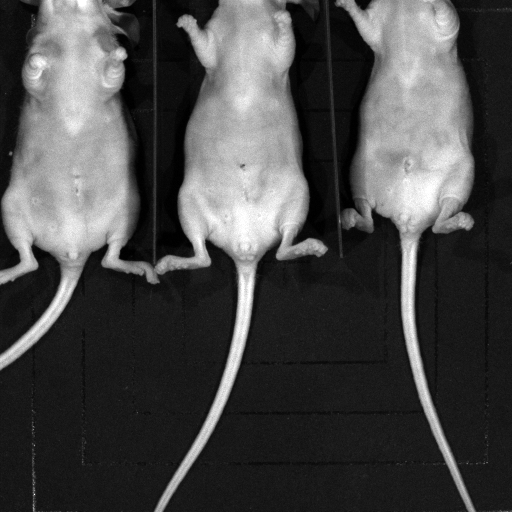

Supplement: Supplementary file 16 — Additional file 16. Raw data bioluminescence files. [file 12915_2022_1317_MOESM16_ESM.zip › Bioluminescence/Raw_Data/20180313/EW20180313134134/photograph.TIF]

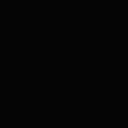

Supplement: Supplementary file 16 — Additional file 16. Raw data bioluminescence files. [file 12915_2022_1317_MOESM16_ESM.zip › Bioluminescence/Raw_Data/20180313/EW20180313134134/readbiasonly.TIF]

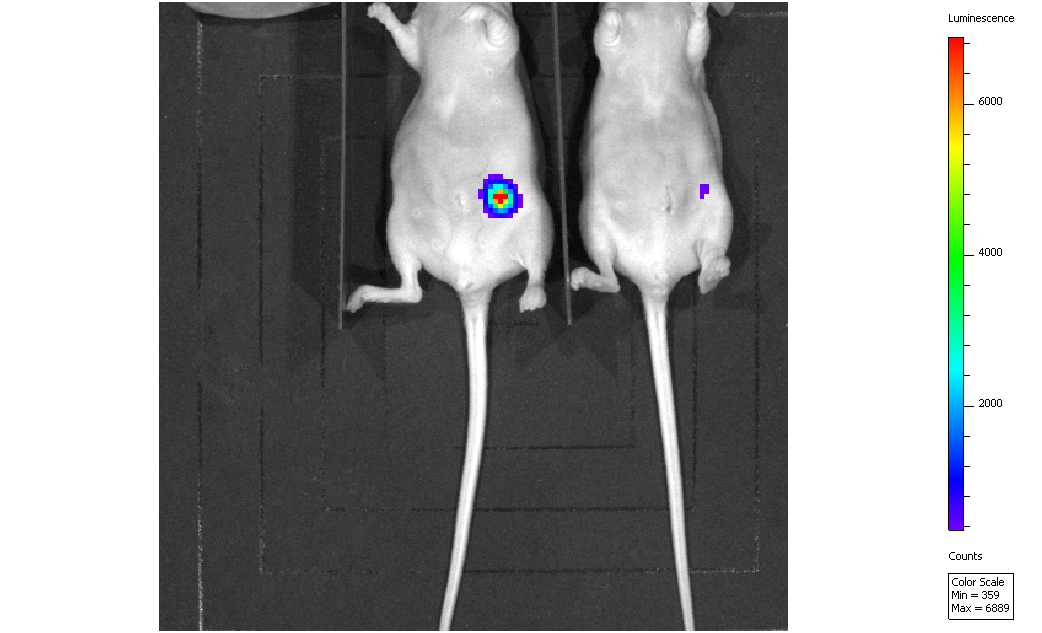

Supplement: Supplementary file 16 — Additional file 16. Raw data bioluminescence files. [file 12915_2022_1317_MOESM16_ESM.zip › Bioluminescence/Raw_Data/20180313/EW20180313134759/EW20180313134759.PNG]

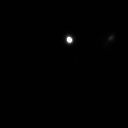

Supplement: Supplementary file 16 — Additional file 16. Raw data bioluminescence files. [file 12915_2022_1317_MOESM16_ESM.zip › Bioluminescence/Raw_Data/20180313/EW20180313134759/luminescent.TIF]

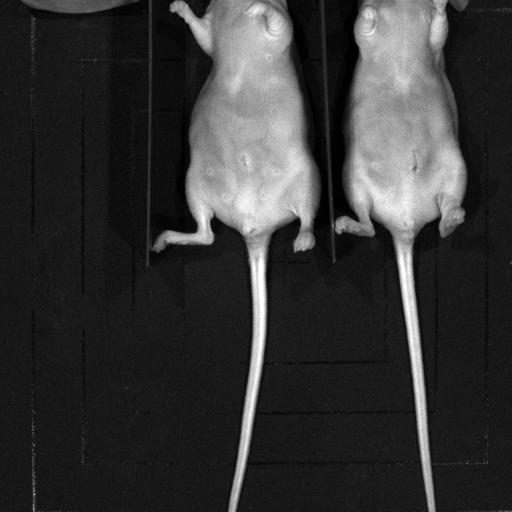

Supplement: Supplementary file 16 — Additional file 16. Raw data bioluminescence files. [file 12915_2022_1317_MOESM16_ESM.zip › Bioluminescence/Raw_Data/20180313/EW20180313134759/photograph.TIF]

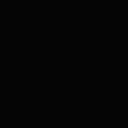

Supplement: Supplementary file 16 — Additional file 16. Raw data bioluminescence files. [file 12915_2022_1317_MOESM16_ESM.zip › Bioluminescence/Raw_Data/20180313/EW20180313134759/readbiasonly.TIF]

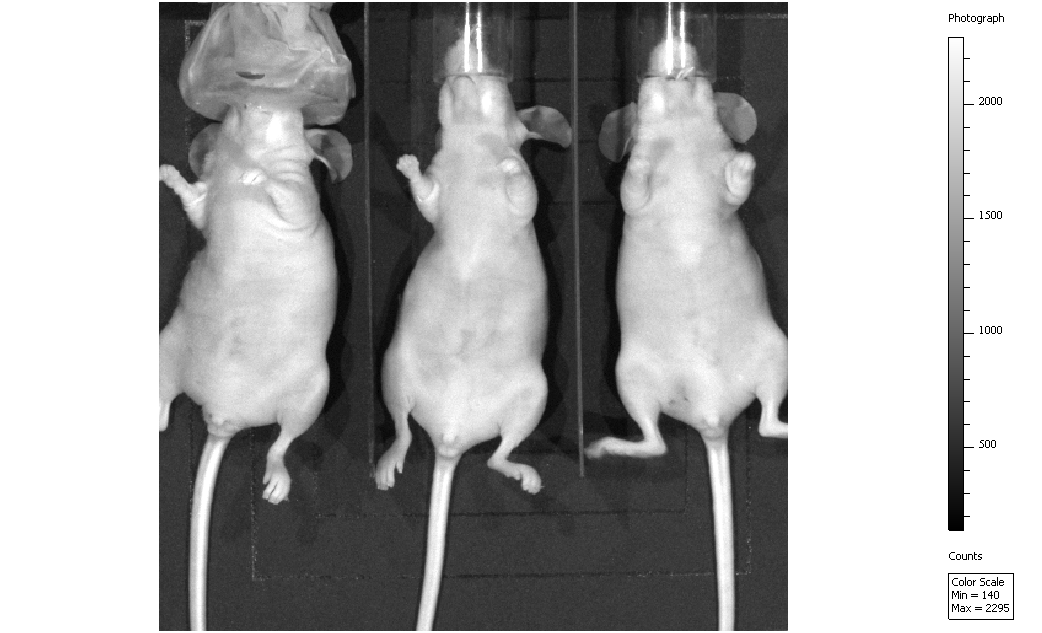

Supplement: Supplementary file 16 — Additional file 16. Raw data bioluminescence files. [file 12915_2022_1317_MOESM16_ESM.zip › Bioluminescence/Raw_Data/20180320/EW20180320111204/EW20180320111204.PNG]

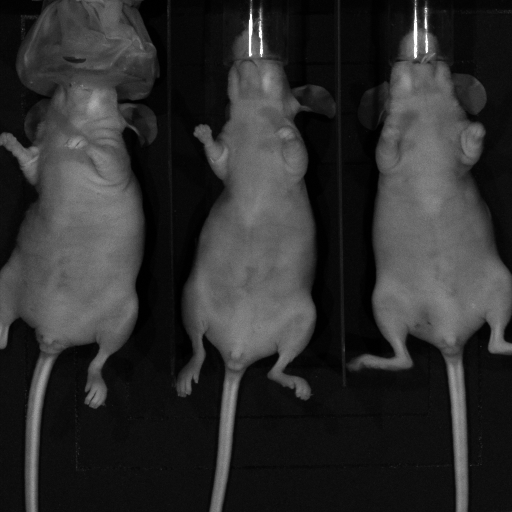

Supplement: Supplementary file 16 — Additional file 16. Raw data bioluminescence files. [file 12915_2022_1317_MOESM16_ESM.zip › Bioluminescence/Raw_Data/20180320/EW20180320111204/photograph.TIF]

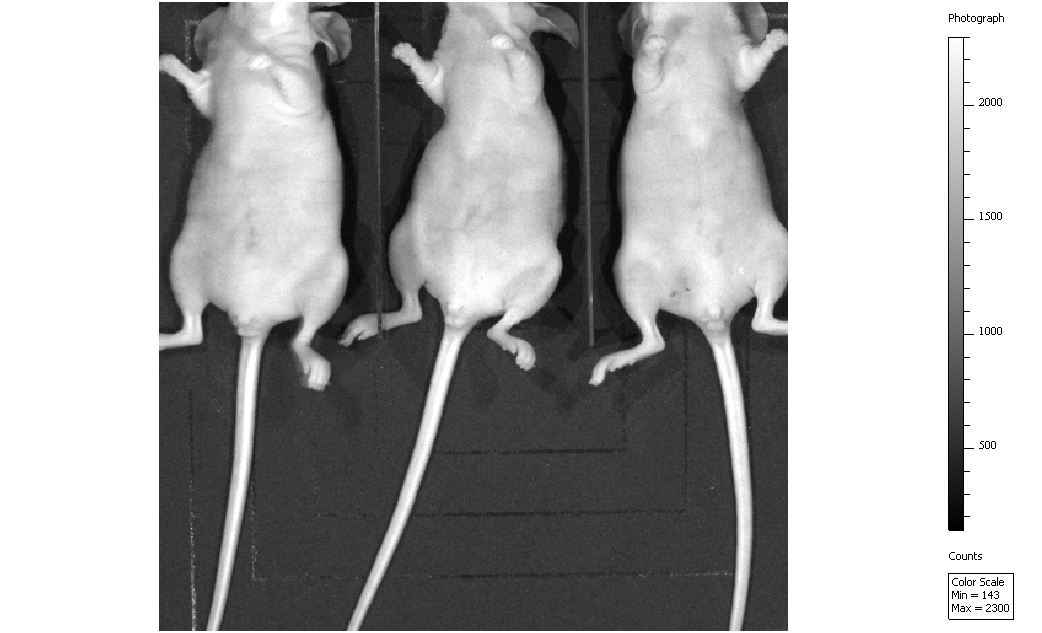

Supplement: Supplementary file 16 — Additional file 16. Raw data bioluminescence files. [file 12915_2022_1317_MOESM16_ESM.zip › Bioluminescence/Raw_Data/20180320/EW20180320111314/EW20180320111314.PNG]

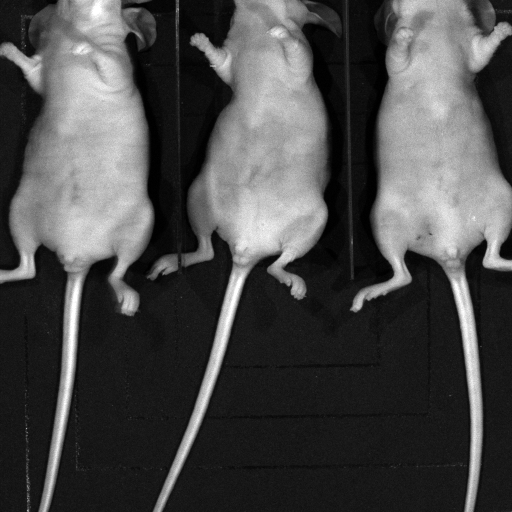

Supplement: Supplementary file 16 — Additional file 16. Raw data bioluminescence files. [file 12915_2022_1317_MOESM16_ESM.zip › Bioluminescence/Raw_Data/20180320/EW20180320111314/photograph.TIF]
