# Supplementary figures and images for: The quorum sensing peptide EntF* promotes colorectal cancer metastasis in mice: a new factor in the host-microbiome interaction (part 2 of 2)
Source: BMC Biol. 2022 Jun 27;20:151. doi: 10.1186/s12915-022-01317-z (PMC9238271; doi:10.1186/s12915-022-01317-z)

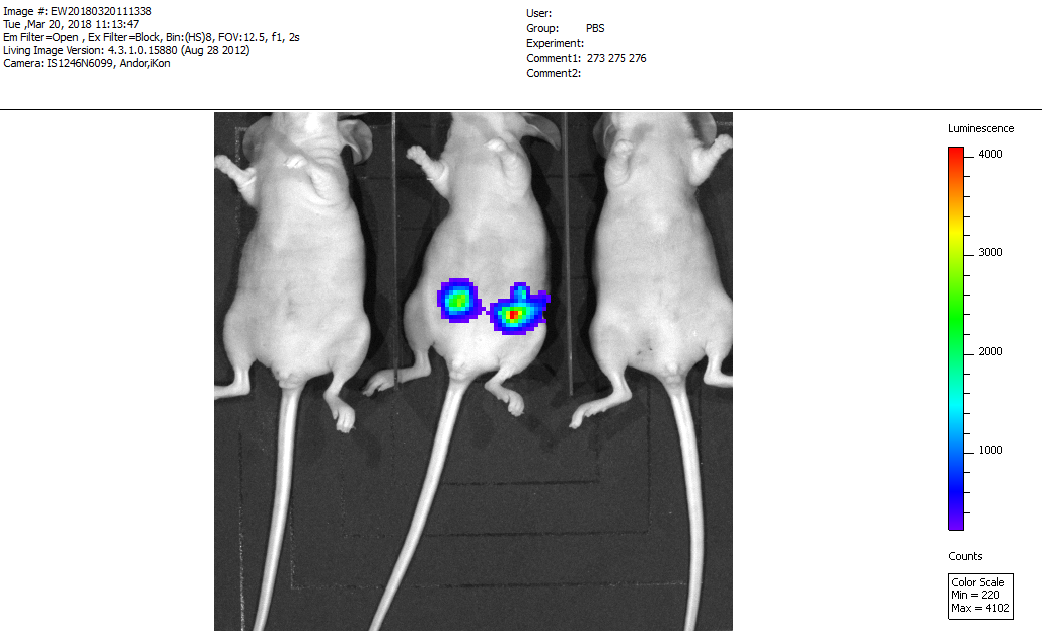

Supplement: Supplementary file 16 — Additional file 16. Raw data bioluminescence files. [file 12915_2022_1317_MOESM16_ESM.zip › Bioluminescence/Raw_Data/20180320/EW20180320111338/EW20180320111338.PNG]

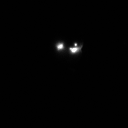

Supplement: Supplementary file 16 — Additional file 16. Raw data bioluminescence files. [file 12915_2022_1317_MOESM16_ESM.zip › Bioluminescence/Raw_Data/20180320/EW20180320111338/luminescent.TIF]

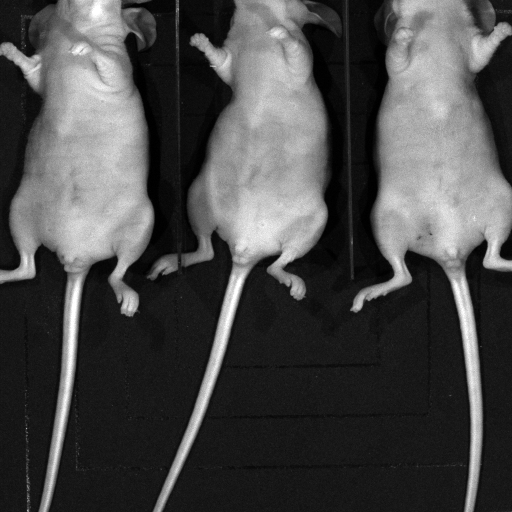

Supplement: Supplementary file 16 — Additional file 16. Raw data bioluminescence files. [file 12915_2022_1317_MOESM16_ESM.zip › Bioluminescence/Raw_Data/20180320/EW20180320111338/photograph.TIF]

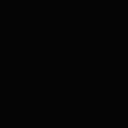

Supplement: Supplementary file 16 — Additional file 16. Raw data bioluminescence files. [file 12915_2022_1317_MOESM16_ESM.zip › Bioluminescence/Raw_Data/20180320/EW20180320111338/readbiasonly.TIF]

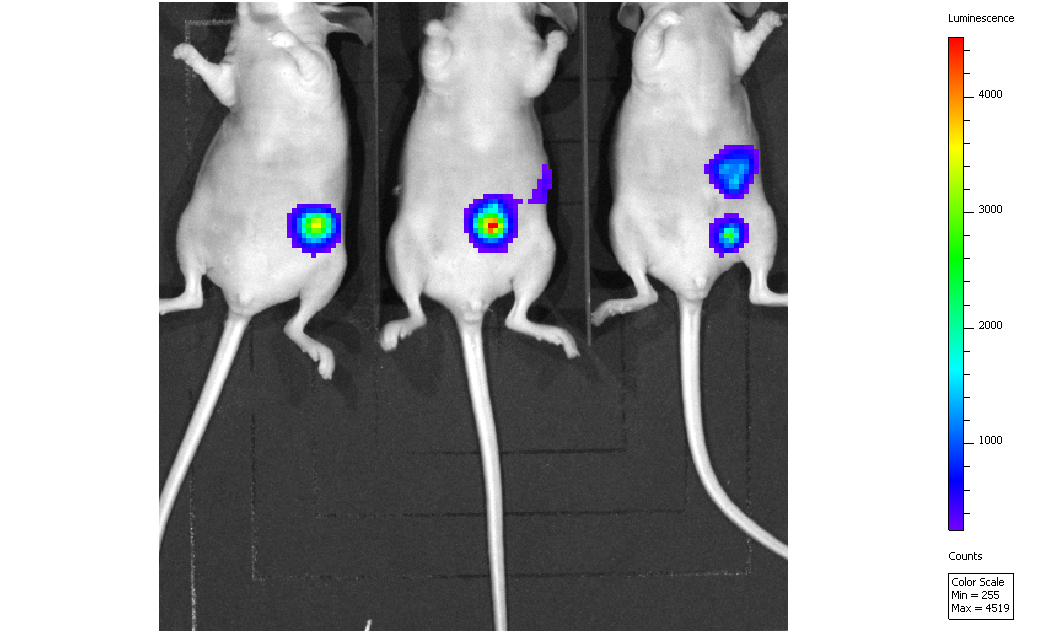

Supplement: Supplementary file 16 — Additional file 16. Raw data bioluminescence files. [file 12915_2022_1317_MOESM16_ESM.zip › Bioluminescence/Raw_Data/20180320/EW20180320112849/EW20180320112849.PNG]

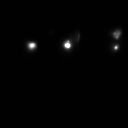

Supplement: Supplementary file 16 — Additional file 16. Raw data bioluminescence files. [file 12915_2022_1317_MOESM16_ESM.zip › Bioluminescence/Raw_Data/20180320/EW20180320112849/luminescent.TIF]

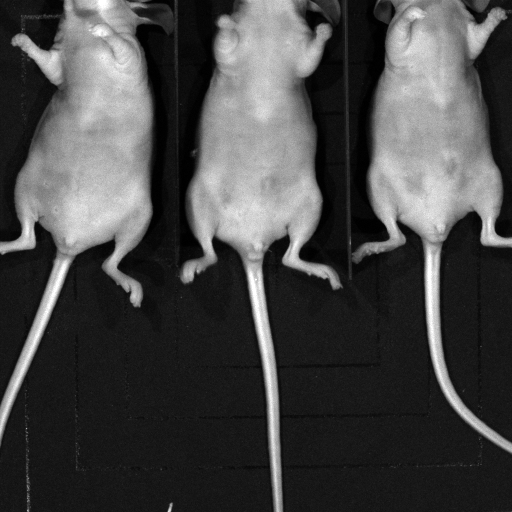

Supplement: Supplementary file 16 — Additional file 16. Raw data bioluminescence files. [file 12915_2022_1317_MOESM16_ESM.zip › Bioluminescence/Raw_Data/20180320/EW20180320112849/photograph.TIF]

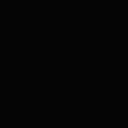

Supplement: Supplementary file 16 — Additional file 16. Raw data bioluminescence files. [file 12915_2022_1317_MOESM16_ESM.zip › Bioluminescence/Raw_Data/20180320/EW20180320112849/readbiasonly.TIF]

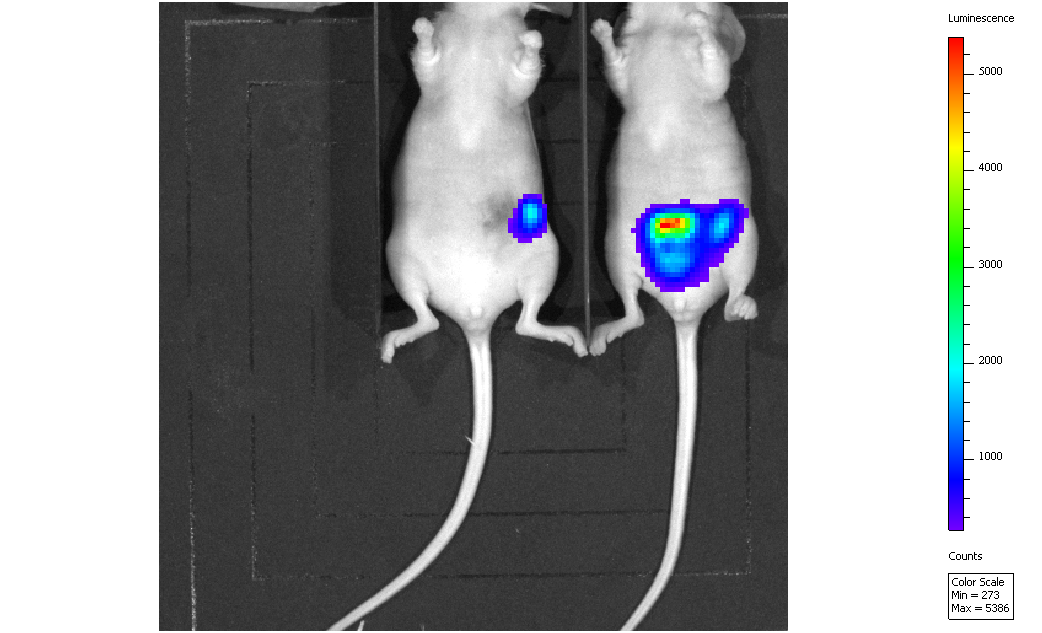

Supplement: Supplementary file 16 — Additional file 16. Raw data bioluminescence files. [file 12915_2022_1317_MOESM16_ESM.zip › Bioluminescence/Raw_Data/20180320/EW20180320113910/EW20180320113910.PNG]

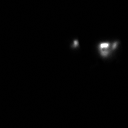

Supplement: Supplementary file 16 — Additional file 16. Raw data bioluminescence files. [file 12915_2022_1317_MOESM16_ESM.zip › Bioluminescence/Raw_Data/20180320/EW20180320113910/luminescent.TIF]

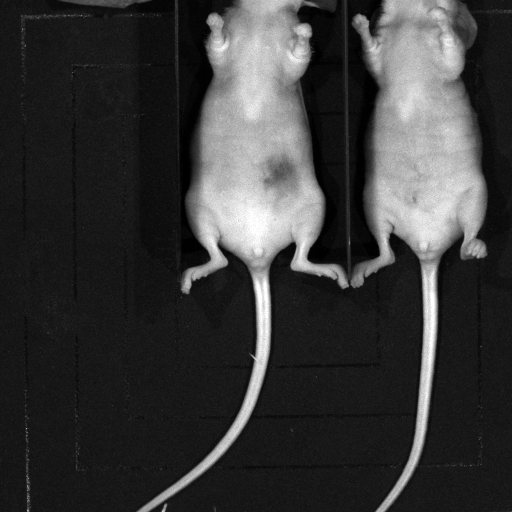

Supplement: Supplementary file 16 — Additional file 16. Raw data bioluminescence files. [file 12915_2022_1317_MOESM16_ESM.zip › Bioluminescence/Raw_Data/20180320/EW20180320113910/photograph.TIF]

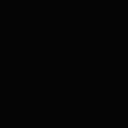

Supplement: Supplementary file 16 — Additional file 16. Raw data bioluminescence files. [file 12915_2022_1317_MOESM16_ESM.zip › Bioluminescence/Raw_Data/20180320/EW20180320113910/readbiasonly.TIF]

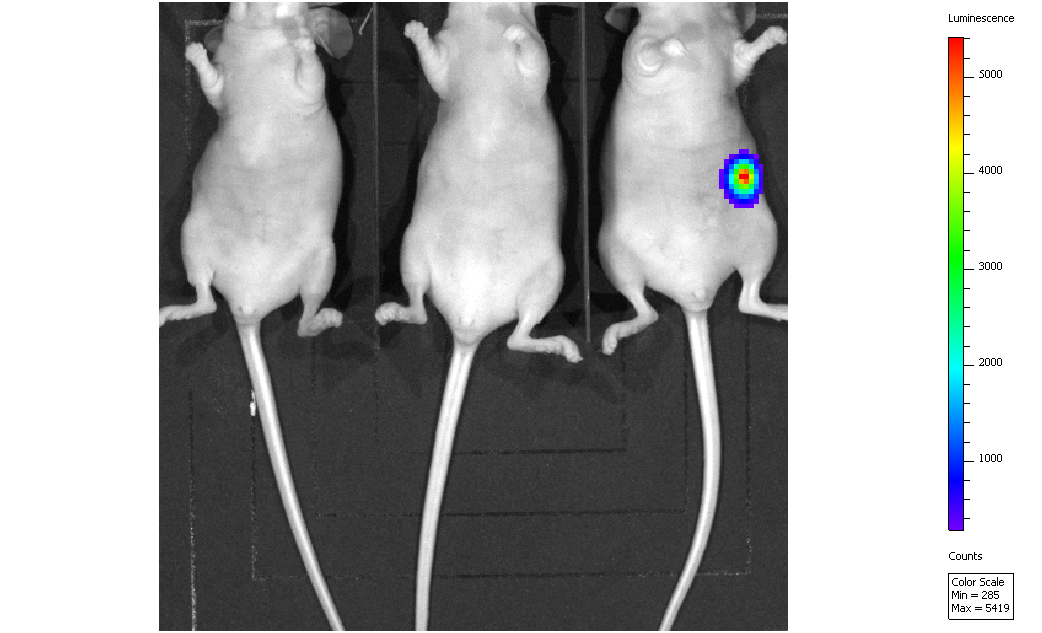

Supplement: Supplementary file 16 — Additional file 16. Raw data bioluminescence files. [file 12915_2022_1317_MOESM16_ESM.zip › Bioluminescence/Raw_Data/20180320/EW20180320114726/EW20180320114726.PNG]

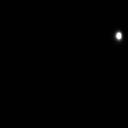

Supplement: Supplementary file 16 — Additional file 16. Raw data bioluminescence files. [file 12915_2022_1317_MOESM16_ESM.zip › Bioluminescence/Raw_Data/20180320/EW20180320114726/luminescent.TIF]

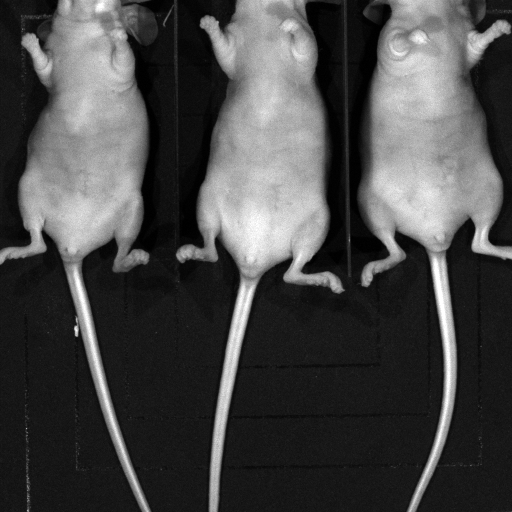

Supplement: Supplementary file 16 — Additional file 16. Raw data bioluminescence files. [file 12915_2022_1317_MOESM16_ESM.zip › Bioluminescence/Raw_Data/20180320/EW20180320114726/photograph.TIF]

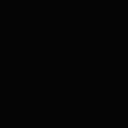

Supplement: Supplementary file 16 — Additional file 16. Raw data bioluminescence files. [file 12915_2022_1317_MOESM16_ESM.zip › Bioluminescence/Raw_Data/20180320/EW20180320114726/readbiasonly.TIF]

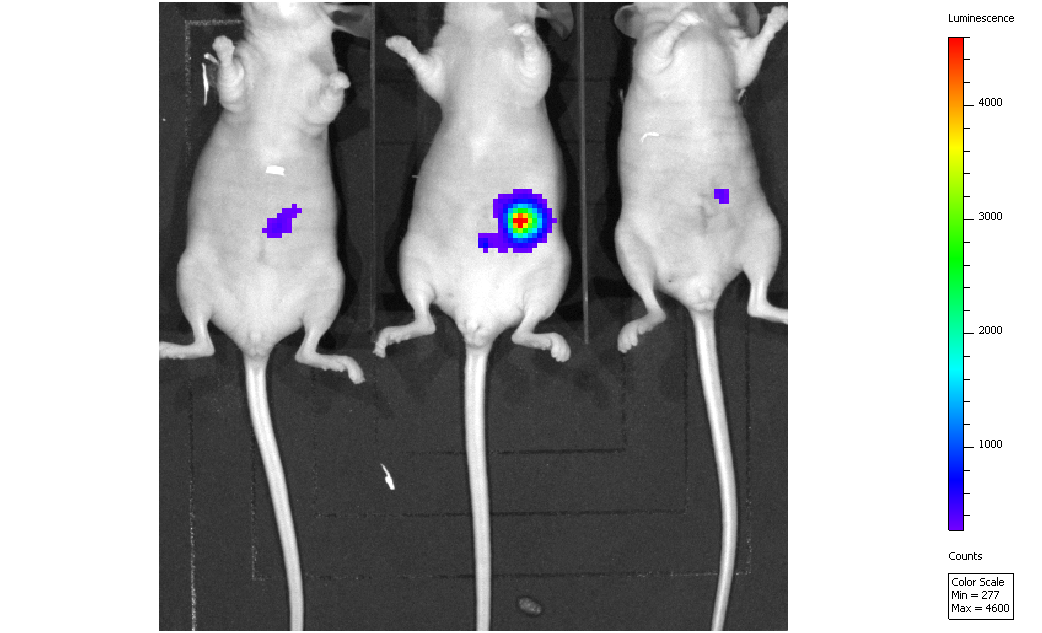

Supplement: Supplementary file 16 — Additional file 16. Raw data bioluminescence files. [file 12915_2022_1317_MOESM16_ESM.zip › Bioluminescence/Raw_Data/20180320/EW20180320115548/EW20180320115548.PNG]

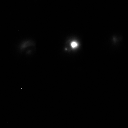

Supplement: Supplementary file 16 — Additional file 16. Raw data bioluminescence files. [file 12915_2022_1317_MOESM16_ESM.zip › Bioluminescence/Raw_Data/20180320/EW20180320115548/luminescent.TIF]

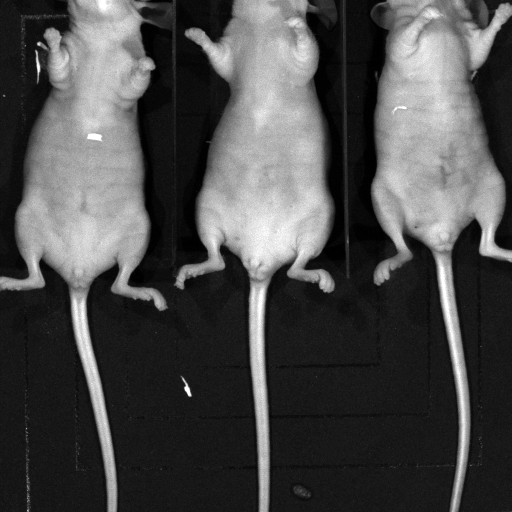

Supplement: Supplementary file 16 — Additional file 16. Raw data bioluminescence files. [file 12915_2022_1317_MOESM16_ESM.zip › Bioluminescence/Raw_Data/20180320/EW20180320115548/photograph.TIF]

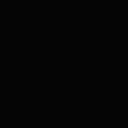

Supplement: Supplementary file 16 — Additional file 16. Raw data bioluminescence files. [file 12915_2022_1317_MOESM16_ESM.zip › Bioluminescence/Raw_Data/20180320/EW20180320115548/readbiasonly.TIF]

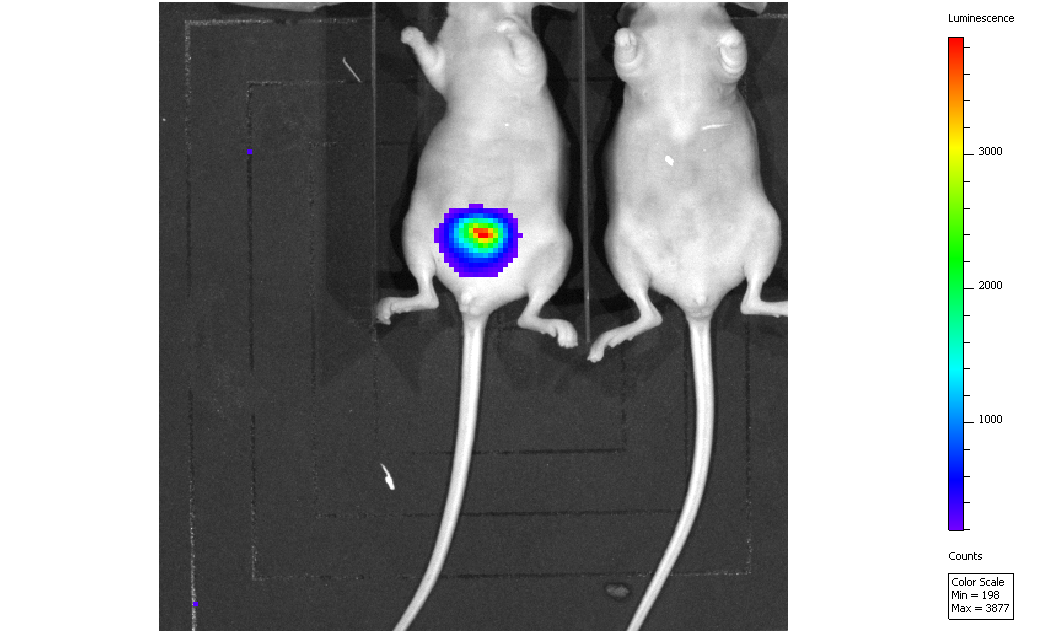

Supplement: Supplementary file 16 — Additional file 16. Raw data bioluminescence files. [file 12915_2022_1317_MOESM16_ESM.zip › Bioluminescence/Raw_Data/20180320/EW20180320120212/EW20180320120212.PNG]

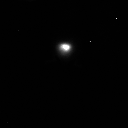

Supplement: Supplementary file 16 — Additional file 16. Raw data bioluminescence files. [file 12915_2022_1317_MOESM16_ESM.zip › Bioluminescence/Raw_Data/20180320/EW20180320120212/luminescent.TIF]

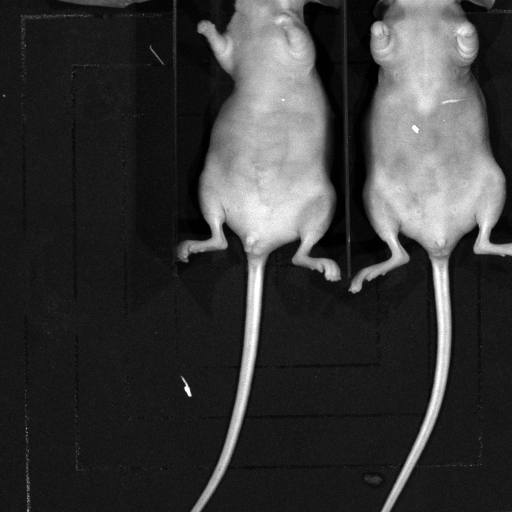

Supplement: Supplementary file 16 — Additional file 16. Raw data bioluminescence files. [file 12915_2022_1317_MOESM16_ESM.zip › Bioluminescence/Raw_Data/20180320/EW20180320120212/photograph.TIF]

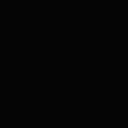

Supplement: Supplementary file 16 — Additional file 16. Raw data bioluminescence files. [file 12915_2022_1317_MOESM16_ESM.zip › Bioluminescence/Raw_Data/20180320/EW20180320120212/readbiasonly.TIF]

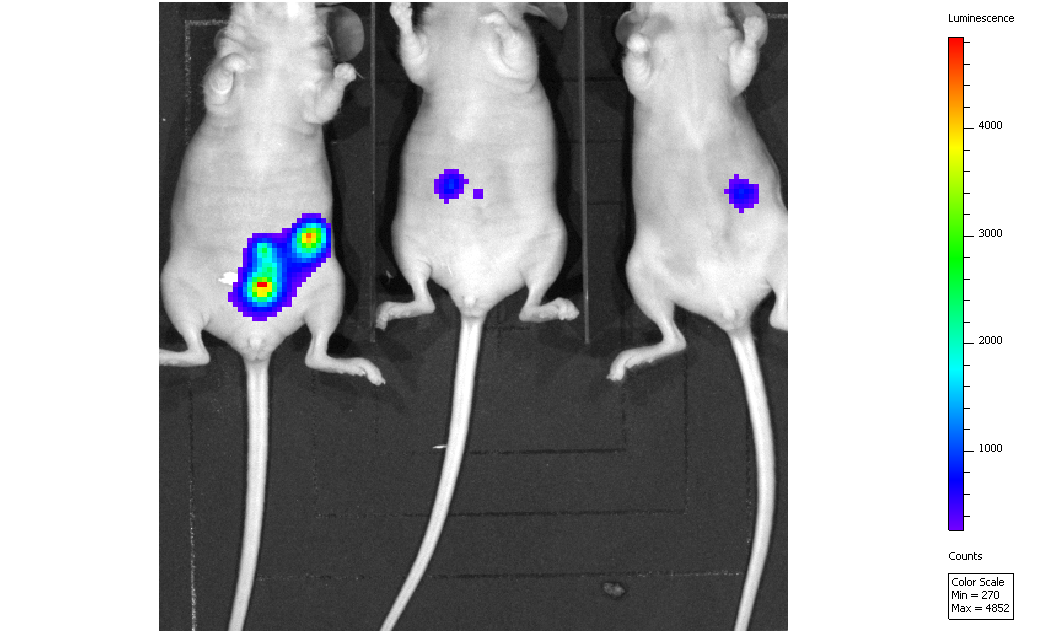

Supplement: Supplementary file 16 — Additional file 16. Raw data bioluminescence files. [file 12915_2022_1317_MOESM16_ESM.zip › Bioluminescence/Raw_Data/20180320/EW20180320121816/EW20180320121816.PNG]

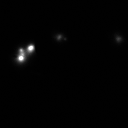

Supplement: Supplementary file 16 — Additional file 16. Raw data bioluminescence files. [file 12915_2022_1317_MOESM16_ESM.zip › Bioluminescence/Raw_Data/20180320/EW20180320121816/luminescent.TIF]

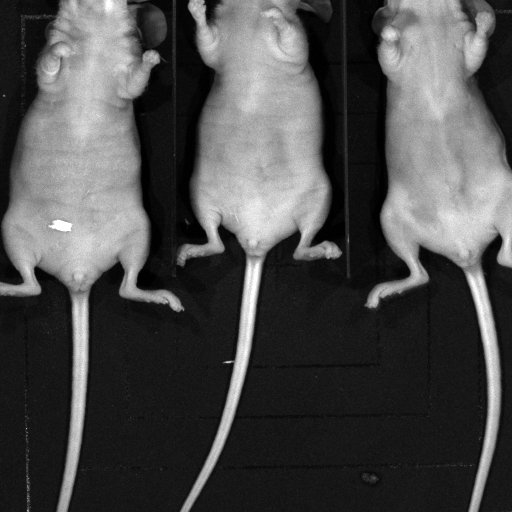

Supplement: Supplementary file 16 — Additional file 16. Raw data bioluminescence files. [file 12915_2022_1317_MOESM16_ESM.zip › Bioluminescence/Raw_Data/20180320/EW20180320121816/photograph.TIF]

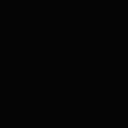

Supplement: Supplementary file 16 — Additional file 16. Raw data bioluminescence files. [file 12915_2022_1317_MOESM16_ESM.zip › Bioluminescence/Raw_Data/20180320/EW20180320121816/readbiasonly.TIF]

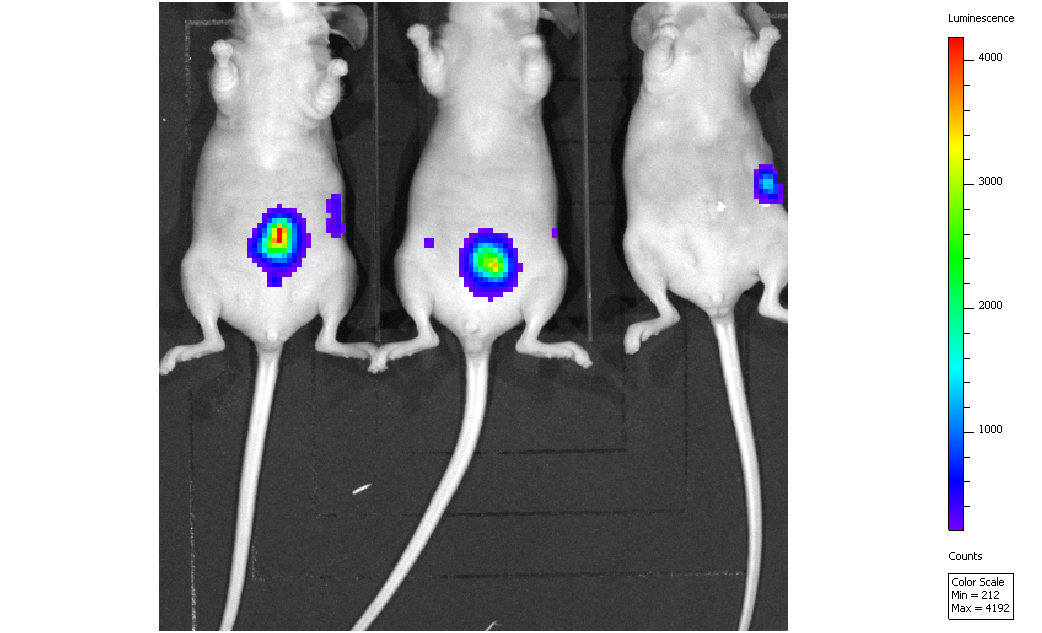

Supplement: Supplementary file 16 — Additional file 16. Raw data bioluminescence files. [file 12915_2022_1317_MOESM16_ESM.zip › Bioluminescence/Raw_Data/20180320/EW20180320122519/EW20180320122519.PNG]

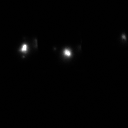

Supplement: Supplementary file 16 — Additional file 16. Raw data bioluminescence files. [file 12915_2022_1317_MOESM16_ESM.zip › Bioluminescence/Raw_Data/20180320/EW20180320122519/luminescent.TIF]

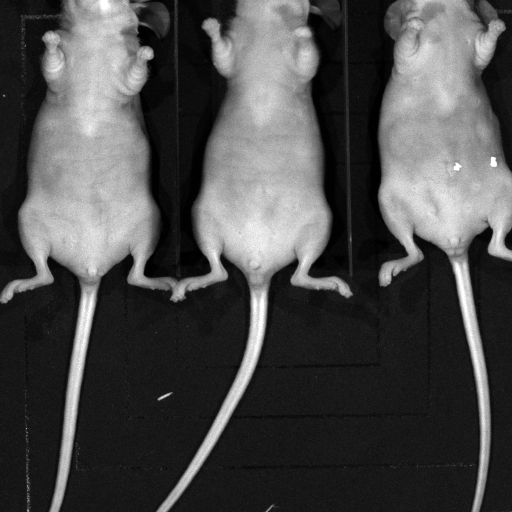

Supplement: Supplementary file 16 — Additional file 16. Raw data bioluminescence files. [file 12915_2022_1317_MOESM16_ESM.zip › Bioluminescence/Raw_Data/20180320/EW20180320122519/photograph.TIF]

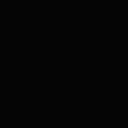

Supplement: Supplementary file 16 — Additional file 16. Raw data bioluminescence files. [file 12915_2022_1317_MOESM16_ESM.zip › Bioluminescence/Raw_Data/20180320/EW20180320122519/readbiasonly.TIF]

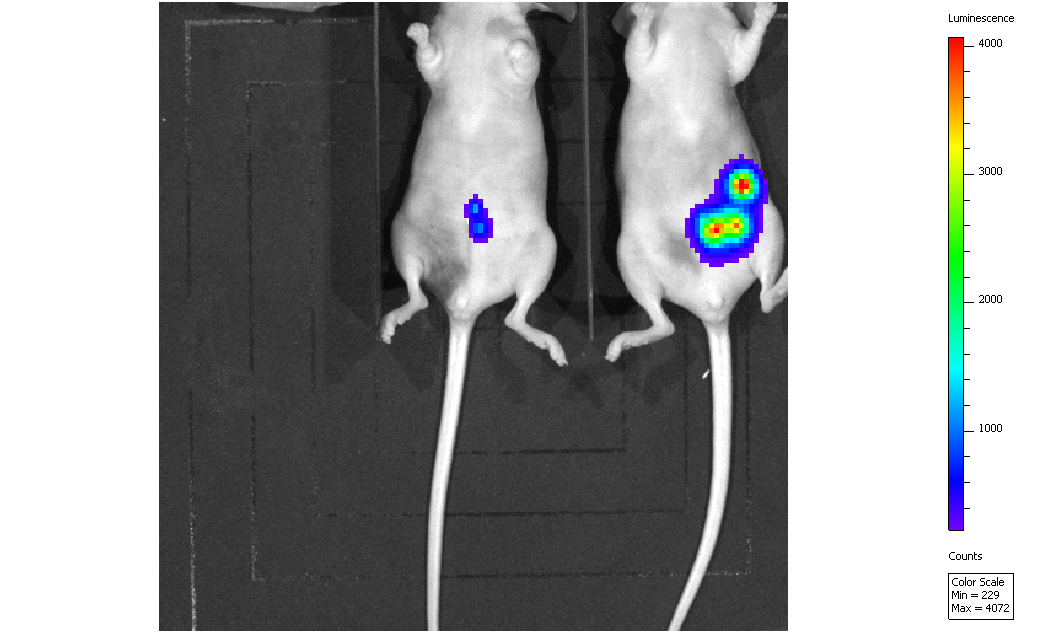

Supplement: Supplementary file 16 — Additional file 16. Raw data bioluminescence files. [file 12915_2022_1317_MOESM16_ESM.zip › Bioluminescence/Raw_Data/20180320/EW20180320123944/EW20180320123944.PNG]

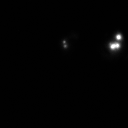

Supplement: Supplementary file 16 — Additional file 16. Raw data bioluminescence files. [file 12915_2022_1317_MOESM16_ESM.zip › Bioluminescence/Raw_Data/20180320/EW20180320123944/luminescent.TIF]

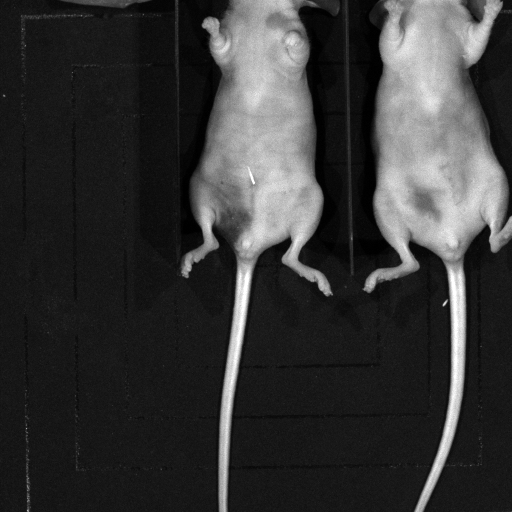

Supplement: Supplementary file 16 — Additional file 16. Raw data bioluminescence files. [file 12915_2022_1317_MOESM16_ESM.zip › Bioluminescence/Raw_Data/20180320/EW20180320123944/photograph.TIF]

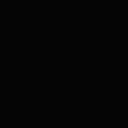

Supplement: Supplementary file 16 — Additional file 16. Raw data bioluminescence files. [file 12915_2022_1317_MOESM16_ESM.zip › Bioluminescence/Raw_Data/20180320/EW20180320123944/readbiasonly.TIF]

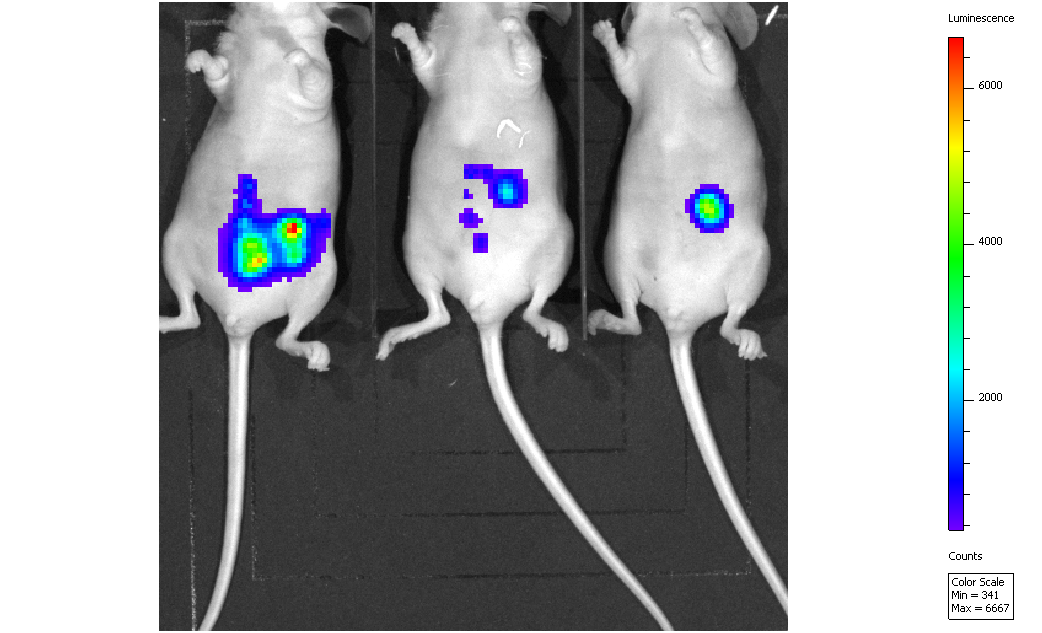

Supplement: Supplementary file 16 — Additional file 16. Raw data bioluminescence files. [file 12915_2022_1317_MOESM16_ESM.zip › Bioluminescence/Raw_Data/20180320/EW20180320124824/EW20180320124824.PNG]

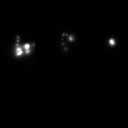

Supplement: Supplementary file 16 — Additional file 16. Raw data bioluminescence files. [file 12915_2022_1317_MOESM16_ESM.zip › Bioluminescence/Raw_Data/20180320/EW20180320124824/luminescent.TIF]

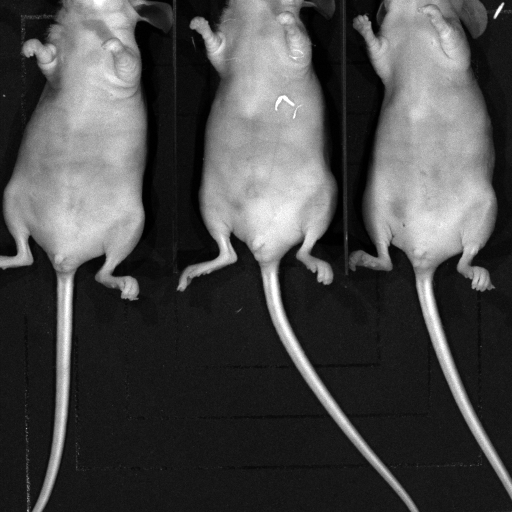

Supplement: Supplementary file 16 — Additional file 16. Raw data bioluminescence files. [file 12915_2022_1317_MOESM16_ESM.zip › Bioluminescence/Raw_Data/20180320/EW20180320124824/photograph.TIF]

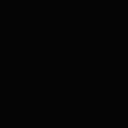

Supplement: Supplementary file 16 — Additional file 16. Raw data bioluminescence files. [file 12915_2022_1317_MOESM16_ESM.zip › Bioluminescence/Raw_Data/20180320/EW20180320124824/readbiasonly.TIF]

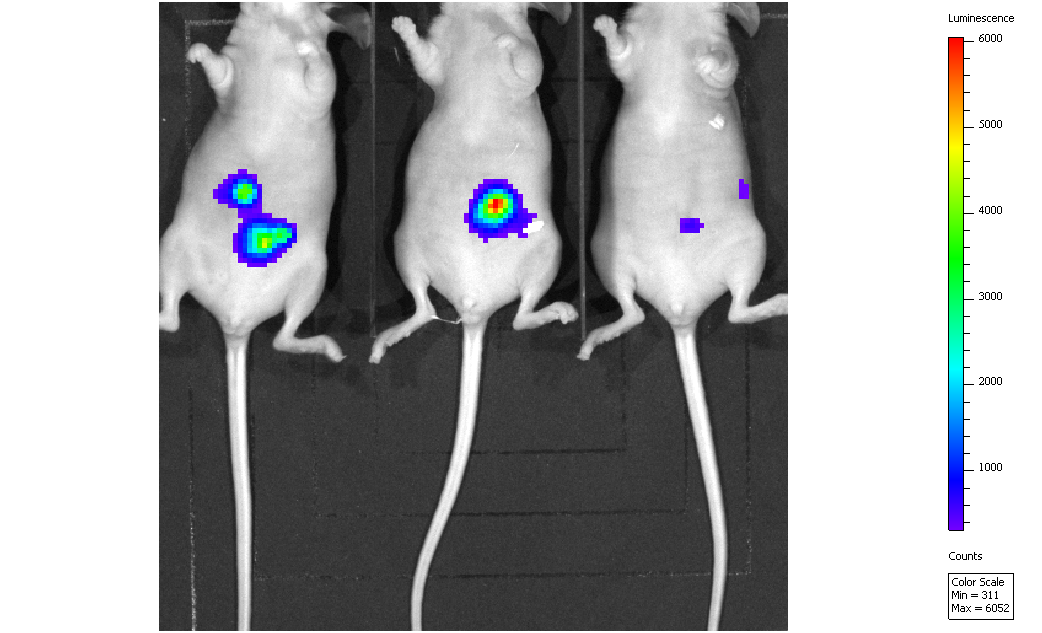

Supplement: Supplementary file 16 — Additional file 16. Raw data bioluminescence files. [file 12915_2022_1317_MOESM16_ESM.zip › Bioluminescence/Raw_Data/20180320/EW20180320125735/EW20180320125735.PNG]

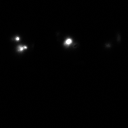

Supplement: Supplementary file 16 — Additional file 16. Raw data bioluminescence files. [file 12915_2022_1317_MOESM16_ESM.zip › Bioluminescence/Raw_Data/20180320/EW20180320125735/luminescent.TIF]

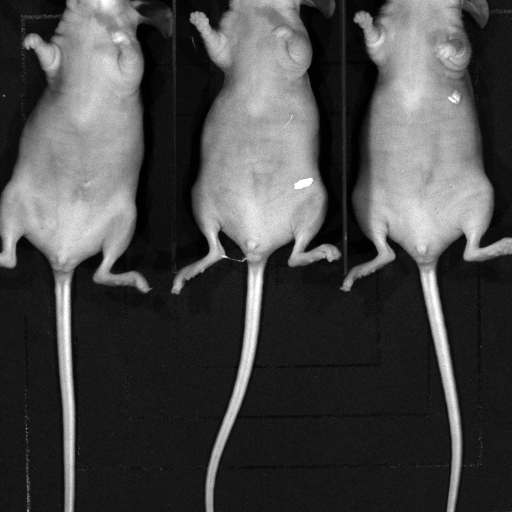

Supplement: Supplementary file 16 — Additional file 16. Raw data bioluminescence files. [file 12915_2022_1317_MOESM16_ESM.zip › Bioluminescence/Raw_Data/20180320/EW20180320125735/photograph.TIF]

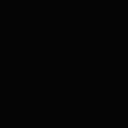

Supplement: Supplementary file 16 — Additional file 16. Raw data bioluminescence files. [file 12915_2022_1317_MOESM16_ESM.zip › Bioluminescence/Raw_Data/20180320/EW20180320125735/readbiasonly.TIF]

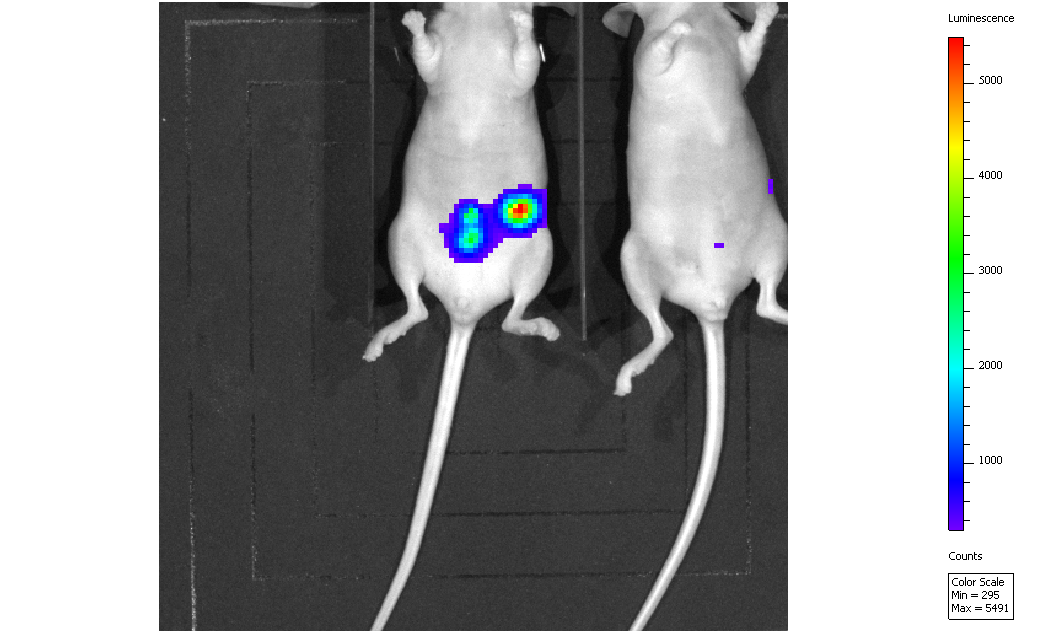

Supplement: Supplementary file 16 — Additional file 16. Raw data bioluminescence files. [file 12915_2022_1317_MOESM16_ESM.zip › Bioluminescence/Raw_Data/20180320/EW20180320130505/EW20180320130505.PNG]

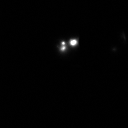

Supplement: Supplementary file 16 — Additional file 16. Raw data bioluminescence files. [file 12915_2022_1317_MOESM16_ESM.zip › Bioluminescence/Raw_Data/20180320/EW20180320130505/luminescent.TIF]

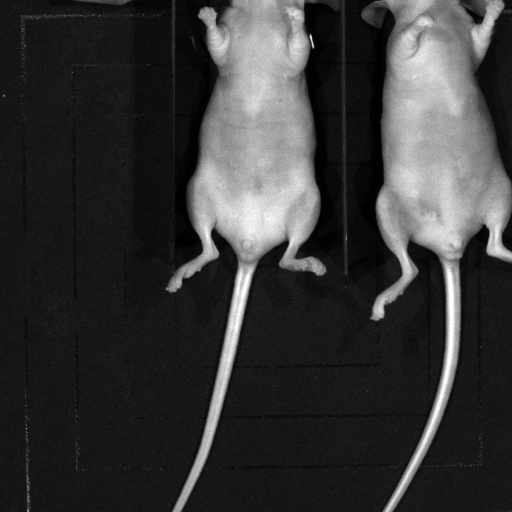

Supplement: Supplementary file 16 — Additional file 16. Raw data bioluminescence files. [file 12915_2022_1317_MOESM16_ESM.zip › Bioluminescence/Raw_Data/20180320/EW20180320130505/photograph.TIF]

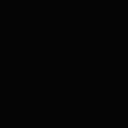

Supplement: Supplementary file 16 — Additional file 16. Raw data bioluminescence files. [file 12915_2022_1317_MOESM16_ESM.zip › Bioluminescence/Raw_Data/20180320/EW20180320130505/readbiasonly.TIF]

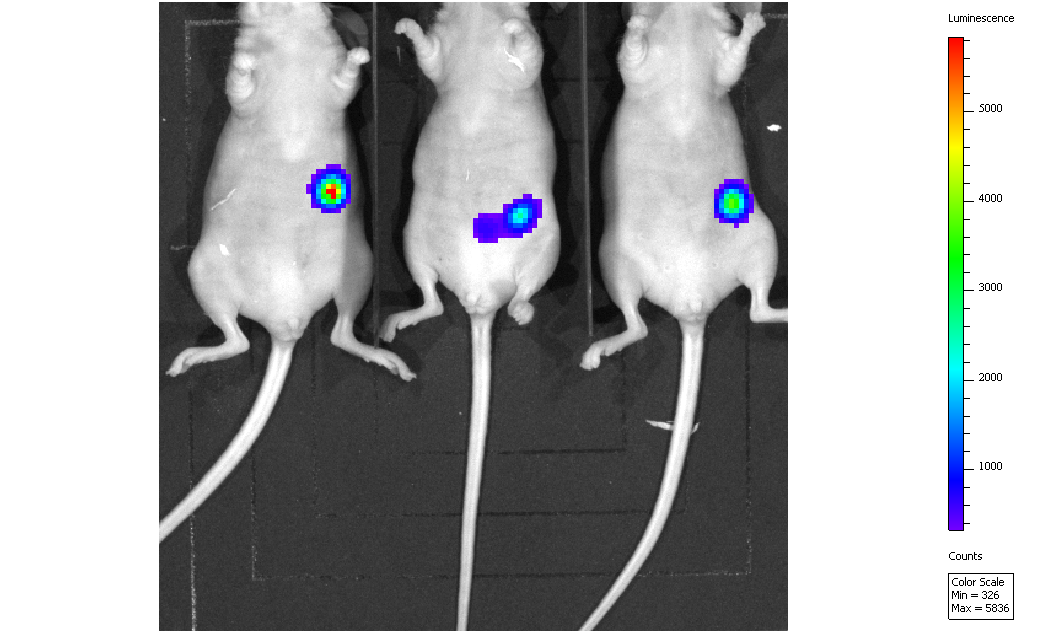

Supplement: Supplementary file 16 — Additional file 16. Raw data bioluminescence files. [file 12915_2022_1317_MOESM16_ESM.zip › Bioluminescence/Raw_Data/20180320/EW20180320131942/EW20180320131942.PNG]

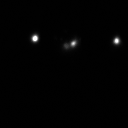

Supplement: Supplementary file 16 — Additional file 16. Raw data bioluminescence files. [file 12915_2022_1317_MOESM16_ESM.zip › Bioluminescence/Raw_Data/20180320/EW20180320131942/luminescent.TIF]

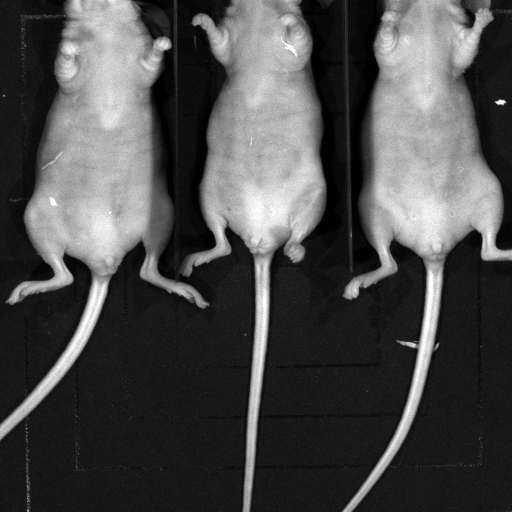

Supplement: Supplementary file 16 — Additional file 16. Raw data bioluminescence files. [file 12915_2022_1317_MOESM16_ESM.zip › Bioluminescence/Raw_Data/20180320/EW20180320131942/photograph.TIF]

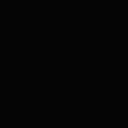

Supplement: Supplementary file 16 — Additional file 16. Raw data bioluminescence files. [file 12915_2022_1317_MOESM16_ESM.zip › Bioluminescence/Raw_Data/20180320/EW20180320131942/readbiasonly.TIF]

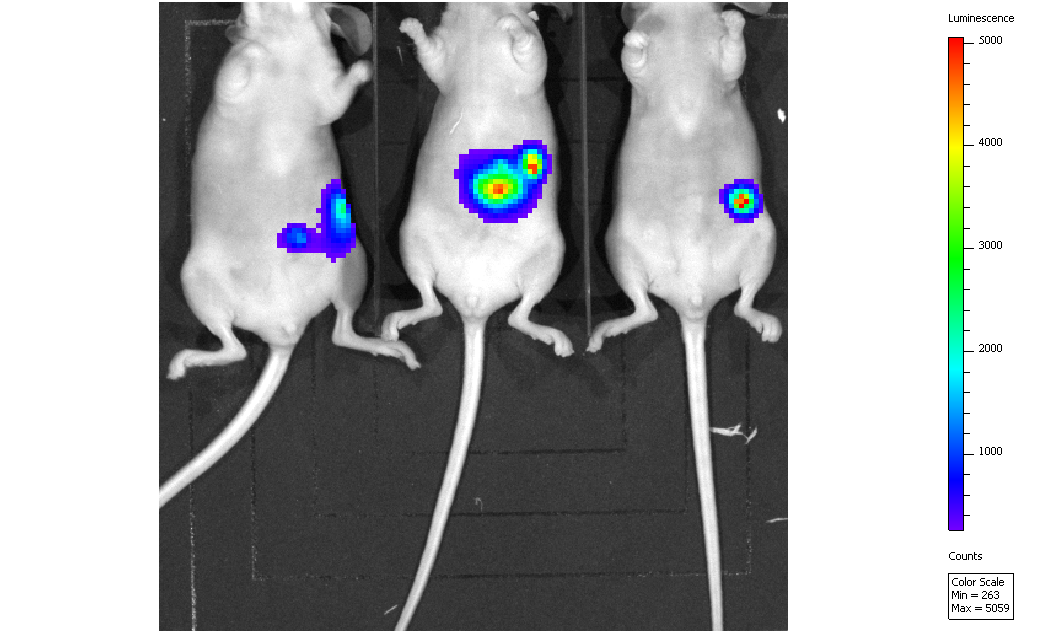

Supplement: Supplementary file 16 — Additional file 16. Raw data bioluminescence files. [file 12915_2022_1317_MOESM16_ESM.zip › Bioluminescence/Raw_Data/20180320/EW20180320132743/EW20180320132743.PNG]

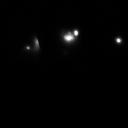

Supplement: Supplementary file 16 — Additional file 16. Raw data bioluminescence files. [file 12915_2022_1317_MOESM16_ESM.zip › Bioluminescence/Raw_Data/20180320/EW20180320132743/luminescent.TIF]

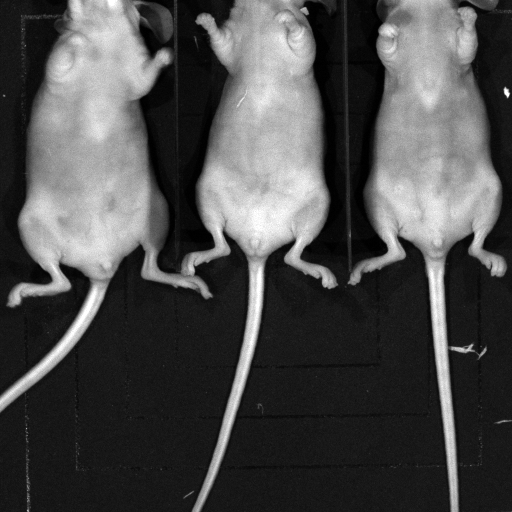

Supplement: Supplementary file 16 — Additional file 16. Raw data bioluminescence files. [file 12915_2022_1317_MOESM16_ESM.zip › Bioluminescence/Raw_Data/20180320/EW20180320132743/photograph.TIF]

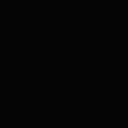

Supplement: Supplementary file 16 — Additional file 16. Raw data bioluminescence files. [file 12915_2022_1317_MOESM16_ESM.zip › Bioluminescence/Raw_Data/20180320/EW20180320132743/readbiasonly.TIF]

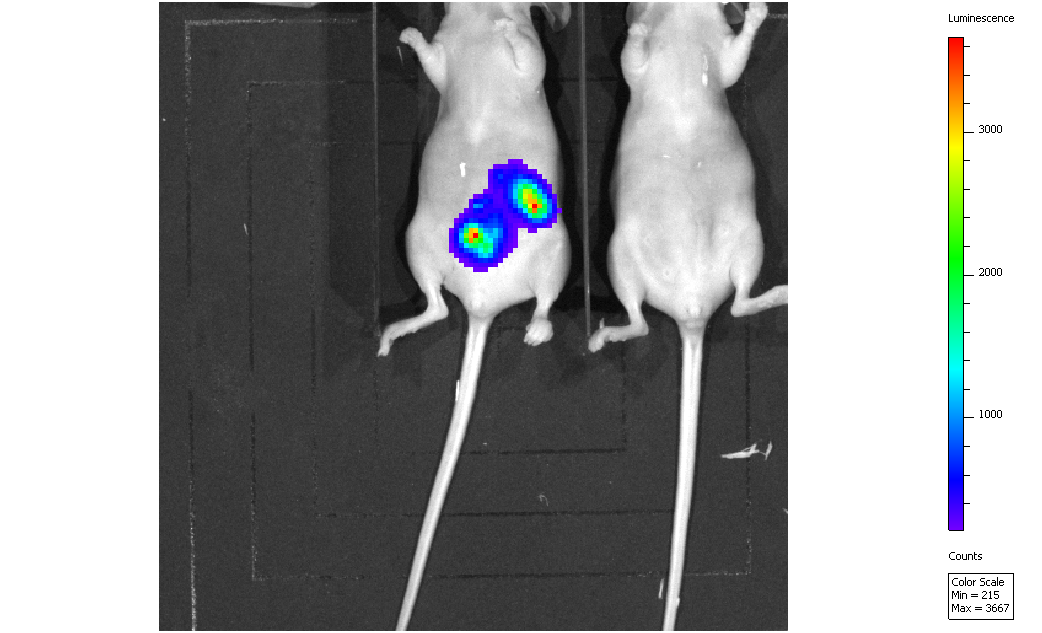

Supplement: Supplementary file 16 — Additional file 16. Raw data bioluminescence files. [file 12915_2022_1317_MOESM16_ESM.zip › Bioluminescence/Raw_Data/20180320/EW20180320133409/EW20180320133409.PNG]

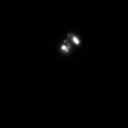

Supplement: Supplementary file 16 — Additional file 16. Raw data bioluminescence files. [file 12915_2022_1317_MOESM16_ESM.zip › Bioluminescence/Raw_Data/20180320/EW20180320133409/luminescent.TIF]

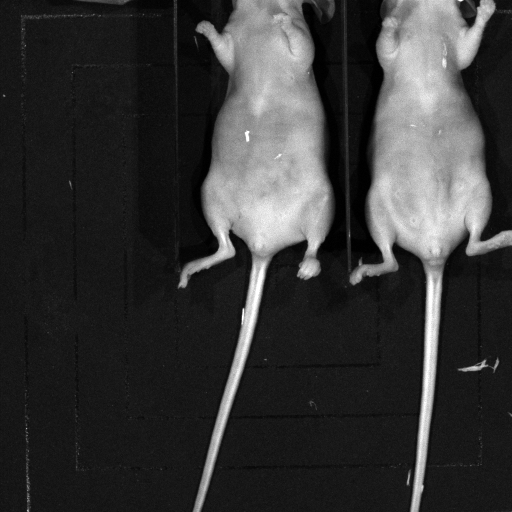

Supplement: Supplementary file 16 — Additional file 16. Raw data bioluminescence files. [file 12915_2022_1317_MOESM16_ESM.zip › Bioluminescence/Raw_Data/20180320/EW20180320133409/photograph.TIF]

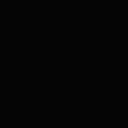

Supplement: Supplementary file 16 — Additional file 16. Raw data bioluminescence files. [file 12915_2022_1317_MOESM16_ESM.zip › Bioluminescence/Raw_Data/20180320/EW20180320133409/readbiasonly.TIF]

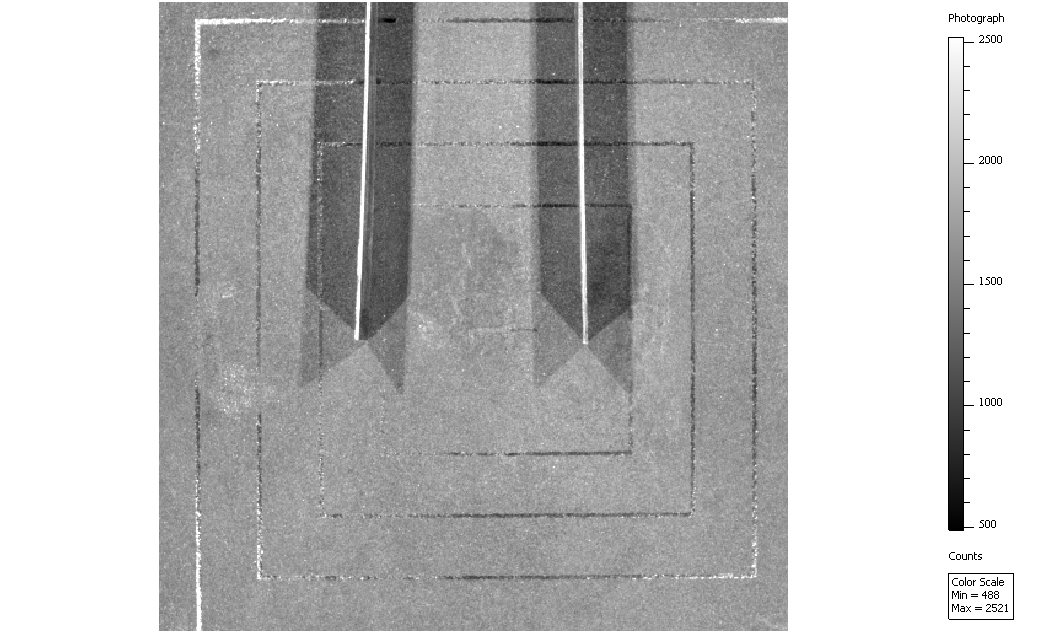

Supplement: Supplementary file 16 — Additional file 16. Raw data bioluminescence files. [file 12915_2022_1317_MOESM16_ESM.zip › Bioluminescence/Raw_Data/20180327/EW20180327111602/EW20180327111602.PNG]

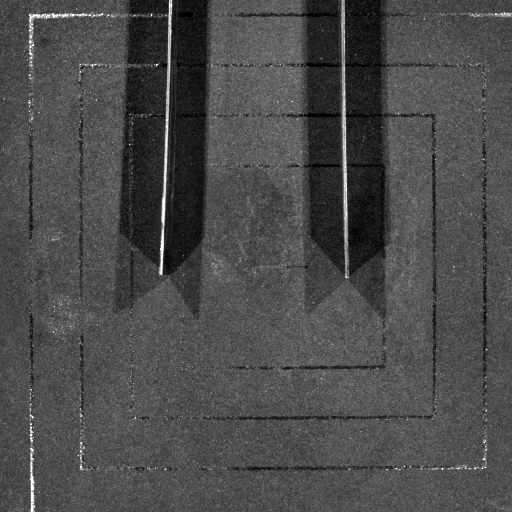

Supplement: Supplementary file 16 — Additional file 16. Raw data bioluminescence files. [file 12915_2022_1317_MOESM16_ESM.zip › Bioluminescence/Raw_Data/20180327/EW20180327111602/photograph.TIF]
