# Supplementary material for: The quorum sensing peptide EntF* promotes colorectal cancer metastasis in mice: a new factor in the host-microbiome interaction
Source: BMC Biol. 2022 Jun 27;20:151. doi: 10.1186/s12915-022-01317-z (PMC9238271; doi:10.1186/s12915-022-01317-z)
Supplement: Supplementary file 17 — Additional file 17. Raw data microscopy files. [file 12915_2022_1317_MOESM17_ESM.docx]

Link to microscopy images (liver and lungs):

<https://ugentbe-my.sharepoint.com/:f:/g/personal/ejwynend_wynendaele_ugent_be/EkOnJ8RafaNJkMEq2lmIW4sBbSvT_bLGUf55vaqCQpG11Q?e=PERHeu>
